# The amide derivative of anticopalic acid induces non-apoptotic cell death in triple-negative breast cancer cells by inhibiting FAK activation

**DOI:** 10.1038/s41598-023-40669-6

**Published:** 2023-08-18

**Authors:** Pornsuda Chawengrum, Natthaorn Luepongpatthana, Sanit Thongnest, Jitnapa Sirirak, Jutatip Boonsombat, Kriengsak Lirdprapamongkol, Siriporn Keeratichamroen, Patcharin Kongwaen, Phreeranat Montatip, Prasat Kittakoop, Jisnuson Svasti, Somsak Ruchirawat

**Affiliations:** 1 Chemical Biology Program, Chulabhorn Graduate Institute, Chulabhorn Royal Academy, Bangkok, Thailand; 2 Applied Biological Sciences Program, Chulabhorn Graduate Institute, Chulabhorn Royal Academy, Bangkok, Thailand; 3 Laboratory of Natural Products, Chulabhorn Research Institute, Bangkok, Thailand; 4 Center of Excellence on Environmental Health and Toxicology (EHT), Office of the Permanent Secretary (OPS), Ministry of Higher Education, Science, Research and Innovation (MHESI), Bangkok, Thailand; 5 Department of Chemistry, Faculty of Science, Silpakorn University, Nakhon Pathom, Thailand; 6 Laboratory of Biochemistry, Chulabhorn Research Institute, Bangkok, Thailand

**Keywords:** Drug discovery, Cancer, Breast cancer, Cancer screening, Chemical modification, Biochemistry, Organic chemistry, Medicinal chemistry, Drug discovery and development

## Abstract

Anticopalic acid (**ACP**), a labdane type diterpenoid obtained from *Kaempferia elegans* rhizomes, together with 21 semi-synthetic derivatives, were evaluated for their cancer cytotoxic activity. Most derivatives displayed higher cytotoxic activity than the parent compound **ACP** in a panel of nine cancer cell lines. Among the tested compounds, the amide **4p** showed the highest cytotoxic activity toward leukemia cell lines, HL-60 and MOLT-3, with IC_50_ values of 6.81 ± 1.99 and 3.72 ± 0.26 µM, respectively. More interestingly, the amide derivative **4l** exhibited cytotoxic activity with an IC_50_ of 13.73 ± 0.04 µM against the MDA-MB-231 triple-negative breast cancer cell line, which is the most aggressive type of breast cancer. Mechanistic studies revealed that **4l** induced cell death in MDA-MB-231 cells through non-apoptotic regulated cell death. In addition, western blot analysis showed that compound **4l** decreased the phosphorylation of FAK protein in a concentration-dependent manner. Molecular docking simulations elucidated that compound **4l** could potentially inhibit FAK activation by binding to a pocket of FAK kinase domain. The data suggested that compound **4l** could be a potential FAK inhibitor for treating triple-negative breast cancer and worth being further investigated.

## Introduction

Cancer is the world’s devastating disease, and breast cancer is the second highest cause of death in all types of cancers^1^. According to the World Health Organization (WHO), around 7.8 million women worldwide were diagnosed with breast cancer in the year 2020^2^. Although uncommon, breast cancer also occurs in male and transgender people^3^. Breast cancer is a highly complex disease classified into many subtypes, each with its own biological features, gene expression profiles, and clinical behaviors^4^. Among breast cancer subtypes, triple-negative breast cancer (TNBC) is the most aggressive and has the highest recurrence rate, metastatic spread, and mortality risks^5^. TNBC is characterized by the lack of expression of estrogen receptor (ER), progesterone receptor (PR), and human epidermal growth factor receptor 2 (HER2). This subtype accounts for approximately 15% of all diagnosed breast cancers and 25% of all deaths related to breast cancer^4^. Due to the lack of suitable targeted receptors, common breast cancer treatments such as hormone therapy and targeted therapy against ER, PR, and HER2 are ineffective in TNBC patients. As a result, TNBC has limited treatment options, and is known to have a worse clinical outcome than other breast cancer subtypes. Chemotherapy remains a standard treatment for TNBC, but chemoresistance frequently occurs^5^. These problems indicate the importance of finding new drugs or efficacious treatment options for TNBC.

Natural products have long made major contributions to drug discovery, especially for cancer and infectious diseases^6,7^. They serve as good sources of new drugs since they are typically affordable materials, having various scaffolds, pharmacophores, and accessibility. In addition, combined with chemical synthesis, natural products can be used as starting points to access diverse molecular structures with amplified bioactivity and desired bioavailability. For these reasons, the concept of isolating a natural product for lead optimization studies or the generation of screening libraries has been regularly reported in the literature^8^. However, to make this strategy effective requires (1) access to adequate supplies for the large-scale isolation of the desired natural product, (2) the high abundance of the natural product of interest from the natural product source, and (3) the presence of chemical functionalities in the natural product scaffold that allows for the simple and high yielding generation of derivatives. Therefore, natural products that could meet these criteria are considered as good sources for a natural product-based drug discovery program.

**Figure 1:**
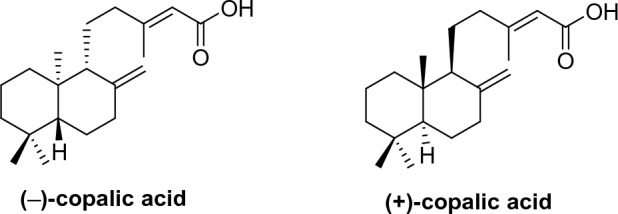
(−)-Copalic acid (COPA) and (+)-copalic acid (anticopalic acid, ACP).

Anticopalic acid (**ACP**) known as (+)-copalic acid (Fig. 1), is a labdane-type bicyclic diterpenoid previously isolated from several plants including *Vitex hemsley*^9^, *Eperua purpurea*^10^, *Eperua leucantha*^11^, *Pinus monticola*^12,13^, *Pinus strobus*^13^, and *Oxystigma oxyphyllum*^14,15^. In 2018, our group isolated **ACP** in large amounts (~ 23%) from the crude extract of the rhizomes of *Kaempferia elegans*^16,17^. Since *Kaempferia elegans* is a plant in the ginger family, which can be easily cultivated, and **ACP** is deemed to have a particularly attractive natural product scaffold due to its low MW (304 Da), multiple stereogenic centers (n = 3, conferring a unique 3D shape), and containing a potential chemical handle, such as carboxylic acid in the molecule. Therefore, **ACP** can be a good candidate as a starting point for optimization into novel natural product-based bioactive agents. It is worth mentioning that anticopalic acid is one of the rare natural products that have both enantiomeric forms found in nature^18^. (−)-Copalic acid (COPA) (Fig. 1), the opposite enantiomeric form of **ACP**, is the major diterpenoid present in folk medicine, copaiba oil^19^.

Based on the comprehensive application of copaiba oil in folk medicine and its recent popularity in the cosmetics and pharmaceutical industries, COPA and its derivatives have been subjected to various scientific studies aiming to prove their biological activities. Previous studies revealed that COPA possesses interesting bioactivities, including antibacterial^20,21^, antifungal^22^, anti-inflammatory^23,24^, anti-tuberculosis^25^ and cancer cytotoxic^26^ properties. Studies of COPA derivatives that offered superior bioactivities than that of the parent COPA have also been reported. For example, the hydrogenated COPA derivative and the COPA derivatives with epoxide, diketone, or sodium carboxylate moieties provided much higher anti-tuberculosis activity^21,25^. Some COPA amide analogues improved their activities in downregulating the expression of androgen receptor for the treatment of prostate cancer^27^.

In contrast to COPA, little is known about the bioactivities of **ACP**. To date, there have only been reports of the antimicrobial activity of **ACP**^16,28^ and antifeedant activity against *Spodoptera frugiperda* of **ACP** and derivatives^9^. In view of the limited existing studies of the bioactivities of **ACP** and the potential use of **ACP** in natural product-based drug discovery, we embarked on the synthesis and cytotoxic evaluation of **ACP** derivatives. Herein, the cytotoxic activity of **ACP** derivatives, mainly the amide analogs, against nine cancer cell lines and the normal cell line MRC-5 was evaluated. Additionally, the molecular mechanisms and biological targets of **ACP** derivative **4l** in MDA-MB-231 triple-negative breast cancer cells were elucidated.

## Results and discussion

### Synthesis of ACP derivatives

**Figure 2:**
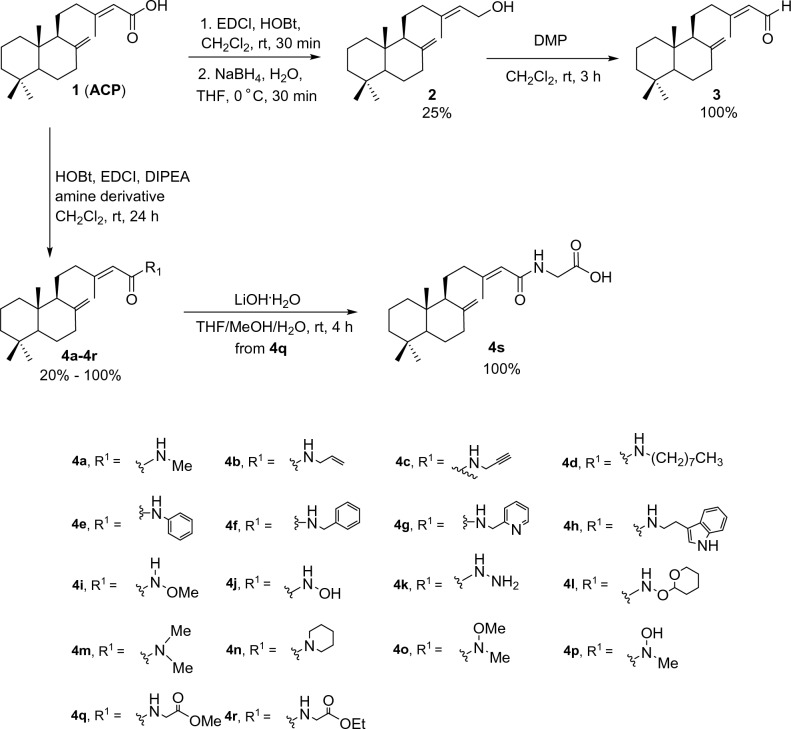
Synthesis of **ACP** derivatives.

The formation of the **ACP** derivatives **1**–**3** and **4a**–**4s** was performed as illustrated in Fig. 2. These included the reduction to the alcohol **2**, the aldehyde **3**, amidation to the amide analogs **4a**–**4r**, and the further hydrolysis of the amide methyl ester **5q** to the acid **4s**. It is noteworthy that most derivatives are amide analogs due to their suitability for quickly performing studies on structure–activity relationship (SAR). In fact, amide formation tends to be the most commonly used by medicinal chemists, and amide bonds are widely present in pharmaceutically active substances, estimated to be found in 25% of marketed drugs^29^.

The yield of the synthesized derivatives was moderate to excellent (20–100%). Of these derivatives, nineteen compounds of the amide derivatives, were reported for the first time. The structures of the synthesized compounds were confirmed by analysis of NMR spectroscopic data and MS. The physical characteristics and spectroscopic data of the synthesized compounds are reported in the Supplementary Information.

### Cytotoxic evaluation revealed the potential of compound 4l towards triple-negative breast cancer MDA-MB-231 cells

The synthetic compounds were screened for their cytotoxic activity using standard MTT or XTT methods^30^ against a panel of nine human cancer cell lines and one normal cell line, including MDA-MB-231 (triple-negative breast cancer), T-47D (hormone-dependent breast cancer), HepG2 (hepatocellular carcinoma), A549 (lung adenocarcinoma), H69AR (multidrug-resistant small cell lung carcinoma), HuCCA-1 (cholangiocarcinoma derived from a Thai patient), HeLa (cervical carcinoma), and two leukemia cell lines, HL-60 (acute promyelocytic leukemia) and MOLT-3 (T cell acute lymphoblastic leukemia). MRC-5 normal embryonic lung fibroblast cell line was used to represent normal cells. Chemotherapeutic drugs, doxorubicin and etoposide, were used as positive controls. IC_50_ values of the tested compounds are given in Table 1.

**Table 1:** Results of cytotoxicity of the isolated compounds towards different human cancer cell lines.

Cpds	Cell lines, IC_50_ (μM)									
	MDA-MB-231^a^	T47-D^b^	HepG2^c^	HL-60^d^	MOLT-3^e^	A549^f^	H69AR^g^	HuCCA-1^h^	HeLa^i^	MRC-5^j^
**1**	I	I	I	103.43 ± 10.71	66.02 ± 0.12	159.13 ± 0.78	I	157.13 ± 0.51	145.17 ± 0.31	I
**2**	31.77 ± 0.13	52.33 ± 0.34	52.33 ± 0.34	27.71 ± 0.31	32.22 ± 1.44	60.52 ± 0.22	111.88 ± 0.71	51.50 ± 0.24	37.49 ± 0.04	54.15 ± 0.31
**3**	64.10 ± 1.80	93.91 ± 1.54	122.16 ± 1.03	56.44 ± 1.37	37.72 ± 3.20	141.78 ± 2.69	I	131.38 ± 1.14	77.44 ± 1.47	125.84 ± 0.46
**4a**	29.92 ± 0.71	73.35 ± 2.06	73.35 ± 2.06	24.72 ± 1.41	25.48 ± 0.70	65.10 ± 3.57	122.48 ± 3.48	62.86 ± 1.36	47.72 ± 1.08	55.90 ± 0.19
**4b**	114.31 ± 0.72	I	106.62 ± 1.28	22.66 ± 0.84	25.41 ± 0.51	I	I	I	79.61 ± 0.49	I
**4c**	27.46 ± 0.62	76.63 ± 4.68	85.06 ± 0.61	24.13 ± 0.08	23.22 ± 1.03	54.05 ± 1.17	112.61 ± 1.35	52.70 ± 0.11	28.06 ± 0.93	48.14 ± 0.62
**4d**	I	I	I	I	I	I	I	I	I	I
**4e**	I	I	I	60.57 ± 4.97	25.95 ± 1.88	46.87 ± 1.28	I	I	98.11 ± 0.08	I
**4f**	I	I	I	87.30 ± 8.47	I	I	I	I	I	I
**4g**	35.05 ± 0.98	55.10 ± 2.80	41.66 ± 1.46	22.66 ± 0.36	20.86 ± 0.99	38.22 ± 0.49	92.73 ± 1.18	46.20 ± 0.60	46.68 ± 1.03	44.81 ± 1.62
**4h**	23.46 ± 0.36	I	40.48 ± 2.25	16.79 ± 0.61	17.10 ± 0.57	41.44 ± 2.48	71.46 ± 2.47	42.36 ± 0.50	20.73 ± 0.40	48.25 ± 1.06
**4i**	45.01 ± 0.86	41.26 ± 1.61	44.26 ± 0.40	24.89 ± 0.45	21.35 ± 1.29	45.67 ± 0.63	96.91 ± 0.46	49.80 ± 0.18	53.76 ± 0.83	52.68 ± 0.03
**4j**	I	I	I	I	I	I	I	I	I	I
**4k**	98.52 ± 3.01	I	I	107.97 ± 1.53	29.29 ± 2.43	I	I	130.02 ± 2.00	118.78 ± 0.06	145.81 ± 0.34
**4l**	13.73 ± 0.04	66.06 ± 6.39	73.49 ± 2.15	21.36 ± 0.31	26.16 ± 1.76	57.71 ± 1.20	94.60 ± 1.67	58.67 ± 0.13	26.44 ± 0.04	40.34 ± 2.11
**4m**	32.15 ± 1.68	42.62 ± 1.08	52.09 ± 2.82	19.42 ± 0.84	21.29 ± 0.47	51.82 ± 0.25	99.97 ± 0.74	44.31 ± 0.42	40.15 ± 0.68	40.78 ± 0.56
**4n**	33.93 ± 1.93	73.01 ± 3.31	32.24 ± 1.37	20.51 ± 0.50	21.04 ± 0.32	41.71 ± 1.43	52.31 ± 0.42	41.25 ± 0.19	38.91 ± 0.13	38.40 ± 1.20
**4o**	38.82 ± 0.44	46.38 ± 1.73	57.29 ± 1.82	27.91 ± 1.99	29.84 ± 1.89	82.44 ± 0.99	115.07 ± 0.64	54.30 ± 1.18	52.86 ± 0.02	47.25 ± 0.96
**4p**	24.50 ± 0.61	35.68 ± 1.01	64.56 ± 0.76	6.81 ± 1.99	3.72 ± 0.26	61.35 ± 2.16	96.67 ± 0.08	58.59 ± 1.20	33.64 ± 0.54	69.80 ± 1.24
**4q**	46.23 ± 1.89	71.26 ± 2.07	73.89 ± 0.30	26.02 ± 0.55	14.86 ± 0.32	43.83 ± 3.08	61.43 ± 2.03	81.11 ± 2.42	39.06 ± 0.78	65.29 ± 0.11
**4r**	23.40 ± 0.81	87.22 ± 6.02	61.30 ± 1.12	23.92 ± 0.59	11.50 ± 1.13	67.33 ± 2.09	45.87 ± 1.80	72.31 ± 1.93	36.63 ± 1.44	63.94 ± 0.65
**4s**	I	I	–	75.07 ± 3.24	84.14 ± 0.09	I	I	I	I	I
**DOX**	2.70 ± 0.16	0.47 ± 0.03	0.40 ± 0.02	–	–	0.38 ± 0.03	38.08 ± 0.29	0.78 ± 0.06	0.48 ± 0.01	0.76 ± 0.05
**ETO**	–	–	–	1.21 ± 0.15	0.07 ± 0.12	–	–	–	–	–

^a^Triple-negative breast cancer.

^b^Hormone-dependent breast cancer.

^c^Hepatocellular carcinoma.

^d^Acute promyelocytic leukemia.

^e^T cell acute lymphoblastic leukemia.

^f^Lung adenocarcinoma.

^g^Multidrug-resistant small cell lung carcinoma.

^h^Cholangiocarcinoma derived from Thai patient.

^i^Cervical carcinoma.

^j^Normal embryonic lung fibroblast; *DOX* doxorubicin, *ETO* etoposide, *I* Inactive at > 120 μM, – not determined.

The results showed that the cytotoxic activity of almost all compounds in this series was higher than that of the parent compound **ACP**. Several derivatives demonstrated a selective cytotoxic activity towards the leukemia cell lines, HL-60 and MOLT-3, whereby compound **4p** exhibited the most potent activity with IC_50_ of 6.81 ± 1.99 µM for HL-60 and 3.72 ± 0.26 µM for MOLT-3. Additionally, a derivative compound **4l** exhibited notable cytotoxic activity against the TNBC cell line MDA-MB-231, with an IC_50_ of 13.73 ± 0.04 µM.

TNBC is the most aggressive type of human breast cancer, and there are currently no effective treatments^5^. From the screening, among the tested compounds, compound **4l** has demonstrated the most promising cytotoxicity and the highest selectivity index toward MDA-MB-231 cells (Table 1, Supplementary Table S1). Moreover, this compound showed a superior selectivity index (SI = 2.9) compared to the positive drug doxorubicin (SI = 0.3), (see Supplementary Table S1). Given this potential, compound **4l** was chosen for further investigation of its cytotoxic mechanism in MDA-MB-231 cells in order to develop new drugs for TNBC treatment.

Our cytotoxic screening includes multiple cancer cell lines derived from different organ origins (breast, liver, bile duct, lung, cervical, and lymphocytes), while only one normal cell line (MRC-5 lung fibroblast) was used as a representative of normal cells. MRC-5 cell line is a well-known normal cell line that has been widely used in cytotoxic screening^31,32^. This cell line can be easily cultured in standard medium without the use of any special supplements. Fibroblasts are a common cell type present in connective tissue of many organs. The selectivity index (SI) evaluation based on cytotoxicity against MRC-5 cell line reflects the degree of side effect of the tested compounds against a widely distributed cell type in the body, but it does not imply to the side effect in the specific organs, which is a limitation of our study.

### Compound 4l induced non-apoptotic cell death in MDA-MB-231 cells

Cell death modality has been categorized into accidental non-regulated cell death (necrosis) and 12 types of regulated cell death by the Nomenclature Committee on Cell Death, based on morphological features, biochemical events during initiation of cell death, and signaling pathways involved^33^. Regulated cell death can be categorized into apoptotic cell death modes (apoptosis and anoikis) and non-apoptotic cell death modes, which are further divided into 2 groups based on the presence of vacuole accumulation such as autophagy, or the absence of vacuoles such as necroptosis^34^. The majority of reported cytotoxic natural products induce cancer cell death through apoptosis. Still, recent findings demonstrated that several natural products exerted their cytotoxic activity through induction of non-apoptotic cell death modes, such as resveratrol-induced autophagy and shikonin-induced necroptosis^35,36^.

**Figure 3:**
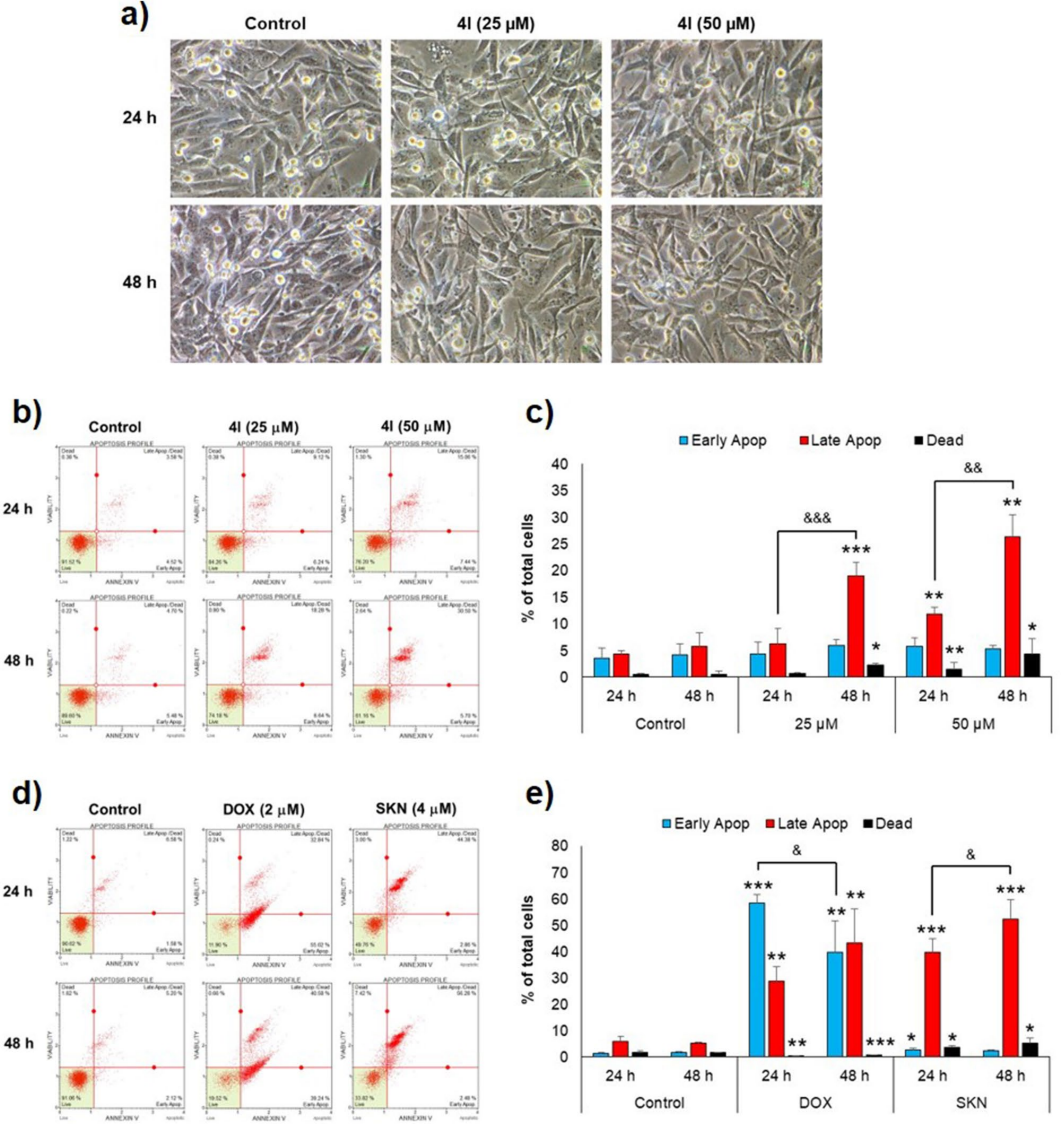
Mode of cell death in MDA-MB-231 breast cancer cells treated with compound **4l**. The cells were treated with **4l**, doxorubicin (DOX), shikonin (SKN) at indicated concentrations for 24–48 h. Then photographs were taken and the cells were subjected to annexin V/7-AAD double staining and analyzed by the flow cytometric technique. (**a**) Morphology of **4l**-treated cells, original magnification of × 400. (**b,d**) Representative dot plots of flow cytometric analysis of the treated cells showing the percentages of live cells (lower left), early apoptotic cells (lower right), late apoptotic cells (upper right), and dead cells (upper left). (**c,e**) Bar graphs displaying percentages of early apoptotic cells, late apoptotic cells, and dead cells in a total cell population. Data are expressed as mean ± SD from three independent experiments. Significant difference between treatment vs control at corresponding time points are shown by *p < 0.05, **p < 0.01, and ***p < 0.001, and significant difference between 24 h vs 48 h are indicated by ^&^*p* < 0.05, ^&&^*p* < 0.01, and ^&&&^*p* < 0.001.

Mode of cell death induced by compound **4l** was explored using MDA-MB-231 cells by observing change in cell morphology and flow cytometric analysis of annexin V/7-AAD double stained cells. The cells were treated with **4l** for 24–48 h, at concentrations of 25–50 µM, which are higher than the IC_50_ value in this cell line. Microscopically, the **4l** treatment did not result in the immediate formation of bubbles on cell surface (Fig. 3a). Accidental cell death necrosis is typically indicated by the immediate production of bubbles on the cell surface after treatment^37^. However, this morphological change was not observed in the **4l**-treated cells, indicating that **4l** treatment did not induce necrosis. Additionally, vacuole accumulation was not observed in the treated cells until 48 h after treatment (Fig. 3a). Taken together, we hypothesized that **4l**-induced cell death in MDA-MB-231 cells was not processed through necrosis or vacuole-presenting regulated cell death such as autophagy.

Interestingly, flow cytometric analysis with annexin V/7-AAD double staining showed that **4l** treatment markedly increased the late apoptotic cell population in MDA-MB-231 cells, in a dose- and time-dependent manner. In comparison, the percentage of early apoptotic cell population did not change significantly with time (Fig. 3b,c). At 48 h, the late apoptotic cell population increased significantly from 5.8% for the control group to 19.0–26.3% for **4l** at 25–50 µM (Fig. 3c). In contrast, the early apoptotic cell populations of **4l** treatments ranged from 5.3 to 5.9%, which was not statistically different from the 4.2% found in the control group (Fig. 3c), indicating that the process of **4l**-induced cell death did not progress through early stage apoptosis.

To compare annexin V/7-AAD double staining profiles of apoptotic and non-apoptotic cell death modes, doxorubicin (DOX) and shikonin (SKN) were employed as inducers of apoptosis and necroptosis, respectively. As shown in Fig. 3d,e, in DOX-induced cell death, the early apoptotic cell population was greater than the late apoptotic cell population at 24 h. Then it decreased at 48 h, concomitant with a time-dependent increase in the late apoptotic cell population (Fig. 3e). SKN, on the other hand, showed a time-dependent increase in the late apoptotic cell population during 24–48 h treatment, whereas the early apoptotic cell population was not elevated throughout the time course (Fig. 3e). The annexin V/7-AAD double staining profile of **4l**-treated cells differs from that of the apoptosis inducer and resembles that of the necroptosis inducer.

Taken together, the results suggested that **4l**-induced cell death in MDA-MB-231 cells was mediated by non-apoptotic regulated cell death, as indicated by a time-dependent increase in late apoptotic population without an increase in the early apoptotic population. Furthermore, the morphology of **4l**-treated cells did not display an accumulation of autophagic vacuoles, excluding the possibility of autophagic cell death. Necroptosis, ferroptosis, mitoptosis, parthanatos, NETosis, and pyroptosis are non-apoptotic cell death modes that do not result in vacuole accumulation^32^. The precise mode of cell death induced by compound **4l**, however, will be investigated further using multiple biochemical analyses.

Most chemotherapeutic drugs currently in use get rid of tumors by inducing apoptosis in cancer cells. However, apoptosis tolerance caused by dysregulation of apoptotic machinery is a mechanism that contributes to the development of cancer multidrug resistance, which is a major cause of failure in chemotherapeutic treatment^38^. Induction of non-apoptotic cell death mode by small molecules is an attractive strategy for overcoming the problem of apoptosis evasion^39^. Therefore, the ability of compound **4l** to induce non-apoptotic cell death is of interest, suggesting that the compound has promise for future anticancer drug development.

### Inhibitory effect of compound 4l on cell survival signaling pathways

**Figure 4:**
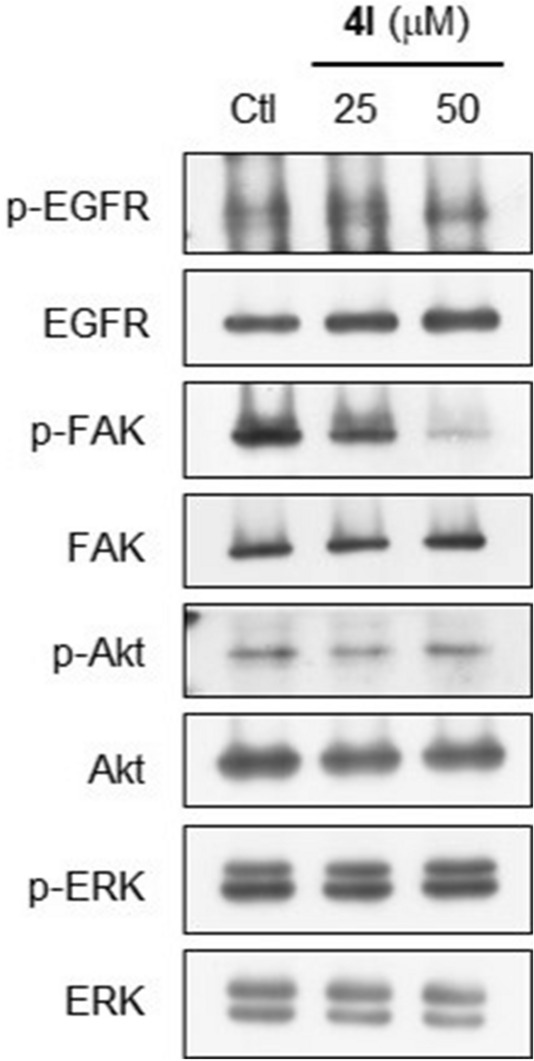
Inhibitory effect of compound **4l** on cell survival signaling pathways in MDA-MB-231 cells. After 24 h treatment with compound **4l**, phosphorylation levels of selected signaling proteins in the treated cells was detected by western blot analysis. Results are representative blots of three independent experiments.

We further investigated targets of compound **4l** on cell survival signaling pathways including EGFR, FAK, Akt, and ERK in MDA-MB-231 cells. After treatment with **4l** at concentrations of 25–50 µM for 24 h, activation (phosphorylation) of the signaling pathways was determined by using western blot analysis. As shown in Fig. 4, phosphorylation of FAK protein was selectively reduced by **4l** in a dose-dependent manner, while phosphorylation of EGFR, Akt, and ERK was not affected by the treatment, suggesting that FAK inhibition might be the cytotoxic mechanism of compound **4l**.

### FAK specific inhibitor induced non-apoptotic cell death in MDA-MB-231 cells

**Figure 5:**
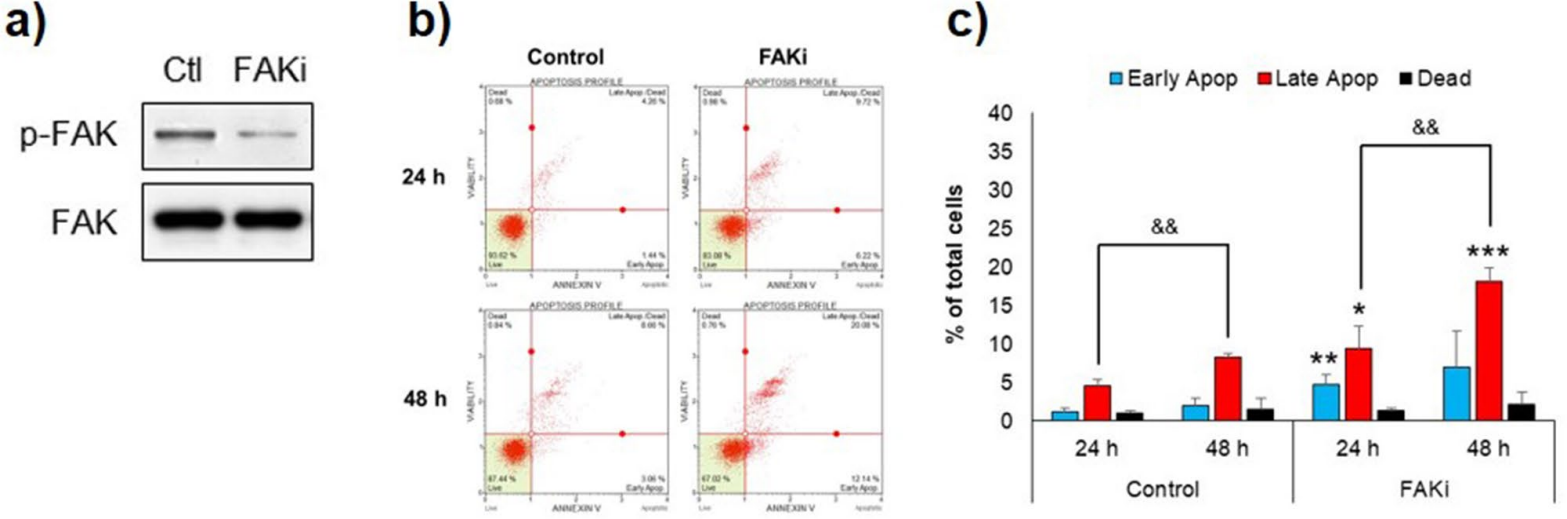
Mode of cell death in MDA-MB-231 cells exposed to FAK specific inhibitor (FAKi). The cells were treated with FAKi (16 μM) for 24–48 h. (**a**) Western blot analysis of FAK phosphorylation at 24 h after treatment. (**b**) Representative dot plots of flow cytometric analysis of the treated cells. (**c**) Bar graphs displaying percentages of early apoptotic cells, late apoptotic cells, and dead cells in total cell population. Data are expressed as mean ± SD from three independent experiments. Significant difference between treatment vs control at corresponding time point are shown by *p < 0.05, **p < 0.01, and ***p < 0.001, and significant difference between 24 vs 48 h are indicated by ^&&^*p* < 0.01.

To determine whether FAK inhibition is a mechanism involved in the induction of non-apoptotic cell death by compound **4l** in MDA-MB-231 cells, the cells were treated with FAK specific inhibitor (1,2,4,5-benzenetetraamine or FAKi) at a cytotoxic concentration (16 μM), followed by analysis of the mode of cell death. As shown in Fig. 5a, after 24 h treatment, FAK phosphorylation was clearly inhibited in FAKi-treated cells. Throughout the 48-h treatment period, a time-dependent increase in the late apoptotic cell population was observed in FAKi-treated cells, which was always greater than the early apoptotic cell population (Fig. 5b,c). The results indicated that FAK inhibition in MDA-MB-231 cells caused non-apoptotic cell death, similar to that observed in **4l**-treated cells.

Several lines of evidence indicate that FAK regulates cell survival as well as several malignant characteristics of cancer cells and is frequently overexpressed in a wide range of tumors^40^. FAK signaling has been reported to be involved in the radio/chemo resistance of cancers. Downregulation of FAK increases the cytotoxic effect of radiation in colon cancer cells^41^, and also enhances cisplatin sensitivity in TNBC cells^42^. In vivo evaluation of a small molecule FAK inhibitor BI 856520 in mouse models of breast cancer yields promising results on suppression of primary tumor growth and outgrowth of metastatic tumors, through impairing cell proliferation both in vitro and in vivo^43^. Several FAK inhibitors are currently being tested in clinical trials in various cancer types, either as single therapy or in combination with other anticancer drugs^44^. Therefore, the compound **4l** might be a potential compound for development as a chemotherapeutic agent to treat TNBC.

### Identification of a potential binding site of compound 4l in FAK kinase domain

**Figure 6:**
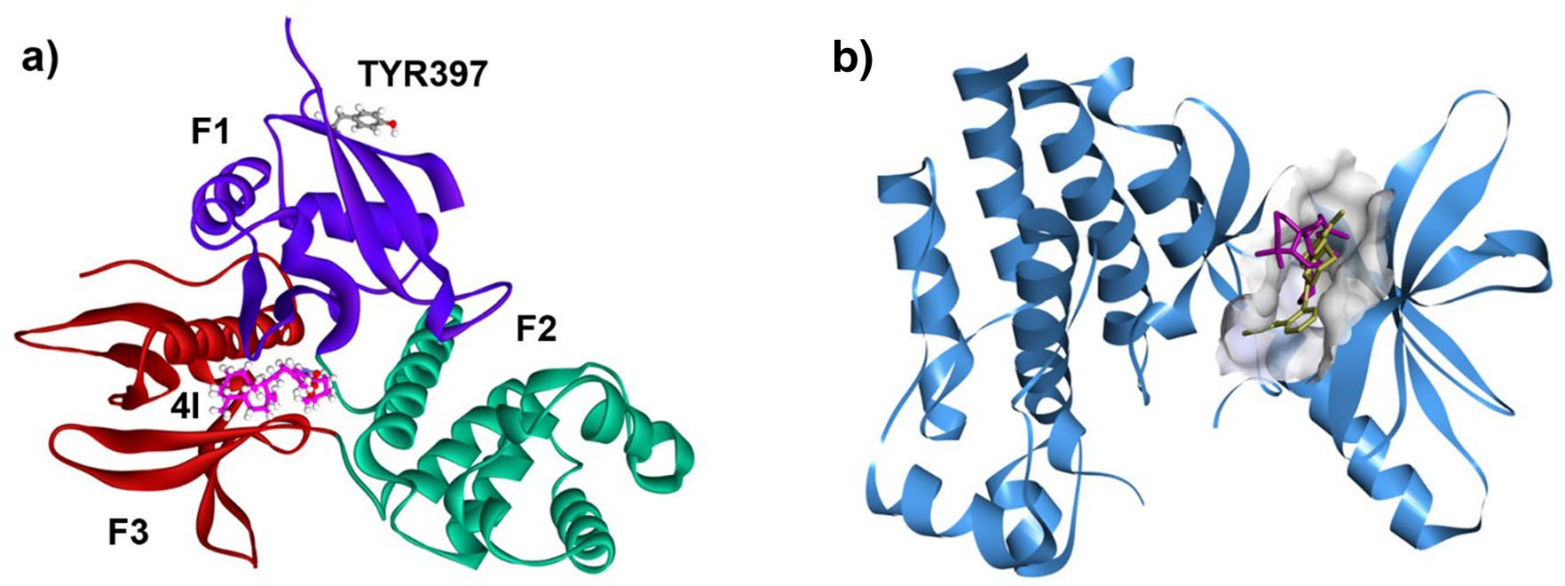
The binding positions of **4l** on FAK protein. (**a**) FAK FERM domain (PDB:2AL6), the phosphorylation site Tyr397 are indicated, three subdomains are labeled with colors violet (F1), green (F2), and red (F3), and compound **4l** is shown in pink stick. (**b**) FAK kinase domain (PDB ID: 2JKK) with compound **4l** (pink stick) and TAE226 (yellow stick) in the ATP binding pocket (grey).

To explore the binding between FAK and compound **4l**, the molecular docking was conducted using iGEMDOCK v2.1 software. Two possible binding sites for small molecule inhibitor on FAK protein, FERM domain and kinase domain, were used as receptor. Unlike FAKi which has been shown to bind to the FERM domain at a pocket closed to Tyr397^45^, the binding site of **4l** on the FERM domain appears to be far from the Tyr397 pocket (Fig. 6a). On the other hand, the binding site of **4l** on the kinase domain was located in an ATP binding pocket (Fig. 6b), which was identical to the binding site of another FAK specific inhibitor, TAE226^46^, indicating that FAK catalytic activity would be inhibited upon binding of **4l**. These results suggested that compound **4l** reduced Tyr397 autophosphorylation of FAK by interfering with FAK kinase activity via binding to the kinase domain rather than the FERM domain.

**Figure 7:**
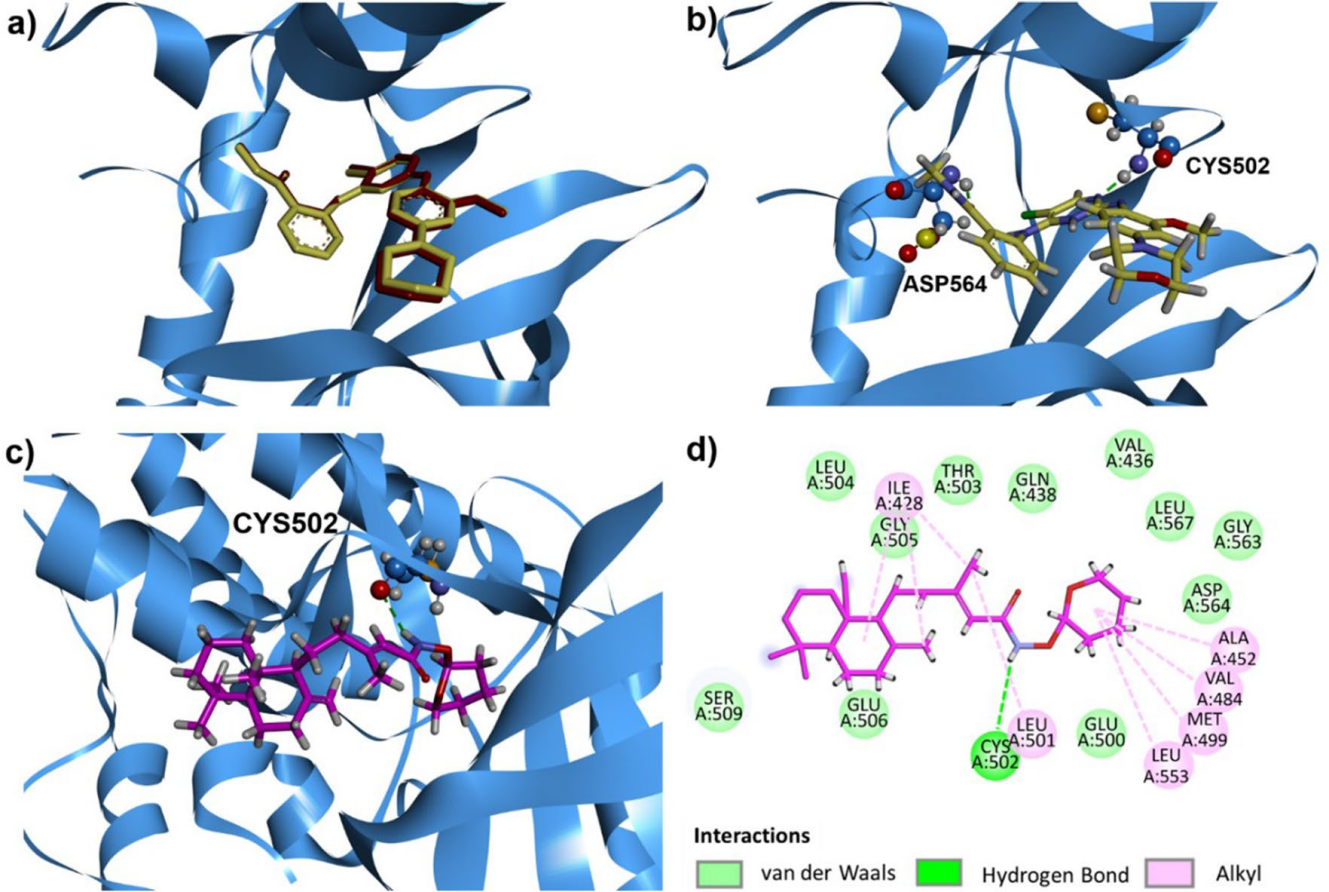
Superposition of redocked TAE226 (red) and co-crystallized TAE226 (yellow) (**a**), hydrogen bond interaction of TAE226 (**b**), hydrogen bond interaction of **4l** (pink) (**c**) and 2D diagram representing interactions of **4l** (**d**) in the ATP binding pocket of FAK kinase domain (PDB ID: 2JKK).

As illustrated in Fig. 7a, redock TAE226 was located to the same position as co-crystallized TAE226, indicating that our docking method was acceptable. The binding energy of TAE226 was − 134.05 kcal/mol, and TAE226 formed two hydrogen bonds with Asp564 and Cys502 (Fig. 7b). Moreover, as shown in Table 2, **4l** had the binding energy of − 80.44 kcal/mol. Figure 7c demonstrates that **4l** formed one hydrogen bond with Cys502 of FAK kinase domain. Fused-cyclohexane rings, methyl carbon and methylene carbon of **4l** also made hydrophobic contacts with Ile428 and Leu501 (Fig. 7d). It can also be noticed that there were hydrophobic interactions between the tetrahydropyran ring of **4l** and four amino acid side chains including Ala452, Val484, Met499, and Leu553 (Fig. 7d). This indicates that fused-cyclohexane rings and tetrahydropyran ring of **4l** can act as the key moieties in binding to the catalytic site of FAK, making **4l** a potential FAK inhbitor.

**Table 2:** Summary of binding energies, amino acid interaction, and hydrogen bond length of **4l** and **TAE226** in the ATP binding pocket of FAK kinase domain.

Compounds	Binding energy (kcal/mol)	Amino acid residues	H-bond length (Å)
**4l**	− 80.4351	CYS502	2.90
**TAE226**	− 134.047	ASP564, CYS502	2.06, 1.76

### The predicted physicochemical properties of 4l

To evaluate the druggability^47,48^ of compound **4l**, the SwissADME website service was used to study the physicochemical properties and analysis by Lipinski’s and Veber’s rules. The calculation of the parent compound **ACP** was also performed to compare with **4l**. The calculated parameters are presented in Table 3. Considering the Lipinski’s rule of five, the parameters of these two compounds, including the molecular weight, the number of hydrogen bond acceptors and the number of hydrogen bond donors abided by the rule. However, the MLogP values, representing the lipophilicity of the compound, are slightly greater than the reported requirement (4.15)^49^. These MLogP values are consistent with the LogS values, which indicated the lower water solubility of these compounds. Nevertheless, compound **4l** and **ACP** have high degree of fraction sp3 (Fsp3), indicating high degree of saturation and chirality content, which could promote a significant preference for binding and selectivity to proteins. Lastly, compound **4l** and **ACP** have TPSA values much less than 140 Å^2^ indicating good cell membrane permeability as molecules with a TPSA value more than 140 (Å^2^) tend to be poor at permeating cell membranes^50^. Moreover, the introduction of the amide substituents leads to an increase in the TPSA of **4l**, making the TSPA value of **4l** close to the range that attributed to most successful drug (≤ 60–70 Å^2^). According to the physiochemical parameters, both **4l** and **ACP** exhibited parameters suitable for appropriate drug ability, except for the solubility of the compounds. The chemical modification of the parent compounds in drug discovery generally aims to improve either physiochemical properties or potency of the drugs, or both. In this study, eventhough compound **4l** did not show improvement in terms of the physiochemical parameters compared to the parent compound **ACP**, compound **4l** exhibited much higher potency than **ACP**, indicating improvement in the potency of the drugs from the modification. Further derivatization of the compounds could allow for better physiochemical values that conform to the drug likeness guideline.

**Table 3:** The physicochemical properties of active compound **4l** compared with **ACP**.

Compounds	MW	Fraction Csp3	TPSA	MLogP	LogS	nHBA	nHBD	nRB
**4l**	391.59	0.88	47.56	4.58	− 6.52	3	1	7
**ACP**	292.46	0.84	37.30	4.41	− 5.69	2	1	4

*MW* molecular weight, *Fraction Csp3* the ratio of *sp*^3^ hybridized carbons over the total carbon count of the molecule, *TPSA* topological polar surface area [Å^2^], *MLogP* lipophilicity parameter: Moriguchi octanol–water partition coefficient (MLogP) is based on quantitative structure-logP relationships by using topological indexes, *LogS* water solubility parameter, LogS scale insoluble <  − 10 < poorly <  − 6 < moderately <  − 4 < soluble <  − 2 very < 0 < highly, *nHBA* number of hydrogen bond acceptors, *nHBD* number of hydrogen bond donors, *nRB* number of rotatable bonds.

## Methods

### General experiment

^1^H- and ^13^C-NMR spectra were recorded in CDCl_3_ or DMSO-*d*_6_ using a Bruker AVANCE 300 NMR or a Bruker AVANCE 400 NMR spectrometer. ^1^H-NMR and ^13^C-NMR chemical shifts (*δ*) Chemical shifts were expressed in ppm and referenced to the residual solvent signals. Coupling constants (*J*) were reported in Hertz (Hz). IR spectra were recorded on a PerkinElmer Spectrum One Spectrometer using a universal attenuated reflectance (ATR) technique and are reported in cm^-1^. HRESIMS analysis was determined using a Bruker Daltonics microTOF spectrometer. Optical rotations were measured on a JASCO P-1020 polarimeter. All glassware was heat-dried prior to use. TLC were visualized using UV light (254 and 366 nm) and Godin’s reagent.

### Plant material

The rhizomes of wild-type *K. elegans* used in this study were from the rural area in Sai Yok District, Kanchanaburi Province, Thailand and were collected by the local people who live in the area under their permission. The plant was authenticated by a taxonomist, Professor Dr. Wongsatit Chuakul of the Faculty of Pharmacy, Mahidol University, Thailand. The voucher specimen number BKF 192,348 (Thongnest No. 2) was deposited at the Department of National Parks, Wildlife and Plant Conservation, Ministry of Natural Resources and Environment, Bangkok, Thailand. All procedures for plant collection were performed in accordance with relevant institutional, national, and international guidelines and legislation.

### Chemicals and antibodies

All solvents were distilled from commercial grade solvents except where indicated otherwise. Dichlromethane (CH_2_Cl_2_) was further purified by pressure filtration through activated alumina for reaction set up. Spectral grade solvents were used for spectroscopic measurements. Thin layer chromatography was performed on Merck precoated silica gel 60 F254 plates. Silica gel 60 (Silicycle, 230*–*400 mesh) was used for flash column chromatography, and Silica gel 60 PF254 (Merck) was used for preparative thin layer chromatography. Silica gel precoated aluminum plates (F254, 0.25 mm) were used for TLC detection. Muse® Annexin V & Dead Cell Kit was purchased from Luminex (Austin, TX, USA). Doxorubicin, Etoposide, and shikonin were obtained from Sigma-Aldrich (St. Louis, MO, USA). FAK specific inhibitor (1,2,4,5-benzenetetraamine tetrahydrochloride or FAK inhibitor 14) was purchased from Tocris Bioscience (Bristol, U.K.). Protease/phosphatase inhibitor cocktail and all antibodies were obtained from Cell Signaling Technology (Beverly, MA, USA). SuperSignal™ ECL substrates were purchased from Thermo Scientific (Rockford, IL, USA).

### Cell lines

MDA-MB-231 (triple-negative breast cancer), T-47D (hormone-dependent breast cancer), HepG2 (hepatocellular carcinoma), HL-60 (acute promyelocytic leukemia), MOLT-3 (T-cell acute lymphoblastic leukemia), A549 (lung adenocarcinoma), H69AR (multidrug-resistant small cell lung carcinoma), HeLa (cervical carcinoma), and MRC-5 (normal embryonic lung fibroblast) were obtained from the American Type Culture Collection (ATCC, Manassas, VA, USA). HuCCA-1 (cholangiocarcinoma derived from a Thai patient) was obtained from Laboratory of Immunology, Chulabhorn Research Institute, Thailand.

### Isolation of anticopalic acid from the *K. elegans*

The rhizomes of *K. elegans* (25 kg) were extracted with dichloromethane (2 × 70 L) at room temperature. After evaporating the solvent, a crude extract (420.6 g) was obtained. The crude dichloromethane extract (420.6 g) was chromatographed over silica gel column, using a step gradient system with hexane-CH_2_Cl_2_ (100:0 to 0:100) and CH_2_Cl_2_-MeOH (100:0 to 0:100) to yield eighteen fractions (F1-F18) after TLC detection. One of the **ACP** rich fractions (Fraction F7, 38.5 g) was purified by silica column chromatography eluted with n-hexane-CH_2_Cl_2_ (95:5 to 0:100) and CH_2_Cl_2_-MeOH (100:0 to 90:10) to give pure **ACP** as white amorphous (34.5 g). The structure of **ACP** obtained from the plant extract was identified based on ^1^H and ^13^C NMR spectral data compared with the previous data^16^. Several other **ACP**-rich fractions will be used for future isolation in due course.

### Preparation of anticopalic acid derivatives

#### The synthesis of anticopalol (2)

To a solution of ACP (25 mg, 0.08 mmol, 1.0 equiv.) in CH_2_Cl_2_ (0.8 mL, 0.1 M) was added HOBt (16.5 mg, 0.12 mmol, 1.5 equiv) and EDCl (23.4 mg, 0.12 mmol, 1.5 equiv.). The mixture was stirred at room temperature for 30 min and then concentrated in vacuo. The residue was dissolved in THF (0.8 mL, 0.1 M) and cooled to 0 °C, and NaBH_4_ (9.2 mg, 0.24 mmol, 3.0 equiv.) was added into the mixture. Then, H_2_O (2 µL, 0.12 mmol, 1.5 equiv.) was added into a solution. The resulting mixture was stirred at 0 °C for 30 min, and then quenched with MeOH (1 mL). The mixture was dried under vacuum and redissolved in EtOAc. The organic layer was wash with 10% citric acid, brine, and dried over Na_2_SO_4_, and concentrated under vacuum. The product was purified by column chromatography eluting with EtOAc:hexane (10:90) to provide 6 mg (25%) of anticopalol (**2**)^16^ as a colorless oil. ${[\mathrm{\alpha }]}_{\mathrm{D}}^{27}$ =  + 30.3 (*c* 0.95, CHCl_3_); FTIR (neat) *Ѵ*_max_: 3356, 2923, 2847, 1662, 1642, 1457, 1442, 1387, 996, 887 cm^‒1^; ^1^H NMR (600 MHz, CDCl_3_) *δ*_H_ 4.83 (1H, d, *J* = 1.3 Hz, H-17), 4.51 (1H, d, *J* = 1.0 Hz, H-17), 4.18 (1H, dd, *J* = 6.9, 1.2 Hz, H-14), 4.18 (1H, d, *J* = 6.9 Hz, H-15), 2.40 (1H, ddd, *J* = 12.8, 4.2, 2.5 Hz, H-7), 2.16 (2H, ddd, *J* = 14.0, 9.8, 4.0 Hz, H_2_-12), 1.98 (1H, td, *J* = 12.9, 5.0 Hz, H-7), 1.76 (1H, m, H-1), 1.71 (1H, m, H-6), 1.67 (3H, s, H_3_-16), 1.61 (1H, m, H-11), 1.57 (1H, m, H-9), 1.56 (1H, m, H-2), 1.49 (1H, m, H-2), 1.43 (1H, m, H-11), 1.39 (1H, m, H-3), 1.32 (1H, tdd, *J* = 12.9, 12.9, 4.3 Hz, H-6), 1.18 (1H, td, *J* = 12.8, 4.9 Hz, H-3), 1.10 (1H, dd, *J* = 12.5, 2.7 Hz, H-5), 0.90 (3H, s, H_3_-19), 0.80 (3H, s, H_3_-18), 0.67 (3H, s, H_3_-20); ^13^C NMR (150 MHz, CDCl_3_) *δ* 148.6 (C-8, s), 140.6 (C-13, s), 123.1 (C-14, d), 106.2 (C-17, t), 59.4 (C-15, t), 56.4 (C-9, d), 55.6 (C-5, d), 42.2 (C-3, t), 39.7 (C-10, s), 39.1 (C-1, t), 38.5 (C-12, t), 38.4 (C-7, t), 33.7 (C-19, q), 33.6 (C-4, s), 24.5 (C-6, t), 21.9 (C-11, t), 21.7 (C-19, q), 19.4 (C-2, t), 19.3 (C-16, q), 14.5 (C-20, q); ESI-TOF MS: calcd for C_20_H_34_NaO, *m/z* 313.2502 [M + Na]^+^ found 340.2502.

#### The synthesis of (E)-3-methyl-5-((1S,8aS)-5,5,8a-trimethyl-2-methylenedecahydronaphthalen-1-yl)pent-2-enal (3)

Anticopalol (**2**) (5.5 mg, 0.018 mmol, 1.0 equiv.) was dissolved in CH_2_Cl_2_ (0.18 mL, 0.1 M). Then, Dess-Martin periodinane (9.6 mg, 0.022 mmol, 1.2 equiv.) was slowly added into a mixture. The solution was kept stirring at room temperature for 3 h. The reaction was worked up with NH_4_Cl (aq.). The mixture was extracted with EtOAc, brine, and dried over Na_2_SO_4_.The crude extract was concentrated under vacuum and purified by column chromatography eluting with EtOAc:hexane (20:80) to give 6.6 mg (100%) of the aldehyde **3** as a yellow oil. ${[\mathrm{\alpha }]}_{\mathrm{D}}^{27}$ =  + 10.5 3 (*c* 0.48, CHCl_3_); FTIR (neat) *Ѵ*_max_: 2925, 2865, 1694, 1642, 1459, 1441, 1387, 1260, 1094, 1018, 887, 802 cm^‒1^; ^1^H NMR (400 MHz, CDCl_3_) *δ*_H_ 9.99 (1H, d, *J* = 8.0 Hz, H-15), 5.87 (1H, d, *J* = 8.0 Hz, H-14), 4.84 (1H, brs, H-17), 4.46 (1H, brs, H-17), 2.39 (1H, m, H-7), 2.32 (1H, m, H-12), 2.15 (3H, s, H_3_-16), 2.04 (1H, m, H-12), 1.94 (1H, m, H-7), 1.74 (1H, m, H-11), 1.74 (1H, m, H-6), 1.65 (1H, m, H-1), 1.62 (1H, m, H-9), 1.50 (1H, m, H-11), 1.45 (2H, m, H_2_-2), 1.33 (1H, m, H-3), 1.25 (1H, m, H-6), 1.16 (1H, m, H-3), 1.10 (1H, m, H-5), 0.85 (3H, s, H_3_-19), 0.78 (3H, s, H_3_-18), 0.68 (3H, s, H_3_-20)); ^13^C NMR (100 MHz, CDCl_3_) *δ*_C_ 191.3 (C-15, d), 164.9 (C-13, s), 148.1 (C-8, s), 127.1 (C-14, d), 106.3 (C-17, t), 56.1 (C-9, d), 55.5 (C-5, d), 42.0 (C-3, t), 39.7 (C-12, t), 39.5 (C-10, s), 39.0 (C-1, t), 38.2 (C-7, t), 33.5 (C-4, s), 33.5 (C-19, q), 24.4 (C-6, t), 21.7 (C-18, q), 21.2 (C-11, t), 19.3 (C-2, t), 17.6 (C-16, q), 14.4 (C-20, q); ESI MS: calcd for C_20_H_32_NaO, *m/z* 311.2345 [M + Na]^+^ found 311.2352.

#### General procedure for preparation of the amide derivatives of ACP 4a–4r

A mixture of ACP (25 mg, 1.0 equiv.), 1-hydroxybenzotriazole hydrate (HOBt) (1.5 equiv.), *N*-(3-dimethylaminopropyl)-*N*′-ethylcarbodiimide hydrochloride (EDCI) (1.5 equiv.), amine derivative (1.5 equiv.) in CH_2_Cl_2_ (0.1 M) was stirred at 0 °C for 5 min, and then *N*,*N*-diisopropylethylamine (DIPEA) (3 equiv.) was slowly added into the mixture. The reaction mixture was stirred at 0 °C for additional 15 min and further stirred at room temperature for 24 h. Then, the reaction was quenched with aqueous 1 N hydrochloric acid solution and extracted with EtOAc (× 3). The organic layers were combined, washed with brine, and dried with MgSO_4_ (s) to obtain a crude fraction, which was further purified by flash silica gel column chromatography, eluted with either EtOAc: hexane or CH_2_Cl_2_:hexane to provide the amide derivatives of ACP **4a**–**4r**.

#### (E)-N,3-Dimethyl-5-((1S,4aS,8aS)-5,5,8a-trimethyl-2-methylenedecahydro-naphthalen-1-yl)pent-2-enamide (4a)

The crude residue was pre-absorbed on silica gel and purified by flash silica gel column chromatography using a mixture of CH_2_Cl_2_:MeOH (99:1) as an eluent to provide **4a** (17.2 mg, 67%) as yellow oil. ${[\mathrm{\alpha }]}_{D}^{25}$ +44.0 (*c* 1.69 CHCl_3_); FTIR (neat) *Ѵ*_max_: 3298, 3078, 2927, 2866, 2843, 1660, 1630, 1548, 1458, 1442, 1410, 1387, 1365, 1262, 1181, 886, 863, 737, 673 cm^‒1^; ^1^H NMR (400 MHz, CDCl_3_) *δ*_H_ 5.49 (1H, br s, H-14), 4.81 (1H, br s, H_2_-17), 4.47 (1H, br s, H_2_-17), 2.81 (3H, d, *J* = 5.0 Hz, H_3_-1ʹ), 2.36 (1H, ddd, *J* = 13.0, 4.0, 2.0 Hz, H_2_-7), 2.21 (1H, ddd, *J* = 14.0, 9.0, 4.0 Hz, H_2_-12), 2.12 (3H, d, *J* = 2.0 Hz, H_3_-16), 1.96 (1H, m, H_2_-7), 1.87 (1H, m, H-12), 1.73 (1H, m, H_2_-1), 1.73 (1H, m, H_2_-6), 1.65 (1H, m, H_2_-11), 1.60 (1H, m, H_2_-2), 1.57 (1H, m, H-9), 1.53 (1H, m, H_2_-2), 1.47 (1H, m, H_2_-11), 1.43 (1H, m, H_2_-3), 1.30 (1H, dd, *J* = 13.0, 4.0 Hz, H_2_-6), 1.17 (1H, td, *J* = 13.0, 4.0 Hz, H_2_-3), 1.05 (1H, dd, *J* = 13.0, 3.0 Hz, H-5), 0.96 (1H, td, *J* = 13.0, 4.0 Hz, H_2_-1), 0.85 (3H, s, H_3_-18), 0.78 (3H, s, H_3_-19), 0.66 (3H, s, H_3_-20); ^13^C NMR (100 MHz, CDCl_3_) *δ*_C_ 168.0 (C-15, s), 154.8 (C-13, s), 148.5 (C-8, s), 117.6 (C-14, d), 106.3 (C-17, t), 56.1 (C-9, d), 55.5 (C-5, d), 42.1 (C-3, t), 39.7 (C-10, s), 39.5 (C-12, t), 39.0 (C-1, t), 38.3 (C-7, t), 33.6 (C-18, q), 33.6 (C-4, s), 26.0 (C-1ʹ, q), 24.4 (C-6, t), 21.7 (C-19, q), 21.5 (C-11, t), 19.4 (C-2, t), 18.2 (C-16, q), 14.5 (C-20, q); ESI-TOF MS: calcd for C_21_H_35_NNaO, *m/z* 340.2610 [M + Na]^+^ found 340.2610.

#### (E)-N-Allyl-3-methyl-5-((1S,4aS,8aS)-5,5,8a-trimethyl-2-methylenedeca-hydronaphthalen-1-yl)pent-2-enamide (4b)

The crude residue was pre-absorbed on silica gel and purified by flash silica gel column chromatography using a mixture of EtOAc:hexane (5:95) as an eluent to provide **4b** (21.4 mg, 78%) as a yellow oil. ${[\mathrm{\alpha }]}_{D}^{25}$ +38.3 (c 1.98, CHCl_3_); FTIR (neat) *Ѵ*_max_: 3293, 3078, 2926, 2866, 2844, 1660, 1630, 1536, 1459, 1442, 1388, 1365, 1256, 1178, 988, 915, 887 cm^‒1^. ^1^H NMR (400 MHz, CDCl_3_) *δ* 5.86 (1H, m, H-2ʹ), 5.53 (1H, dd, *J* = 2.4, 1.2 Hz, H-14), 5.20 (1H, m, H_2_-3ʹ), 5.13 (1H, m, H_2_-3ʹ), 4.83 (1H, d, *J* = 1.6 Hz, H_2_-17), 4.49 (1H, d , *J* = 1.2 Hz, H_2_-17), 3.92 (2H, tt, *J* = 6.0, 1.6 Hz., H_2_-1ʹ), 2.3389 (1H, ddd, *J* = 13.0, 4.4, 2.4 Hz, H_2_-7), 2.24 (1H, ddd, *J* = 12.0, 10.0, 4.4 Hz, H_2_-12), 2.16 (3H, d, *J* = 1.2 Hz, H_3_-16), 1.97 (1H, m, H_2_-7), 1.91 (1H, m, H_2_-12), 1.75 (1H, m, H_2_-1), 1.72 (1H, m, H_2_-6), 1.67 (1H, m, H_2_-11), 1.58 (1H, m, H_2_-2), 1.55 (1H, m, H-9), 1.50 (1H, m, H_2_-2), 1.46 (1H, m, H_2_-11), 1.39 (1H, m, H_2_-3), 1.32 (1H, dd, *J* = 13.0, 4.4 Hz, H_2_-6), 1.17 (1H, td, *J* = 13.4, 4.2 Hz, H_2_-3), 1.08 (1H, dd, *J* = 12.0, 2.7 Hz, H-5), 1.01 (1H, td, *J* = 13.0, 4.0 Hz, H_2_-1), 0.87 (3H, s, H_3_-18), 0.80 (3H, s, H_3_-19), 0.68 (3H,s, H_3_-20). ^13^C NMR (100 MHz, CDCl_3_) *δ* 166.9 (C-15, s), 155.6 (C-13, s), 148.5 (C-8, s) 134.6 (C-2ʹ, d), 117.5 (C-14, d), 116.3 (C-3ʹ, t), 106.3 (C-17, t), 56.1 (C-9, d), 55.5 (C-5, d), 42.1 (C-1ʹ, t), 41.6 (C-3, t), 39.7 (C-12, t), 39.6 (C-10, s), 39.0 (C-1, t), 38.3 (C-7, t), 33.6 (C-4, s), 33.6 (C-18, q), 24.5 (C-6, t), 21.7 (C-19, q), 21.5 (C-11, t), 19.4 (C-2, t), 18.4 (C-16, q), 14.5 (C-20, q)); ESI-TOF MS: calcd for C_23_H_37_NNaO, *m/z* 366.2767 [M + Na]+, found 366.2766.

#### (E)-3-Methyl-N-(prop-2-yn-1-yl)-5-((1S,4aS,8aS)-5,5,8a-trimethyl-2-methyl enedecahydronaphthalen-1-yl)pent-2-enamide (4c)

The crude residue was pre-absorbed on silica gel and purified by flash silica gel column chromatography using a mixture of EtOAc:hexane (5:95) as an eluent to provide **4c** (21.1 mg, quantitative yield) as a yellow oil. ${[\mathrm{\alpha }]}_{D}^{25}$ +32.9 (*c* 1.69, CHCl_3_); FTIR (neat) *Ѵ*_max_: 3309, 2927, 2847, 1662, 1637, 1527, 1459, 1442, 1387, 1366, 1253, 1176, 1115, 886 cm^‒1^. ^1^H NMR (400 MHz, CDCl_3_) *δ* 5.59 (1H, br s, NH), 5.51 (1H, br d, *J* = 0.8 Hz, H-14), 4.83 (1H, d, *J* = 1.6 Hz, H_2_-17), 4.48 (1H, d, *J* = 1.2 Hz, H_2_-17), 4.10 (2H, dd, *J* = 5.6, 2.8 Hz, H_2_-1ʹ), 2.38 (1H, ddd, *J* = 13.0, 4.4, 2.4 Hz, H_2_-7), 2.26 (1H, dd, *J* = 10.0, 4.0 Hz, H_2_-12), 2.23 (1H, t, *J* = 2.8 Hz, H-3ʹ), 2.16 (3H, d, *J* = 1.2 Hz, H_3_-16), 1.97 (1H, dd, *J* = 13.0, 5.0 Hz, H_2_-7), 1.91 (1H, m, H_2_-12), 1.75 (1H, m, H_2_-1), 1.71 (1H, m, H_2_-6), 1.64 (1H, m, H_2_-11), 1.58 (1H, m, H_2_-2), 1.55 (1H, m, H-9), 1.49 (1H, m, H_2_-2), 1.45 (1H, m, H-11), 1.39 (1H, m, H_2_-3), 1.32 (1H, dd, *J* = 13.0, 4.4 Hz, H_2_-6), 1.17 (1H, td, *J* = 13.0, 4.4 Hz, H_2_-3), 1.08 (1H, dd, *J* = 13.0, 2.8 Hz, H-5), 1.00 (1H, td, *J* = 13.0, 4.4 Hz, H_2_-1), 0.87 (3H, s, H_3_-18), 0.80 (3H, s, H_3_-19), 0.68 (3H, s, H_3_-20). ^13^C NMR (100 MHz, CDCl_3_) *δ* 166.6 (C-15, s), 156.9 (C-13, s), 148.4 (C-8, s), 116.8 (C-14, d), 106.3 (C-17, t), 79.9 (C-2ʹ, s), 71.4 (C-3ʹ, d), 56.0 (C-9, d), 55.4 (C-5, d), 42.1 (C-3, t), 39.66 (C-10, s), 39.65 (C-12, t), 39.0 (C-1, t), 38.3 (C-7, t), 33.59 (C-18, q), 33.57 (C-4, s), 28.9 (C-1ʹ, t), 24.4 (C-6, t), 21.7 (C-19, q), 21.5 (C-11, t), 19.4 (C-2, t), 18.4 (C-16, q), 14.5 (C-20, q). ESI-TOF MS: calcd for C_23_H_36_NO, *m/z* 342.2791 [M + H]^+^, found 342.2794.

#### (E)-3-Methyl-N-octyl-5-((1S,4aS,8aS)-5,5,8a-trimethyl-2-methylenedecahydronaphthalen-1-yl)pent-2-enamide (4d)

The crude residue was pre-absorbed on silica gel and purified by flash silica gel column chromatography using a mixture of EtOAc:hexane (10:90) as an eluent to provide **4d** (26.9 mg, 80% yield) as a yellow oil. ${[\mathrm{\alpha }]}_{D}^{26}$ +28.2 (c 2.51, CHCl_3_); FTIR (neat) *Ѵ*_max_: 3293, 3078, 2924, 2851, 1659, 1628, 1542, 1459, 1441, 1387, 1365, 1259, 887 cm^‒1^. ^1^H NMR (400 MHz, CDCl_3_) *δ* 5.5 (1H, d, *J* = 1.2 Hz, H-14), 5.39 (1H, br s, NH), 4.83 (1H, d, *J* = 1.5 Hz, H_2_-17), 4.49 (1H, d, *J* = 1.2 Hz, H_2_-17), 3.27 (2H, m, H_2_-1ʹ), 2.38 (1H, ddd, *J* = 12.0, 4.4, 2.4 Hz, H_2_-7), 2.23 (1H, ddd, *J* = 12.0, 10.0, 4.0 Hz, H_2_-12), 2.14 (3H, d, *J* = 1.2 Hz, H_3_-16), 1.97 (1H, m, H_2_-7), 1.90 (1H, m, H_2_-12), 1.76 (1H, m, H_2_-1), 1.72 (1H, m, H_2_-6), 1.65 (1H, m, H_2_-11), 1.58 (1H, m, H_2_-2), 1.56 (1H, m, H-9), 1.53‒1.47 (2H, m, H-2'), 1.49 (2H, m, H_2_-2/H_2_-2ʹ), 1.45 (1H, m, H_2_-11), 1.39 (1H, m, H_2_-3), 1.33 (1H, m, H_2_-6), 1.32‒1.25 (10H, m, H-3ʹ/H-4ʹ/ H-5ʹ/H-6ʹ/H-7ʹ), 1.17 (1H, td, *J* = 13.0, 4.0 Hz, H-3), 1.08 (1H, dd, *J* = 13.0, 4.0 Hz, H-5), 1.01 (1H, td, *J* = 13.0, 4.0 Hz, H-1), 0.88 (3H, m, H_3_-8ʹ), 0.87 (3H, s, H_3_-18), 0.80 (3H, s, H_3_-19), 0.68 (3H, s, H_3_-20). ^13^C NMR (100 MHz, CDCl_3_) *δ* 167.2 (C-15, s), 154.7 (C-13, s), 148.5 (C-8, s), 117.9 (C-14, d), 106.3 (C-17, t), 56.1 (C-9, d), 55.5 (C-5, d), 42.1 (C-3, t), 39.7 (C-10, s), 39.6 (C-12, t), 39.2 (C-1ʹ, t), 39.0 (C-1, t), 38.3 (C-7, t), 33.6 (C-18, q), 33.58 (C-4, s), 31.8 (C-6ʹ, t), 29.8 (C-2ʹ, t), 29.3 (C-4ʹ, t), 29.2 (C-5ʹ, t), 27.0 (C-3ʹ, t), 24.5 (C-6, t), 22.6 (C-7ʹ, t), 21.7 (C-19, q), 21.5 (C-11), 19.3 (C-2), 18.3 (C-16, q), 14.5 (C-20, q), 14.1 (C-8ʹ, q). ESI-TOF MS: calcd for C_28_H_49_NNaO, *m/z* 438.3706 [M + Na]^+^, found 438.3695.

#### (E)-3-Methyl-N-phenyl-5-((1S,4aS,8aS)-5,5,8a-trimethyl-2-methylene-decahydronaphthalen-1-yl)pent-2-enamide (4e)

The crude residue was pre-absorbed on silica gel and purified by flash silica gel column chromatography using a mixture of EtOAc:hexane (5:95) as an eluent to provide **4e** (16.7 mg, 54% yield) as a yellow oil.${[\mathrm{\alpha }]}_{D}^{25}$ +35.2 (*c* 0.69, CHCl_3_); FTIR (neat) *Ѵ*_max_: 3308, 2927, 2845, 1661, 1641, 1598, 1541, 1499, 1440, 1387, 1309, 1252, 1152, 1079, 888, 752, 691 cm^‒1^. ^1^H NMR (400 MHz, CDCl_3_) *δ* 7.55 (1H, d, *J* = 7.6 Hz, H-2ʹ), 7.32 (3H, m, H-3ʹ/H-4ʹ/H-5ʹ), 7.08 (1H, br t, *J* = 7.6 Hz, H-6ʹ), 5.66 (1H, d, *J* = 1.0 Hz, H-14), 4.82 (1H, d, *J* = 1.1 Hz, H_2_-17), 4.52 (1H, br s, H_2_-17), 2.40 (1H, ddd, *J* = 13.0, 4.4, 2.4 Hz, H_2_-7), 2.27 (1H, ddd, *J* = 13.0, 10.4, 1.2 Hz, H_2_-12), 2.22 (3H, d, *J* = 1.2 Hz, H_3_-16), 1.98 (1H, m, H_2_-7), 1.95 (1H, m, H_2_-12), 1.76 (1H, m, H_2_-1), 1.74 (1H, m, H_2_-6), 1.71 (1H, m, H_2_-11), 1.61 (1H, m, H_2_-2), 1.58 (1H, m, H-9), 1.53 (1H, m, H_2_-2), 1.48 (1H, m, H_2_-11), 1.40 (1H, m, H_2_-3), 1.33 (1H, dd, *J* = 13.0, 4.0 Hz, H_2_-6), 1.18 (1H, td, *J* = 13.0, 4.4 Hz, H_2_-3), 1.10 (1H, dd, *J* = 12.4, 3.0 Hz, H-5), 1.02 (1H, td, *J* = 13.0, 4.0 Hz, H_2_-1), 0.88 (3H, s, H_3_-18), 0.80 (3H, s, H_3_-19), 0.70 (3H, s, H_3_-20). ^13^C NMR (100 MHz, CDCl_3_) *δ*_C_ 165.0 (C-15, s), 157.9 (C-13, s), 148.5 (C-8, s), 138.3 (C-1ʹ, s), 129.0 (C-4ʹ, d), 124.0 (C-3ʹ/C-5ʹ, d), 119.7 (C-2ʹ/C-6ʹ, d), 117.9 (C-14, d), 106.4 (C-17), 56.1 (C-9, d), 55.5 (C-5, d), 42.1 (C-3, t), 39.9 (C-12, t), 39.7 (C-10, s), 39.1 (C-1, t), 38.4 (C-7, t), 33.6 (C-18, q), 33.6 (C-4, s), 24.5 (C-6, t), 21.7 (C-19, q), 21.5 (C-11, t), 19.4 (C-2, t), 18.6 (C-16, q), 14.5 (C-20, q); ESI MS: Calcd for C_26_H_37_NNaO, *m/z* 402.2767 [M + Na]^+^, found 402.2779.

#### (E)-N-Benzyl-3-methyl-5-((1S,4aS,8aS)-5,5,8a-trimethyl-2-methylene-decahydronaphthalen-1-yl)pent-2-enamide (4f)

The crude residue was pre-absorbed on silica gel and purified by flash silica gel column chromatography using a mixture of EtOAc:hexane (10:90) as an eluent to provide **4f** (30.7 mg, 97% yield) as a yellow oil. ${[\mathrm{\alpha }]}_{D}^{25}$ +31.0 (*c* 0.70, CHCl_3_); FTIR (neat) *Ѵ*_max_: 3295, 2925, 2843, 1657, 1631, 1536, 1454, 1387, 1365, 1255, 1175, 887, 697 cm^‒1^. ^1^H NMR (400 MHz, CDCl_3_)* δ* 7.31‒7.27 (5H, m, H-2ʹ/H-3ʹ/H-4ʹ/H-5ʹ/H-6ʹ), 5.63 (1H, br s, NH), 5.53 (1H, d, *J* = 1.1 Hz, H-14), 4.83 (1H, d, *J* = 1.4 Hz, H_2_-17), 4.48 (1H, s, H_2_-17), 4.47 (2H, m, H_2_-7ʹ), 2.36 (1H, ddd, *J* = 13.0, 4.4, 2.4 Hz, H_2_-7), 2.24 (1H, ddd, *J* = 13.0, 10.4, 1.2 Hz, H_2_-12), 2.18 (3H, d, *J* = 1.1 Hz, H_3_-16), 1.96 (1H, m, H_2_-7), 1.89 (1H, m, H_2_-12), 1.75 (1H, m, H_2_-1), 1.71 (1H, m, H_2_-6), 1.65 (1H, m, H_2_-11), 1.59 (1H, m, H_2_-2), 1.56 (1H, m, H-9), 1.49 (1H, m, H_2_-2), 1.44 (1H, m, H_2_-11), 1.39 (1H, m, H_2_-3), 1.31 (1H, dd, *J* = 13.0, 4.0 Hz, H_2_-6), 1.17 (1H, td, *J* = 13.0, 4.4 Hz, H_2_-3), 1.08 (1H, dd, *J* = 12.4, 3.0 Hz, H-5), 1.00 (1H, td, *J* = 13.0, 4.0 Hz, H_2_-1), 0.87 (3H, s, H_3_-18), 0.80 (3H, s, H_3_-19), 0.67 (3H, s, H_3_-20). ^13^C NMR (100 MHz, CDCl_3_) *δ* 167.9 (C-15, s), 156.9 (C-13, s), 149.4 (C-8, s), 139.5 (C-1ʹ, s), 129.5 (C-3ʹ/C-5ʹ, d), 128.7 (C-2ʹ/C-6ʹ, d), 128.2 (C-4ʹ, d), 118.2 (C-14, d), 107.0 (C-17, t), 56.5 (C-9, d), 55.8 (C-5, d),43.6 (C-7ʹ, t), 42.4 (C-3, t), 39.99 (C-10, s), 39.90 (C-12, t), 39.3 (C-1, t), 38.6 (C-7, t), 33.8 (C-4, s), 33.8 (C-18, q), 24.6 (C-6, t), 21.9 (C-19, q), 21.7 (C-11, t), 19.5 (C-2, t), 18.6 (C-16, q), 14.6 (C-20, q). ESI-TOF MS: calcd for C_27_H_39_NNaO, *m/z* 416.2924 [M + Na]^+^, found 416.2940.

#### (E)-3-Methyl-N-(pyridin-2-ylmethyl)-5-((1S,4aS,8aS)-5,5,8a-trimethyl-2-methylenedecahydronaphthalen-1-yl)pent-2-enamide (4g)

The crude residue was pre-absorbed on silica gel and purified by flash silica gel column chromatography using a mixture of EtOAc:hexane (20:80) as an eluent to provide **4g** (29.1 mg, 91% yield) as a yellow oil. ${[\mathrm{\alpha }]}_{D}^{27}$ +33.3 (c 0.71, CHCl3); FTIR (neat) *Ѵ*_max_: 3312, 3077, 2925, 2866, 2843, 1661, 1641, 1599, 1571, 1524, 1459, 1437, 1387, 1365, 1234, 1153, 887, 750, 692 cm^‒1^. ^1^H NMR (400 MHz, CDCl_3_) *δ* 8.54 (1H, ddd, *J* = 4.0, 1.6, 0.4 Hz, H-6ʹ), 7.67 (1H, td, *J* = 8.0, 2.0 Hz, H-4ʹ), 7.30 (1H, d, *J* = 8.0 Hz, H-3ʹ), 7.20 (1H, ddd, *J* = 4.0, 1.6, 0.8 Hz, H-5ʹ), 6.60 (1H, br s, NH), 5.65 (1H, br d, *J* = 1.3 Hz, H-14), 4.84, d, *J* = 1.6 Hz, H_2_-17), 4.61 (1H, br s, H_2_-7ʹ), 4.60 (1H, br s, H_2_-7ʹ), 4.50 (1H, d, *J* = 1.2 Hz, H_2_-17), 2.38 (1H, ddd, *J* = 13.0, 4.4, 2.4 Hz, H_2_-7), 2.26 (1H, ddd, *J* = 16.0, 10.4, 1.2 Hz, H_2_-12), 2.17 (3H, d, *J* = 1.2 Hz, H_3_-16), 1.98 (1H, dd, *J* = 13.0, 2.4 Hz, H_2_-7), 1.91 (1H, m, H_2_-12), 1.74 (1H, m, H_2_-1), 1.71 (1H, m, H_2_-6), 1.66 (1H, m, H_2_-11), 1.58 (1H, m, H_2_-2), 1.56 (1H, m, H-9), 1.50 (1H, m, H_2_-2), 1.46 (1H, m, H_2_-11), 1.39 (1H, m, H_2_-3), 1.32 (1H, dd, *J* = 13.0, 4.0 Hz, H_2_-6), 1.17 (1H, td, *J* = 13.0, 4.4 Hz, H_2_-3), 1.09 (1H, dd, *J* = 12.4, 3.0 Hz, H-5), 1.01 (1H, td, *J* = 13.0, 5.0 Hz, H_2_-1), 0.87 (3H, s, H_3_-18), 0.80 (3H, s, H_3_-19), 0.68 (3H, s, H_3_-20). ^13^C NMR (100 MHz, CDCl_3_) *δ* 167.2 (C-15, s), 156.8 (C-2ʹ, s), 155.6 (C-13, s), 149.0 (C-6ʹ, d), 148.5 (C-8, s), 136.8 (C-4ʹ, d), 122.31 (C-3ʹ, d) 122.24 (C-5ʹ, d), 117.6 (C-14, d), 106.3 (C-17, t), 56.1 (C-9, d), 55.5 (C-5), 44.3 (C-7ʹ, d), 42.1 (C-3, t), 39.7 (C-10, s), 39.7 (C-12, t), 39.1 (C-1, t), 38.3 (C-7, t), 33.6 (C-4, s), 33.6 (C-18, q), 24.5 (C-6, t), 21.7 (C-19, q), 21.5 (C-11, t), 19.4 (C-2, t), 18.4 (C-16, q), 14.5 (C-20, q). ESI-TOF MS: calcd for C_26_H_38_N_2_NaO, *m/z* 417.2876 [M + Na]^+^, found 417.2893.

#### (E)-N-(2-(1H-Indol-3-yl)ethyl)-3-methyl-5-((1S,4aS,8aS)-5,5,8a-trimethyl-2-methylenedecahydronaphthalen-1-yl)pent-2-enamide (4h)

The crude residue was pre-absorbed on silica gel and purified by flash silica gel column chromatography using a mixture of EtOAc:hexane (20:80) as an eluent to provide **4h** (33 mg, 91% yield) as a yellow oil. ${[\mathrm{\alpha }]}_{D}^{25}$ +26.2 (*c* 2.21, CHCl_3_); FTIR (neat) *Ѵ*_max_: 3299, 2928, 2845, 1689, 1641, 1521, 1457, 1440, 1387, 1365, 1254, 1230, 887, 740 cm^‒1^. ^1^H NMR (400 MHz, CDCl_3_) *δ* 8.30 (1H, br s, NH-tryptamine), 7.61 (1H, d, *J* = 8.0 Hz, H-7ʹ tryptamine), 7.37 (1H, d, *J* = 8.0 Hz, H-4ʹ tryptamine), 7.21 (1H, t, *J* = 7.0 Hz, H-5ʹ tryptamine), 7.11 (1H, t, *J* = 7.0 Hz, H-6ʹ tryptamine), 7.03 (1H, d, *J* = 2.0 Hz, H-2ʹ tryptamine), 5.49 (1H, br s, NH), 5.43 (1H, br s, H-14), 4.81 (1H, d, *J* = 1.2 Hz, H_2_-17), 4.47 (1H, br s, H_2_-17), 3.63 (2H, ddd, *J* = 7.0, 5.0, 2.0 Hz, H_2_-α tryptamine), 2.99 (2H, t, *J* = 7.0 Hz, H_2_-β tryptamine), 2.37 (1H, ddd, *J* = 13.0, 4.0, 3.0 Hz, H_2_-7), 2.20 (1H, ddd,* J* = 13.0, 10.0, 4.0 Hz, H_2_-12), 2.14 (3H, d,* J* = 0.8 Hz, H_3_-16), 1.95 (1H, m, H_2_-7), 1.86 (1H, m, H_2_-12), 1.74 (1H, m, H_2_-1), 1.70 (1H, m, H_2_-6), 1.63 (2H, m, H_2_-11), 1.57 (1H, m, H_2_-2), 1.54 (1H, m, H-9), 1.48 (1H, m, H_2_-2), 1.43 (1H, m, H_2_-11), 1.38 (1H, m, H_2_-3), 1.31 (1H, dd, *J* = 13.0, 4.4 Hz, H_2_-6), 1.16 (1H, td, *J* = 12.0, 4.1 Hz, H_2_-3), 1.07 (1H, dd, *J* = 12.0, 2.4 Hz, H-5), 0.99 (1H, td, *J* = 12.0, 4.0 Hz, H_2_-1), 0.87 (3H, s, H_3_-18), 0.80 (3H, s, H_3_-19), 0.67 (3H, s, H_3_-20); ^13^C NMR (100 MHz, CDCl_3_) *δ* 167.2 (C-15, s), 155.0 (C-13, s), 148.6 (C-8, s), 136.4 (C-7ʹa, s), 127.4 (C-3ʹa, s), 122.1 (C-2ʹ, d), 122.1 (C-6ʹ, d), 119.4 (C-5ʹ, d), 118.9 (C-7ʹ, d), 117.8 (C-14, d), 113.1 (C-3ʹ, s), 111.3 (C-4', d), 106.3 (C-17, t), 56.1 (C-9, d), 55.4 (C-5, d), 42.1 (C-3, t), 39.7 (C-10, s), 39.6 (C-12, t), 39.4 (C-α, d), 39.0 (C-1, t), 38.3 (C-7, t), 33.59 (C-18, q), 33.57 (C-4, s), 25.5 (C-β, t), 24.2 (C-6, t), 21.7 (C-19, q), 21.5 (C-11, d), 19.4 (C-2), 18.3 (C-16, q), 14.4 (C-20, q); ESI-TOF MS: calcd for C_30_H_42_N_2_NaO, *m/z* 469.3189 [M + Na]^+^, found 469.3197.

#### (E)-N-Methoxy-3-methyl-5-((1S,4aS,8aS)-5,5,8a-trimethyl-2-methylene-decahydronaphthalen-1-yl)pent-2-enamide (4i)

The crude residue was pre-absorbed on silica gel and purified by flash silica gel column chromatography using a mixture of EtOAc:hexane (20:80) as an eluent to provide **4i** (19 mg, 71% yield) as a yellow oil. ${[\mathrm{\alpha }]}_{D}^{27}$ +37.5 (*c* 1.54, CHCl_3_); FTIR (neat) *Ѵ*_max_: 3176, 2931, 2843, 1638, 1507, 1459, 1440, 1387, 1366, 1255, 1201, 1076, 1048, 887, 788 cm^‒1^. ^1^H NMR (400 MHz, CDCl_3_) *δ* 5.50 (1H, br s, H-14), 4.84 (1H, d, *J* = 1.2 Hz, H_2_-17), 4.49 (1H, s, H_2_-17), 3.77 (3H, s, OCH_3_), 2.38 (1H, ddd, *J* = 12.0, 4.4, 2.4 Hz, H_2_-7), 2.27 (1H, ddd, *J* = 13.0, 10.0, 4.4 Hz, H_2_-12), 2.17 (3H, d, *J* = 1.1 Hz, H_3_-16), 1.98 (1H, m, H_2_-7), 1.94 (1H, m, H_2_-12), 1.75 (1H, m, H_2_-1), 1.71 (1H, m, H_2_-6), 1.65 (1H, m, H_2_-11), 1.59 (1H, m, H_2_-2), 1.56 (1H, m, H-9), 1.49 (1H, m, H-2), 1.48 (1H, m, H_2_-11), 1.39 (1H, m, H_2_-3), 1.32 (1H, dd, *J* = 13.0, 4.2 Hz, H_2_-6), 1.17 (1H, td, *J* = 13.0, 4.0 Hz, H_2_-3), 1.09 (1H, dd, *J* = 12.0, 2.4 Hz, H-5), 1.01 (1H, td, *J* = 12.8, 4.0 Hz, H_2_-1), 0.87 (3H, s, H_3_-18), 0.80 (3H, s,H_3_-19), 0.68 (3H, s, H_3_-20); ^13^C NMR (100 MHz, CDCl_3_) *δ* 171.0 (C-15, s), 148.4 (C-13/C-8, s), 113.0 (C-14, d), 106.3 (C-17, t), 64.7 (OCH_3_, q), 56.1 (C-9, d), 55.5 (C-5, d), 42.1 (C-3, t), 39.9 (C-10, s), 39.7 (C-12, t), 39.1 (C-1, t), 38.3 (C-7, t), 33.6 (C-18, s), 33.59 (C-4, s), 24.4 (C-6, t), 21.7 (C-19, s), 21.5 (C-11, t), 19.4 (C-2, t), 18.8 (C-16, s), 14.5 (C-20, s); ESI-TOF MS: calcd for C_21_H_35_NNaO_2_, *m/z* 356.2560 [M + Na]^+^, found 356.2565.

#### (E)-N-Hydroxy-3-methyl-5-((1S,4aS,8aS)-5,5,8a-trimethyl-2-methylene-decahydronaphthalen-1-yl)pent-2-enamide (4j)

The crude residue was pre-absorbed on silica gel and purified by flash silica gel column chromatography using a mixture of EtOAc:hexane (40:80) as an eluent to provide **4j** (4.7 mg, 20% yield) as a yellow oil. ${[\mathrm{\alpha }]}_{D}^{26}$ +33.5 (*c* 0.55, CHCl_3_); FTIR (neat) *Ѵ*_max_: 3212, 2925, 2843, 1641, 1459, 1442, 1387, 1366, 1087, 1031, 887, 861, 738 cm^‒1^; ^1^H NMR (300 MHz, CDCl_3_) *δ* 5.45 (1H, br s, H-14), 4.84 (1H, d, *J* = 1.3 Hz, H_2_-17), 4.47 (1H, br s, H_2_-17), 2.38 (1H, ddd, *J* = 13.0, 4.0, 2.0 Hz, H_2_-7), 2.27 (1H, ddd, *J* = 14.0, 10.0, 5.0 Hz, H_2_-12), 2.18 (3H, br s, H_3_-16), 1.97 (1H, m, H-7), 1.93 (1H, m, H_2_-12), 1.75 (1H, m, H_2_-1), 1.71 (1H, m, H_2_-6), 1.65 (1H, m, H_2_-11), 1.58 (1H, m, H_2_-2), 1.55 (1H, m, H-9), 1.50 (1H, m, H_2_-2), 1.46 (1H, m, H_2_-11), 1.39 (1H, m, H_2_-3), 1.32 (1H, dd, *J* = 13.0, 4.0 Hz, H_2_-6), 1.17 (1H, td, *J* = 13.3, 4.0 Hz, H_2_-3), 1.08 (1H, dd, *J* = 10.0, 3.0 Hz, H-5), 1.00 (1H, td, *J* = 12.0, 4.0 Hz, H_2_-1), 0.87 (3H, s, H_3_-18), 0.80 (3H, s, H_3_-19), 0.68 (3H, s, H_3_-20); ^13^C NMR (75 MHz, CDCl_3_) *δ* 166.5 (C-15, s), 158.7 (C-13, s), 148.3 (C-8, s), 112.3 (C-14, d), 106.4 (C-17, t), 56.0 (C-9, d), 55.5 (C-5, d), 42.1 (C-3, t), 39.72 (C-12, t), 39.70 (C-10, s), 39.1 (C-1, t), 38.3 (C-7, t), 33.6 (C-18, q), 33.6 (C, C-4, s), 24.4 (C-6, t), 22.6 (C-19, q), 21.7 (C-11, t), 19.3 (C-16, q), 18.9 (C-2, t), 14.5 (C-20, q); ESI-TOF MS: calcd for C_20_H_34_NO_2_, *m/z* 320.2584 [M + H]^+^, found 320.2583.

#### (E)-3-Methyl-5-((1S,4aS,8aS)-5,5,8a-trimethyl-2-methylenedecahydro-naphthalen-1-yl)pent-2-enehydrazide (4k)

The crude residue was pre-absorbed on silica gel and purified by flash silica gel column chromatography using a mixture of EtOAc:hexane (20:80) as an eluent to provide **4k** (9.8 mg, 40% yield) as a yellow oil. ${[\mathrm{\alpha }]}_{D}^{27}$ +35.5 (*c* 1.16, CHCl_3_); FTIR (neat) *Ѵ*_max_: 3240, 2924, 2865, 2844, 1655, 1624, 1542, 1458, 1440, 1386, 1365, 1289, 1194, 1006, 887, 851, 685 cm^‒1^; ^1^H NMR (400 MHz, CDCl_3_) *δ* 5.48 (1H, s, H-14), 4.84 (1H, d, *J* = 1.3 Hz, H_2_-17), 4.47 (1H, br s, H_2_-17), 2.38 (1H, ddd, *J* = 13.0, 4.0, 2.0 Hz, H_2_-7), 2.27 (1H, ddd, *J* = 14.0, 10.0, 5.0 Hz, H_2_-12), 2.18 (3H, br s, H_3_-16), 1.97 (1H, m, H-7), 1.93 (1H, m, H_2_-12), 1.75 (1H, m, H_2_-1), 1.71 (1H, m, H_2_-6), 1.65 (1H, m, H_2_-11), 1.58 (1H, m, H_2_-2), 1.55 (1H, m, H-9), 1.50 (1H, m, H_2_-2), 1.46 (1H, m, H_2_-11), 1.39 (1H, m, H_2_-3), 1.32 (1H, dd, *J* = 13.0, 4.0 Hz, H_2_-6), 1.17 (1H, td, *J* = 13.3, 4.0 Hz, H_2_-3), 1.08 (1H, dd, *J* = 10.0, 3.0 Hz, H-5), 1.00 (1H, td, *J* = 12.0, 4.0 Hz, H_2_-1), 0.87 (3H, s, H_3_-18), 0.80 (3H, s, H_3_-19), 0.68 (3H, s, H_3_-20);^13^C NMR (100 MHz, CDCl_3_) *δ* 163.0 (C-15, s), 157.1 (C-13, s), 148.4 (C-8, s), 114.6 (C-14, d), 106.3 (C-17, t), 56.0 (C-9, d), 55.5 (C-5, d), 42.1 (C-3, t), 39.67 (C-10, s), 39.6 (C-12, t), 39.0 (C-1, t), 38.3 (C-7, t), 33.59 (C-18, q), 33.58 (C-4, s), 21.7 (C-19, q), 21.6 (C-11, t), 19.4 (C-16, q), 18.6 (C-2, t), 14.5 (C-20, q); ESI-TOF MS: calcd for C_20_H_34_N_2_O, *m/z* 341.2563 [M + H]^+^, found 341.2553.

#### (E)-3-Methyl-N-((tetrahydro-2H-pyran-2-yl)oxy)-5-((1S,4aS,8aS)-5,5,8a-trimethyl-2-methylenedecahydronaphthalen-1-yl)pent-2-enamide (4l)

The crude residue was pre-absorbed on silica gel and purified by flash silica gel column chromatography using a mixture of EtOAc:hexane (10:90) as an eluent to provide **4l** (22.9 mg, 74% yield) as a yellow oil. ${[\mathrm{\alpha }]}_{D}^{25}$ +11.8 (*c* 1.06 CHCl_3_); FTIR (neat) *Ѵ*_max_: 3197, 2925, 2849, 1661, 1642, 1456, 1441, 1387, 1365, 1257, 1204, 1113, 1037, 1064, 1021, 951, 895, 875, 817 cm^‒1^. ^1^H NMR (400 MHz, CDCl_3_) *δ* 5.49 (1H, br s, H-14), 4. 94 (1H, br s, H-1ʹ), 4.82 (1H, s, H_2_-17), 4.47 (1H, s, H_2_-17), 3.95 (1H, t, *J* = 9.0 Hz, H_2_-5ʹ), 3.63 (1H, m, H_2_-5ʹ), 2.37 (1H, ddd, *J* = 13.0, 4.0, 2.4 Hz, H_2_-7), 2.25 (1H, ddd, *J* = 14.0, 10.1, 4 Hz, H_2_-12), 2.15 (3H, d, *J* = 1.0 Hz, H_3_-16), 1.97 (1H, m, H_2_-7), 1.93 (1H, m, H_2_-12), 1.81 (2H, m, H_2_-2ʹ/H_2_-3ʹ), 1.74 (1H, m, H_2_-1), 1.71 (1H, m, H_2_-6), 1.65 (1H, m, H_2_-11), 1.63 (1H, m, H_2_-4ʹ), 1.58–1.53 (2H, m, H_2_-3ʹ/H_2_-4ʹ), 1.58 (1H, m, H_2_-2), 1.54 (1H, m, H-9), 1.48 (1H, m, H_2_-2), 1.46 (1H, m, H_2_-11), 1.38 (1H, m, H_2_-3), 1.30 (1H, dd, *J* = 13.0, 4.0 Hz, H_2_-6), 1.16 (1H, td, *J* = 13.0, 4.0 Hz, H_2_-3), 1.07 (1H, dt, *J* = 12.0, 2.0 Hz, H-5), 0.99 (1H, br t, *J* = 12.0 Hz, H_2_-1), 0.86 (3H, s, H_3_-18), 0.79 (3H, s, H_3_-19), 0.67 (3H, s, H_3_-20); ^13^C NMR (100 MHz, CDCl_3_) *δ* 158.0 (C-15, s), 158.0 (C-13, s), 148.4 (C-8, s), 148.4 (C-13, s), 113.2 (C-14, d), 106.3 (C-17, t), 102.6 (C-1ʹ, d), 62.6 (C-5ʹ, t), 56.0 (C-9, d), 55.4 (C-5, d), 42.0 (C-3, t), 39.8 (C-10, s), 39.6 (C-12, t), 39.0 (C-1, t), 38.3 (C-7, t), 33.6 (C-18, q), 33.6 (C-4, s), 28.1 (C-2ʹ, t), 25.0 (C-4ʹ, t), 24.4 (C-6, t), 21.7 (C-19, q), 21.4 (C-11, t), 19.3 (C-2, t), 18.67 (C-3ʹ, t), 18.66 (C-16, q), 14.4 (C-20, q). ESI-TOF MS: calcd for C_25_H_41_NNaO_3_,* m/z* 426.2979 [M + Na]^+^, found 426.2969.

#### (E)-N,N,3-Trimethyl-5-((1S,4aS,8aS)-5,5,8a-trimethyl-2-methylenedecahydro-naphthalen-1-yl)pent-2-enamide (4m)

The crude residue was pre-absorbed on silica gel and purified by flash silica gel column chromatography using a mixture of CH_2_Cl_2_:MeOH (98:2) as an eluent to provide **4m** (22.6 mg, 85% yield) as a yellow oil. ${[\mathrm{\alpha }]}_{D}^{27}$ +31.9 (*c* 2.11, CHCl_3_); FTIR (neat) *Ѵ*_max_: 2925, 2848, 1651, 1629, 1458, 1442, 1388, 1371, 1265, 1131, 1056, 887, 852 cm^‒1^. ^1^H NMR (300 MHz, CDCl_3_) *δ* 5.76 (1H, d, *J* = 0.8 Hz, H-14), 4.84 (1H, d, *J* = 1.6 Hz, H_2_-17), 4.50 (1H, d, *J* = 1.2 Hz, H_2_-17), 3.02 (3H, s, H_3_-1ʹʹ), 2.98 (3H, s, H_3_-1ʹ), 2.39 (1H, ddd, *J* = 13.0 4.4, 2.4 Hz, H_2_-7), 2.24 (1H, ddd, *J* = 13.0, 10.0, 4.0 Hz, H_2_-12), 1.97 (1H, m, H_2_-7), 1.93 (1H, m, H_2_-12), 1.90 (3H, d, *J* = 1.2 Hz, H_3_-16), 1.75 (1H, m, H_2_-1), 1.71 (1H, m, H_2_-7), 1.65 (1H, m, H_2_-11), 1.58 (1H, m, H-9), 1.50 1.58 (1H, m, H-9), 1.55 (1H, m, H_2_-2), 1.50 (1H, m, H_2_-2), 1.48 (1H, m, H_2_-11), 1.39 (1H, m, H_2_-3), 1.33 (1H, dd, *J* = 13.0, 4.0 Hz, H_2_-6), 1.17 (1H, td, *J* = 13.0, 4.0 Hz, H_2_-3), 1.08 (1H, dd, *J* = 12.0, 3.0 Hz, H-5), 1.01 (1H, td, *J* = 13.0, 4.0 Hz, H_2_-1), 0.87 (3H, s, H_3_-18), 0.80 (3H, s, H_3_-19), 0.68 (3H, s, H_3_-20). ^13^C NMR (75 MHz, CDCl_3_) *δ* 168.8 (C-15, s), 149.9 (C-13, s), 148.5 (C-8, s), 117.4 (C-14, d), 106.3 (C-17, t), 56.1 (C-9, d), 55.5 (C-5, d), 42.1 (C-3, t), 39.7 (C-10, s), 39.1 (C-1, t), 38.6 (C-12, t), 38.3 (C-7, t), 37.8 (C-1ʹʹ, q), 34.7 (C-1ʹ, q), 33.61 (C-18, q), 33.58 (C-4, s), 24.4 (C-6, t), 21.7 (C-19, q), 21.4 (C-11, t), 19.4 (C-2, t), 18.5 (C-16, q), 14.5 (C-20, q); ESI-TOF MS: calcd for C_22_H_38_NO, *m/z* 322.2948 [M + H]^+^, found 332.2949.

#### (E)-3-Methyl-1-(piperidin-1-yl)-5-((1S,4aS,8aS)-5,5,8a-trimethyl-2-methylenedecahydronaphthalen-1-yl)pent-2-en-1-one (4n)

The crude residue was pre-absorbed on silica gel and purified by flash silica gel column chromatography using a mixture of CH_2_Cl_2_:MeOH (99:1) as an eluent to provide **4n** (20.3 mg, 68% yield) as a yellow oil. ${[\mathrm{\alpha }]}_{D}^{27}$ +24.9 (*c* 2.08, CHCl_3_); FTIR (neat) *Ѵ*_max_: 3077, 2932, 2848, 1626, 1440, 1381, 1255, 1228, 1137, 1124, 1021, 886, 850 cm^‒1^. ^1^H NMR (400 MHz, CDCl_3_) *δ* 5.72 (1H, d, *J* = 0.8 Hz, H-14), 4.84 (1H, d, *J* = 1.6 Hz, H_2_-17), 4.51 (1H, br s, H_2_-17), 3.59 (1H, m, H_2_-a), 3.45 (1H, m, H_2_-a), 2.39 (1H, ddd, *J* = 13.0, 4.0, 2.4 Hz, H_2_-7), 2.22 (1H, ddd, *J* = 14.0, 9.5, 4.3 Hz, H_2_-7), 1.94 (2H, m, H_2_-12), 1.83 (3H, br s, H_3_-16), 1.76 (1H, m, H_2_-1), 1.71 (1H, m, H_2_-c), 1.65 (1H, m, H_2_-11), 1.63 (1H, m, H_2_-6), 1.61 (1H, m, H_2_-b), 1.58 (2H, m, H_2_-c/H-9), 1.55 (1H, m, H_2_-2), 1.53 (1H, m, H_2_-b), 1.52 (1H, m, H_2_-b), 1.49 (1H, m, H_2_-2), 1.47 (2H, m, H_2_-11), 1.39 (1H, br d, *J* = 13.0 Hz, H_2_-3), 1.32 (1H, dd, *J* = 13.0, 4.0 Hz, H_2_-6), 1.17 (1H, td, *J* = 13.0, 4.0 Hz, H_2_-3), 1.07 (1H, dd, *J* = 13.0, 2.4 Hz, H-5), 1.00 (1H, td, *J* = 13.0, 4.0 Hz, H_2_-1), 0.87 (3H, s, H_3_-18), 0.80 (3H, s, H_3_-19), 0.69 (3H, s, H_3_-20); ^13^C NMR (100 MHz, CDCl_3_) *δ* 167.5 (C-15, s), 148.4 (C-8, s), 147.9 (C-13, s), 118.1 (C-14, d), 106.4 (C-17, t), 56.1 (C-9, d), 55.6 (C-5, d), 47.4 (C-a of piperidine, t), 42.21 (C-a of piperidine, t), 42.16 (C-3, t), 39.7 (C-10, s), 39.1 (C-1, t), 38.4 (C-12, t), 38.2 (C-7, t), 33.64 (C-18, q), 33.58 (C-4, s), 26.7 (C-b of piperidine, t), 25.7 (C-b of piperidine, t), 24.7 (C-c of piperidine, t), 24.5 (C-6, t), 21.7 (C-19, q), 21.3 (C-11, t), 19.4 (C-2, t), 18.5 (C-16, q), 14.5 (C-20, q); ESI-TOF MS: calcd for C_25_H_41_NNaO, *m/z* 394.3080 [M + Na]+, found 394.3070.

#### (E)-N-Methoxy-N,3-dimethyl-5-((1S,4aS,8aS)-5,5,8a-trimethyl-2-methylene-decahydronaphthalen-1-yl)pent-2-enamide (4o)

The crude residue was pre-absorbed on silica gel and purified by flash silica gel column chromatography using a mixture of CH_2_Cl_2_:MeOH (99:1) as an eluent to provide **4o** (29.4 mg, quantitative yield) as a colorless oil. ${[\mathrm{\alpha }]}_{D}^{27}$ +29.6 (*c* 2.61, CHCl_3_); FTIR (neat) *Ѵ*_max_: 2933, 2844, 1656, 1634, 1458, 1441, 1387, 1366, 1319, 1175, 1003, 887 cm^‒1^; ^1^H NMR (300 MHz, CDCl_3_) *δ* 6.08 (1H, s, H-14), 4.86 (1H, br s, H_2_-17), 4.53 (1H, br s, H_2_-17), 3.68 (3H, s, OCH_3_), 3.20 (3H, s, H_3_-1ʹ), 2.40 (1H, ddd, *J* = 12.0, 4.0, 3.0 Hz, H_2_-7), 2.30 (1H, ddd,* J* = 14.0, 9.0, 4.0 Hz, H_2_-12), 2.13 (3H, d, *J* = 1.1 Hz, H_3_-16), 2.06–1.91 (2H, m, H_2_-7/H_2_-12), 1.78 (1H, m, H_2_-1), 1.74 (1H, m, H_2_-11), 1.65 (1H, m, H_2_-2), 1.56 (1H, m, H-9), 1.50 (1H, m, H_2_-2), 1.47 (1H, m, H_2_-11), 1.40 (1H, m, H_2_-3), 1.33 (1H, dd, *J* = 13.0, 4.0 Hz, H_2_-6), 1.20 (1H, td, *J* = 13.0, 4.0 Hz, H_2_-3), 1.10 (1H, dd, *J* = 12.0, 2.0 Hz, H-5), 1.01 (1H, td, *J* = 13.0, 4.0 Hz, H_2_-1), 0.88 (3H, s, H_3_-18), 0.81 (3H, s, H_3_-19), 0.70 (3H, s, H_3_-20); ^13^C NMR (75 MHz, CDCl_3_) *δ* 168.3 (C-15, s), 157.2 (C-13, s), 148.4 (C-8, s), 113.7 (C-14, d), 106.3 (C-17, t), 61.4 (OCH_3_, q), 56.0 (C-9, d), 55.5 (C-5, d), 42.1 (C-3, t), 40.0 (C-12, t), 39.7 (C-10, s), 39.0 (C-1, t), 38.3 (C-7, t), 33.59 (C-18, q), 33.57 (C-4, s), 32.0 (C-1ʹ, q), 24.5 (C-6, t), 21.7 (C-19, q), 21.6 (C-11, t), 19.4 (C-2, t), 18.7 (C-16, q), 14.5 (C-20, q); ESI-TOF MS: calcd for C_22_H_37_NNaO_2_, *m/z* 370.2717 [M + Na]^+^, found 370.2741.

#### (E)-N-Hydroxy-N,3-dimethyl-5-((1S,4aS,8aS)-5,5,8a-trimethyl-2-methylene decahydronaphthalen-1-yl)pent-2-enamide (4p)

The crude residue was pre-absorbed on silica gel and purified by flash silica gel column chromatography using a mixture of CH_2_Cl_2_:MeOH (99:1) as an eluent to provide **4p** (27.5 mg, 51% yield) as a yellow oil. ${[\mathrm{\alpha }]}_{D}^{27}$ +38.8 (*c* 0.42, CHCl_3_); FTIR (neat) *Ѵ*_max_:3170, 3078, 2926, 2843, 1764, 1687, 1643, 1597, 1440, 1387, 1201, 1110, 965, 886, 742 cm^‒1^. ^1^H NMR (400 MHz, CDCl_3_) *δ* 5.73 (1H, br s, NH), 5.68 (1H, d, *J* = 1.0 Hz, H-14), 4.85 (1H, d, *J* = 1.4 Hz, H_2_-17), 4.50 (1H, s, H_2_-17), 3.35 (3H, s, H_3_-1ʹ), 2.39 (1H, ddd, *J* = 13.0, 4.4, 2.4 Hz, H_2_-7), 2.28 (H, ddd, *J* = 12.0, 10.0, 4.0 Hz, H_2_-12), 2.04 (3H, s, H_3_-16), 1.98 (1H, m, H_2_-12), 1.94 (1H, m, H_2_-7), 1.75 (1H, m, H_2_-1), 1.73 (1H, m, H_2_-6), 1.66 (1H, m, H_2_-11), 1.60 (1H, m, H_2_-2). 1.56 (1H, m, H-9), 1.50 (H, m, H_2_-2), 1.47 (1H, m, H_2_-11), 1.39 (1H, m, H_2_-3), 1.33 (1H, dd, *J* = 13.0, 4.0 Hz, H_2_-6), 1.18 (1H, td, *J* = 13.0, 4.0 Hz, H_2_-3), 1.09 (1H, dd, *J* = 12.0, 4.0 Hz, H-5), 1.01 (1H, td, *J* = 13.0, 4 Hz, H_2_-1), 0.87 (3H, s, H_3_-18), 0.80 (3H, s, H_3_-19), 0.68 (3H, s, H_3_-20); ^13^C NMR (100 MHz, CDCl_3_) *δ* 163.5 (C-15, s), 148.4 (C-13/C-8, s), 114.9 (C-14, d), 106.4 (C-17, t), 56.1 (C-9, d), 55.5 (C-5, d), 42.1 (C-3, t), 40.0 (C-10, s), 39.7 (C-12, t), 39.1 (C-1, t), 38.3 (C-7, t), 36.3 (C-1ʹ, q), 33.6 (C-18, q), 33.59 (C-4, s), 24.5 (C-6, t), 21.7 (C-19, q), 21.5 (C-11, t), 19.4 (C-2, t), 18.7 (C-16, q), 14.5 (C-20, q). ESI-TOF MS: calcd for C_21_H_35_NNaO_2_, *m/z* 356.2560 [M–H]^+^, found 356.2564.

#### Methyl ((E)-3-methyl-5-((1S,4aS,8aS)-5,5,8a-trimethyl-2-methylenedeca-hydronaphthalen-1-yl)pent-2-enoyl)glycinate (4q)

The crude residue was pre-absorbed on silica gel and purified by flash silica gel column chromatography using a mixture of EtOAc:hexane (10:90) as an eluent to provide **4q** (22.3 mg, 74% yield) as a yellow oil. ${[\mathrm{\alpha }]}_{D}^{26}$ + 29.0 (*c* 0.88, CHCl_3_); FTIR (neat) *Ѵ*_max_: 3314, 2928, 2865, 2844, 1754, 1661, 1638, 1638, 1532, 1438, 1366, 1205, 1175, 887 cm^‒1^; ^1^H NMR (400 MHz, CDCl_3_) *δ* 5.86 (1H, br s, NH), 5.59 (1H, br s, H-14), 4.84 (1H, d, *J* = 1.4 Hz, H_2_-17), 4.49 (1H, d, *J* = 1.0 Hz, H_2_-17), 4.10 (2H, d, *J* = 5.2 Hz, H_2_-2ʹ), 3.77 (3H, s, OCH_3_), 2.39 (1H, ddd, *J* = 12.0, 4.4, 2.4 Hz, H_2_-7), 2.26 (1H, ddd, *J* = 12.0, 10.0, 4.0 Hz, H_2_-12), 2.15 (3H, d, *J* = 0.8 Hz, H_3_-16), 1.98 (1H, m, H_2_-7), 1.92 (1H, m, H_2_-12), 1.76 (1H, m, H_2_-1), 1.72 (1H, m, H_2_-6), 1.65 (1H, m, H_2_-6), 1.5589 (1H, m, H_2_-2), 1.56 (1H, m, H-9), 1.50 (1H, m, H_2_-2), 1.46 (1H, m, H_2_-11), 1.39 (1H, m, H_2_-3), 1.32 (1H, dd, *J* = 13.0, 4.4 Hz, H_2_-6), 1.17 (1H, td, *J* = 13.0, 4.0 Hz, H_2_-3), 1.09 (1H, dd, *J* = 13.0, 3.0 Hz, H-5), 1.01 (1H, td, *J* = 13.0, 4.4 Hz, H_2_-1), 0.87 (3H, s, H_3_-18), 0.80 (3H, s, H_3_-19), 0.68 (3H, s, H_3_-20); ^13^C NMR (100 MHz, CDCl_3_) *δ* 170.8 (C-1ʹ, s), 167.0 (C-15, s), 156.8 (C-13, s), 148.4 (C-8, s), 116.9 (C-14, d), 106.3 (C-17, t), 56.1 (C-9, d), 55.4 (C-5, d), 52.3 (OCH_3_, q), 42.1 (C-3, t), 41.0 (C-2ʹ, t), 39.7 (C-12, t), 39.68 (C-10, s), 39.1 (C-1, t), 38.3 (C-7, t), 33.6 (C-18, q), 33.6 (C-4, s), 24.5 (C-6, t), 21.7 (C-19, q), 21,5 (C-11, t), 19.4 (C-2, t), 18.5 (C-16, q), 14.5 (C-20, q); ESI-TOF MS: calcd for C_23_H_37_NNaO_3_, *m/z* 398.2666 [M + Na]^+^, found 398.2660.

#### Ethyl ((E)-3-methyl-5-((1S,4aS,8aS)-5,5,8a-trimethyl-2-methylenedec-ahydronaphthalen-1-yl) pent-2-enoyl)glycinate (4r)

The crude residue was pre-absorbed on silica gel and purified by flash silica gel column chromatography using a mixture of CH_2_Cl_2_:MeOH (95:5) as an eluent to provide **4r** (22.4 mg, 72% yield) as a colourless oil.${[\mathrm{\alpha }]}_{D}^{27}$ +30.5 (*c* 1.41 CHCl_3_); FTIR (neat) *Ѵ*_max_: 3313, 2928, 2844, 1750, 1661, 1638, 1524, 1460, 1443, 1373, 1263, 1195, 1178, 1025, 886, 863, 736 cm^‒1^; ^1^H NMR (300 MHz, CDCl_3_) *δ*_H_ 5.94 (H, br s, NH), 5.60 (H, d, *J* = 1.0 Hz, H-14), 4.85 (1H, d, *J* = 1.2 Hz, H_2_-17), 4.50 (1H, s, H_2_-17), 4.23 (2H, q, *J* = 7.0 Hz, H_2_-3ʹ), 4.08 (2H, d, *J* = 5.0 Hz, H_2_-2ʹ), 2.39 (1H, ddd, *J* = 13.0, 4.0, 2.0 Hz, H_2_-7), 2.26 (1H, ddd, *J* = 13.0, 10.0, 4.0 Hz, H_2_-12), 2.15 (3H, d, *J* = 1.0 Hz, H_3_-16), 1.98 (1H, m, H_2_-7), 1.94 (1H, m, H_2_-12), 1.78 (1H, m, H_2_-1), 1.72 (1H, m, H_2_-6), 1.68 (1H, m, H_2_-11), 1.61 (1H, m, H_2_-2), 1.56 (1H, m, H-9), 1.50 (1H, m, H_2_-2), 1.48 (1H, m, H_2_-11), 1.40 (1H, m, H_2_-3), 1.33 (1H, m, H-6), 1.30 (3H, t, *J* = 7.0 Hz, H_3_-4ʹ), 1.21 (1H, td, *J* = 13.0, 4.0 Hz, H_2_-3), 1.10 (1H, dd, *J* = 12.0, 3.0 Hz, H-5), 0.99 (1H, td, *J* = 13.0, 4.0 Hz, H_2_-1), 0.88 (3H, s, H_3_-18), 0.81 (3H, s, H_3_-19), 0.69 (3H, s, H_3_-20); ^13^C NMR (75 MHz, CDCl_3_) *δ*_C_ 170.3 (C-1ʹ, s), 167.0 (C-15, s), 156.5 (C-13, s), 148.4 (C-8, s), 117.0 (C-14, d), 106.3 (C-17, t), 61.4 (C-3ʹ, t), 56.1 (C-9, d), 55.4 (C-5, d), 42.1 (C-3, t), 41.2 (C-2ʹ, t), 39.68 (C-12, t), 39.65 (C-10, s), 39.0 (C-1, t), 38.3 (C-7, t), 33.5 (C-18, q), 33.5 (C-4, s), 24.4 (C-6, t), 21.7 (C-19, q), 21.5 (C-11, t), 19.3 (C-2, t), 18.4 (C-16, q), 14.4 (C-20, q), 14.1 (C-4ʹ, q); ESI-TOF MS: calcd for C_24_H_39_NNaO_3_, *m/z* 412.2822 [M + Na]^+^, found 412.2836.

#### The synthesis of ((E)-3-methyl-5-((1S,4aS,8aS)-5,5,8a-trimethyl-2-methylenedecahydro-naphthalen-1-yl)pent-2-enoyl)glycine (4s)

A mixture of **4r** (16.5 mg, 0.04 mmol, 1.0 equiv.) and lithium hydroxide monohydrate (2.1 mg, 0.5 mmol, 1.2 equiv.) were dissolved in THF:MeOH:H_2_O (0.4 mL, 0.1 M). The reaction was allowed to stir at room temperature for 4 h, and then MeOH and THF were removed under vacuo. The crude was diluted with H_2_O and extracted with EtOAc, and the organic layer was discarded. The aqueous layer was acidified to ~ pH 3 with 1N hydrochloric acid solution, and then extracted with EtOAc. The organic layers were combined, washed with brine, dried over Mg_2_SO_4_ (s), and dried under vacuo to afford **4s** (11.7 mg, quantitative yield) as a colourless oil. The target compound was obtained as a yellow oil in quantitative yield. ${[\mathrm{\alpha }]}_{D}^{25}$ + 20.7 (*c* 0.83 CHCl_3_); FTIR (neat) *Ѵ*_max_: 3336, 2926, 2867, 1715, 1662, 1541, 1457, 1441, 1388, 1366, 1261, 1218, 1087, 888, 733 cm^‒1^; ^1^H NMR (400 MHz, CDCl_3_) *δ* 6.11 (1H, br s, NH), 5.61 (1H, br s, H-14), 4.84 (1H, br s, H_2_-17), 4.48 (1H, br s, H_2_-17), 4.10 (2H, br s, H_2_-2ʹ), 2.39 (1H, br d, *J* = 12.0 Hz, H_2_-7), 2.25 (1H, br d, *J* = 12.0 Hz, H_2_-12), 2.15 (3H, s, H_3_-16), 1.97 (1H, m, H_2_-7), 1.91 (1H, m, H_2_-12), 1.75 (1H, m, H_2_-1), 1.71 (1H, m, H_2_-6), 1.65 (1H, m, H_2_-11), 1.58 (1H, m, H_2_-2), 1.56 (1H, m, H-9), 1.50 (1H, m, H_2_-2), 1.45 (1H, m, H_2_-11), 1.39 (1H, m, H_2_-3), 1.32 (1H, m, H_2_-6), 1.17 (1H, m, H_2_-3), 1.08 (1H, br d, *J* = 12.0 Hz, H-5), 1.00 (1H, m, H_2_-1), 0.87 (3H, s, H_3_-18), 0.80 (3H, s, H_3_-19), 0.68 (3H, s, H_3_-20); ^13^C NMR (100 MHz, CDCl_3_) *δ* 172.9 (C-1ʹ, s), 167.9 (C-15, s), 158.0 (C-13, s), 148.4 (C-8, s), 116.5 (C-14, d), 106.3 (C-17, t), 56.1 (C-9, d), 55.4 (C-5, d), 42.1 (C-3, t), 41.1 (C-2ʹ, t), 39.8 (C-10, s), 39.7 (C-12, t), 39.0 (C-1, t), 38.3 (C-7, t), 33.6 (C-18, q), 33.6 (C, C-4, s), 24.4 (C-6, t), 21.7 (C-19, q), 21.5 (C-11, t), 19.4 (C-2, t), 18.6 (C-16, q), 14.5 (C-20, q); ESI-TOF MS: calcd for C_22_H_36_NO_3_, *m/z* 362.2690 [M + H]^+^, found 362.2681.

### Cytotoxic activity assay

The cells were seeded into 96-well plates *(*100 µL for adherent cells and 75 µL for suspended cells*)* at a density of 5000–20,000 cells per well, depending on their growth rates. Adherent and suspended cells were then allowed to grow at 37 °C with 95% humidity and 5% CO_2_ for 24 h and 30 min, respectively. The cytotoxicity assay was initiated by adding an equal volume of cell culture medium containing tested compound at predetermined concentrations. Following 48 h of exposure, cell viability was determined using MTT assay for adherent cells^51,52^or XTT assay for suspeended cells^53^. Briefly, for adherent cells, 100 µL of the MTT reagent was added to each well, and the microtiter plates were further incubated for 2.5–4 h. The medium was subsequently replaced with 100 µL of DMSO to dissolve the purple formazan before the absorbance at 550 nm was measured using a microplate reader *(*a SpectraMax Plus 384*)* with a reference wavelength of 650 nm. For suspended cells, 75 µL of the XTT reagent was added to each well, and the cells were further incubated for 4 h. Afterwards, the absorbance of orange formazan at 492 nm was measured with a reference wavelength of 690 nm using a microplate reader. The IC_50_ value was finally calculated from the dose–response curve as the concentration that inhibits the cell growth by 50*%* in comparison with the negative control following 48 h of exposure to each tested compound.

### Cell death analysis

Annexin V and 7-AAD double staining was used to distinguish apoptotic and non-apoptotic cell death modes. Briefly, MDA-MB-231 cells were seeded into 6-well culture plates at 1 × 10^6^ cells/well and left in a CO_2_ incubator for 24 h. Subsequently, the cells were treated with tested compounds for 24–48 h. DMSO was used as a vehicle and its final concentration was kept at 0.2% (v/v) throughout the study. The treated cells were harvested by trypsinization and subjected to annexin V/7-AAD double staining using Muse® Annexin V & Dead Cell Kit as previously described^54^. The stained cells were analyzed by flow cytometric technique using Muse® Cell Analyzer. Population of annexin V-positive cells was defined as early apoptotic cells, and the population positive for both annexin V and 7-AAD was defined as late apoptotic cells. The sequential events of increasing early and late apoptotic cell populations over time is a characteristic of apoptotic cell death.

### Western blot analysis

MDA-MB-231 cells in 6-well culture plates (1 × 10^6^ cells/well) were treated with tested compounds for 24 h. Then the cells were harvested and western blot analysis was used to detect alteration of targeted proteins in the treated cells, as previously described^55^. Briefly, the cells in 6-well culture plates were scraped in RIPA cell lysis buffer supplemented with protease/phosphatase inhibitor cocktail before being lysed by sonication. Cell lysates were centrifuged at 12,000×*g* for 5 min, at 4 °C, to remove cell debris. Total proteins (20 μg) in the cell lysates were separated by SDS-PAGE and blotted to PVDF membranes. The membranes were blocked with 3% (w/v) BSA and incubated overnight at 4 °C with primary antibodies specific to the following proteins: EGFR, phospho-EGFR (Y845), FAK, phospho-FAK (Y397), Akt, phospho-Akt (S473), ERK, and phospho-ERK (T202/Y204). Subsequently, the membranes were incubated with horseradish peroxidase-conjugated secondary antibodies for 1 h at room temperature. Bands of specific proteins were detected by using SuperSignal™ ECL substrates and visualized on X-ray films.

### Molecular docking

iGEMDOCK v2.1 software^56^ with accurate docking setting was employed to perform molecular docking to predict the possible binding between **4l** and FAK. The molecular structure of **4l** was obtained at the B3LYP/6-31G* level using Gaussian09. The protein structures of FERM domain (PDB:2AL6) and kinase domain (PDB ID: 2JKK) of FAK were obtained from Protein Data Bank (http://www.rcsb.org/). The BIOVIA Discovery Studio Visualizer^57^ was used to analyze and image the docking results.

### Physicochemical property methodology

Drug-likeness properties and Lipinski’s rule of five were studied by using SwissADME website services^58,59^.

## Conclusions

**ACP** is a labdane diterpenoid that could be obtained in a high quantity from *K. elegans*. With its attractive structure to use as a starting point for optimization into novel natural product-based bioactive agents, a series of 21 **ACP** derivatives were synthesized and evaluated for their in vitro cytotoxic activity against a panel of cancer cell lines. **ACP** and most of its derivatives showed moderate activity. Interestingly, compound **4l** demonstrated notable cytotoxic activity against the triple-negative breast cancer MDA-MB-231 cell line. Further mechanistic study revealed FAK as a potential target of compound **4l** in MDA-MB-231 breast cancer cells, and FAK inhibition appeared to be the mechanism underlying non-apoptotic cell death induction in **4l**-treated cells. An in-silico study suggests that **4l** could potentially inhibit FAK activation by binding to the ATP binding pocket of FAK kinase domain, resulting in suppression of Tyr397 autophosphorylation of FAK. To our knowledge, this is the first report demontrating FAK inhibitory activity of **ACP** derivative. The data obtained from this work is important for further development of natural product-based bioactive agents for treatment of the triple-negative breast cancer based on the **ACP** scaffold.

### Supplementary Information

Supplementary Information.

## Data Availability

The data generated and/or analyzed during the current study are available in the Supplementary Information. These included the un-cropped films of western blot results, and the ^1^H, ^13^CNMR, and MS spectra of compounds **1**–**3** and **4a**–**4s**.
