# Supplementary material for: The amide derivative of anticopalic acid induces non-apoptotic cell death in triple-negative breast cancer cells by inhibiting FAK activation
Source: Sci Rep. 2023 Aug 18;13:13456. doi: 10.1038/s41598-023-40669-6 (PMC10439230; doi:10.1038/s41598-023-40669-6)

Supplementary Information

The amide derivative of anticopalic acid induces non-apoptotic cell death in triple-negative breast cancer cells by inhibiting FAK activation

Pornsuda Chawengrum[#,1], Natthaorn Luepongpatthana[#,2], Sanit Thongnest[3,4], Jitnapa Sirirak[5], Jutatip Boonsombat[3,4,*], Kriengsak Lirdprapamongkol[4,6,*], Siriporn Keeratichamroen[6], Patcharin Kongwaen[3], Phreeranat Montatip[6], Prasat Kittakoop[1,3,4], Jisnuson Svasti[2,6] & Somsak Ruchirawat[1,3,4]

[1]*Chemical Biology Program, Chulabhorn Graduate Institute, Chulabhorn Royal Academy, Bangkok, Thailand*

[2]*Applied Biological Sciences Program, Chulabhorn Graduate Institute, Chulabhorn Royal Academy, Bangkok, Thailand*

[3]*Laboratory of Natural Products, Chulabhorn Research Institute, Bangkok, Thailand*

[4]*Center of Excellence on Environmental Health and Toxicology (EHT), Office of the Permanent Secretary (OPS), Ministry of Higher Education, Science, Research and Innovation (MHESI), Thailand*

[5]*Department of Chemistry, Faculty of Science, Silpakorn University, Nakhon Pathom, Thailand*

[6]*Laboratory of Biochemistry, Chulabhorn Research Institute, Bangkok, Thailand*

[#] *Contributed equally*

*Corresponding authors jutatip@cri.or.th and kriengsak@cri.or.th

Content	Page
**Figure S1.** The Un-cropped films of western blot results of cell survival signaling proteins in MDA-MB-231 cells after treatment with compound **4l** for 24 h.	**S4**
**Figure S2.** The Un-cropped films of WB results of FAK phosphorylation in MDA-MB-231 cells after treatment with FAK specific inhibitor (FAKi) for 24 h.	**S4**
**Figure S3.** Dose-dependent cytotoxic curves of compound **4l** (a) and a positive control doxorubicin (b).	**S5**
**Figure S4.** The HPLC chromatogram showing percentage purity of compound **4l**.	**S6**
**Table S1.** Selectivity index values of the isolated compounds towards different human cancer cell lines.	**S7**
^{1}H and ^{13}C NMR spectra of anticopalic acid (**1**)	**S8**
NMR and HRMS spectra of compound **2**	**S10**
NMR and HRMS spectra of compound **3**	**S13**
NMR and HRMS spectra of compound **4a**	**S16**
NMR and HRMS spectra of compound **4b**	**S19**
NMR and HRMS spectra of compound **4c**	**S22**
NMR and HRMS spectra of compound **4d**	**S25**
NMR and HRMS spectra of compound **4e**	**S28**
NMR and HRMS spectra of compound **4f**	**S31**
NMR and HRMS spectra of compound **4g**	**S34**
NMR and HRMS spectra of compound **4h**	**S37**
NMR and HRMS spectra of compound **4i**	**S40**
NMR and HRMS spectra of compound **4j**	**S43**
NMR and HRMS spectra of compound **4k**	**S46**
NMR and HRMS spectra of compound **4l**	**S49**
NMR and HRMS spectra of compound **4m**	**S52**
NMR and HRMS spectra of compound **4n**	**S55**
NMR and HRMS spectra of compound **4o**	**S58**

NMR and HRMS spectra of compound **4p** **S61**

NMR and HRMS spectra of compound **4q** **S64**

NMR and HRMS spectra of compound **4r** **S67**

NMR and HRMS spectra of compound **5s** **S70**

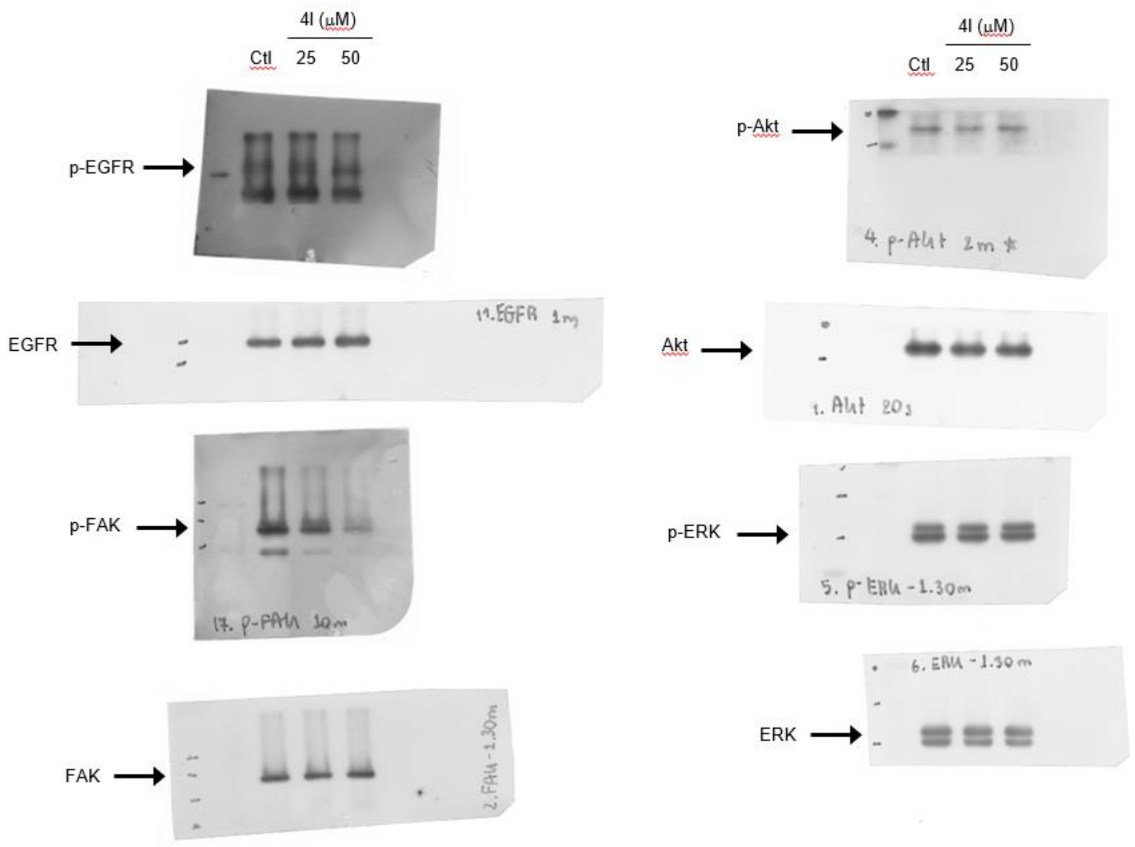

Figure S1. The Un-cropped films of western blot results of cell survival signaling proteins in MDA-MB-231 cells after treatment with compound **4l** for 24 h.

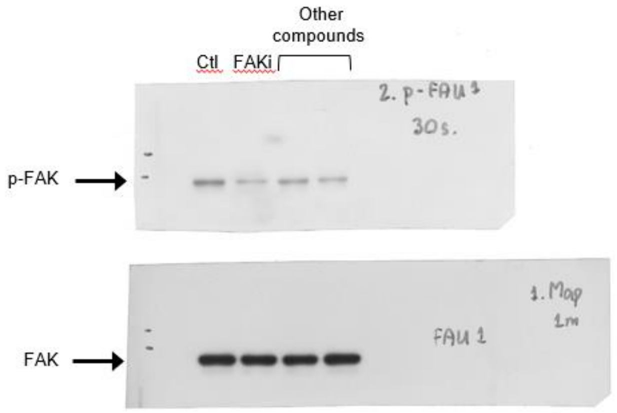

Figure S2. The Un-cropped films of WB results of FAK phosphorylation in MDA-MB-231 cells after treatment with FAK specific inhibitor (FAKi) for 24 h.

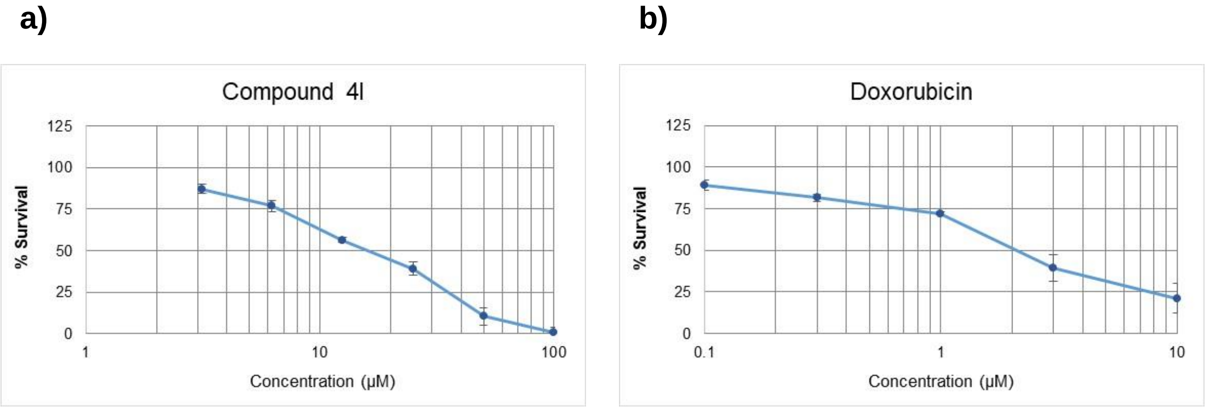

Figure S3. Dose-dependent cytotoxic curves of compound **4l** (a) and a positive control doxorubicin (b). Cell survival was determined after 48 h treatment by MTT assay. Data are expressed as mean ± SD of triplicate wells. Curves of a representative experiment are shown.

HPLC analysis system: Hichrom 5 C18 (25 cm x 4.6 mm; Hichrom)

95% MeCN/H_2O to 100% MeCN

1 ST-Copa140-(C15)

Sample Name:	**ST-Copa140-(C15)**	*Injection Volume:*	**20.0**
Vial Number:	**7**	*Channel:*	**UV_VIS_1**
Sample Type:	**unknown**	*Wavelength:*	**210.0**
Control Program:	**G 95to100_100m F07 ACN**	*Bandwidth:*	**4**
Quantif. Method:	**Method**	*Dilution Factor:*	**1.0000**
Recording Time:	**6/28/2023 14:34**	*Sample Weight:*	**1.0000**
Run Time (min):	**24.92**	*Sample Amount:*	**1.0000**

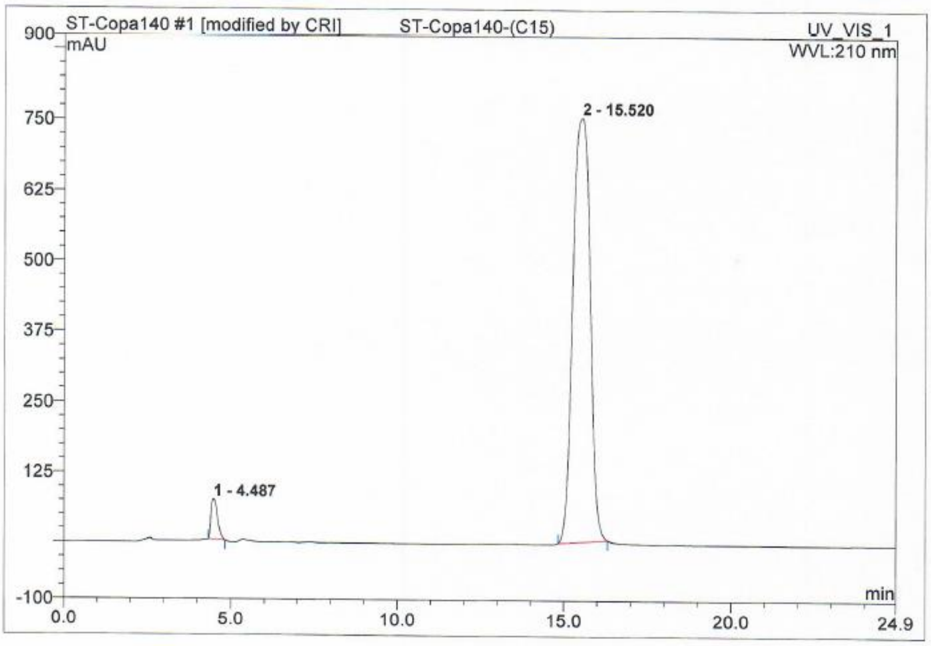

No.	Ret.Time min	Peak Name	Height mAU	Area mAU*min	Rel.Area %	Amount	Type
1	4.49	n.a.	71.375	16.219	3.46	n.a.	BMB*
2	15.52	n.a.	752.344	452.448	96.54	n.a.	BMB*
Total:			823.718	468.667	100.00	0.000	

Figure S4. The HPLC chromatogram showing percentage purity of compound **4l**.

Table S1 Selectivity index values of the isolated compounds towards different human cancer cell lines.

Cpds	**Selectivity Index (SI) values**								
	MDA-MB-231[a]	T47-D[b]	HepG2[c]	HL-60[d]	MOLT-3[e]	A549[f]	H69AR[g]	HuCCA-1[h]	HeLa[i]
**1**	1.0	1.0	1.0	1.6	2.5	1.0	1.0	1.0	1.1
**2**	1.7	1.0	1.0	2.0	1.7	0.9	0.5	1.1	1.4
**3**	2.0	1.3	1.0	2.2	3.3	0.9	0.7	1.0	1.6
**4a**	1.9	0.8	0.8	2.3	2.2	0.9	0.5	0.9	1.2
**4b**	1.3	1.0	1.4	6.4	5.7	1.0	1.0	1.0	1.8
**4c**	1.8	0.6	0.6	2.0	2.1	0.9	0.4	0.9	1.7
**4d**	1.0	1.0	1.0	1.0	1.0	1.0	1.0	1.0	1.0
**4e**	1.0	1.0	1.0	2.2	5.1	2.8	1.0	1.0	1.3
**4f**	1.0	1.0	1.0	1.5	1.0	1.0	1.0	1.0	1.0
**4g**	1.3	0.8	1.1	2.0	2.1	1.2	0.5	1.0	1.0
**4h**	2.1	0.4	1.2	2.9	2.8	1.2	0.7	1.1	2.3
**4i**	1.2	1.3	1.2	2.1	2.5	1.2	0.5	1.1	1.0
**4j**	1.0	1.0	1.0	1.0	1.0	1.0	1.0	1.0	1.0
**4k**	1.5	0.9	0.9	1.4	5.0	0.9	0.9	1.1	1.2
**4l**	2.9	0.6	0.5	1.9	1.5	0.7	0.4	0.7	1.5
**4m**	1.3	1.0	0.8	2.1	1.9	0.8	0.4	0.9	1.0
**4n**	1.1	0.5	1.2	1.9	1.8	0.9	0.7	0.9	1.0
**4o**	1.2	1.0	0.8	1.7	1.6	0.6	0.4	0.9	0.9
**4p**	2.8	2.0	1.1	10.2	18.8	1.1	0.7	1.2	2.1
**4q**	1.4	0.9	0.9	2.5	4.4	1.5	1.1	0.8	1.7
**4r**	2.7	0.7	1.0	2.7	5.6	0.9	1.4	0.9	1.7
**4s**	1.0	1.0	-	1.8	1.6	1.0	1.0	1.0	1.0
**DOX**	0.3	1.6	1.9	-	-	2.0	0.0	1.0	1.6
**ETO**	-	-	-	-	-	-	-	-	-

[a] = Triple-negative breast cancer; [b] = Hormone-dependent breast cancer; [c] = Hepatocellular carcinoma; [d] = Acute promyelocytic leukemia; [e] = T cell acute lymphoblastic leukemia; [f] = Lung adenocarcinoma; [g] = multidrug-resistant small cell lung carcinoma; [h] = cholangiocarcinoma derived from Thai patient; [i] = Cervical carcinoma; DOX = doxorubicin; ETO = etoposide; SI = IC_{50} normal cell/ IC_{50} cancer cell; - = Not determined

^{1}H NMR of compound **1** (600 MHz, $CDCl_3$)

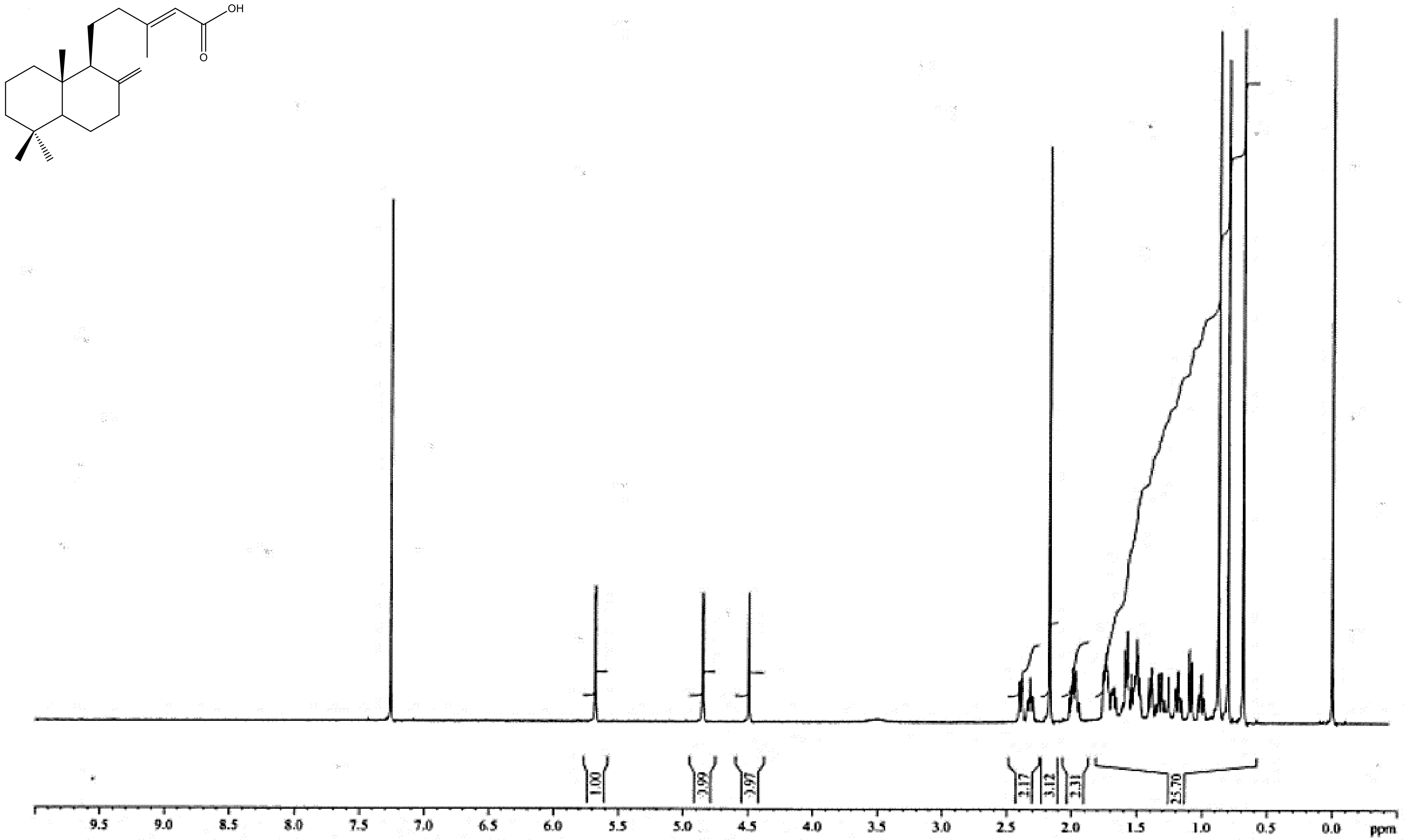

^{13}C NMR of compound **1** (150 MHz, $CDCl_3$)

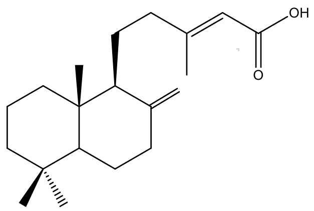

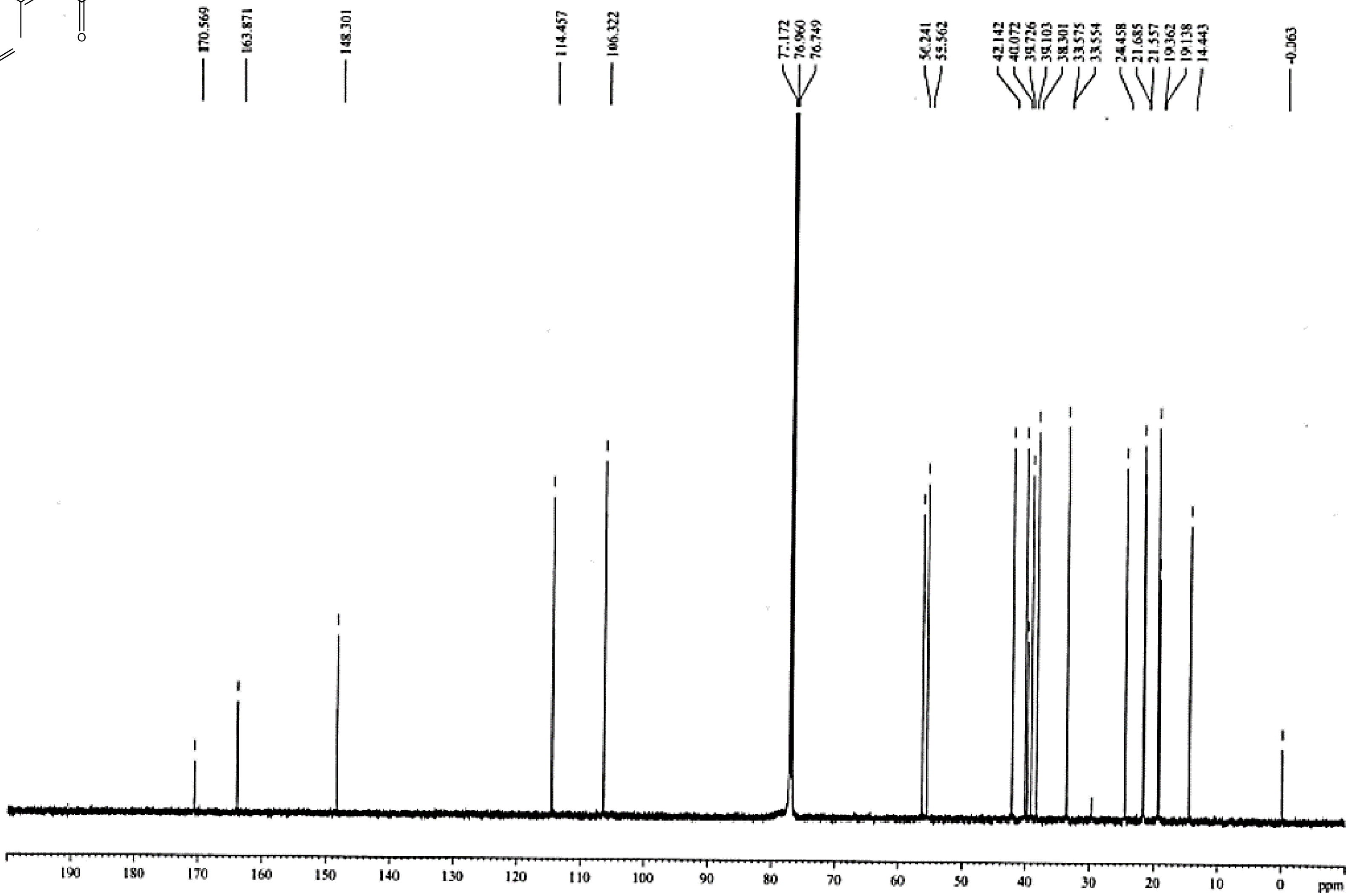

^{1}H NMR of compound **2** (600 MHz, $CDCl_3$)

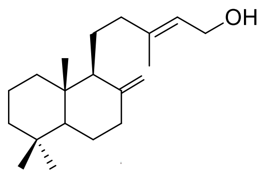

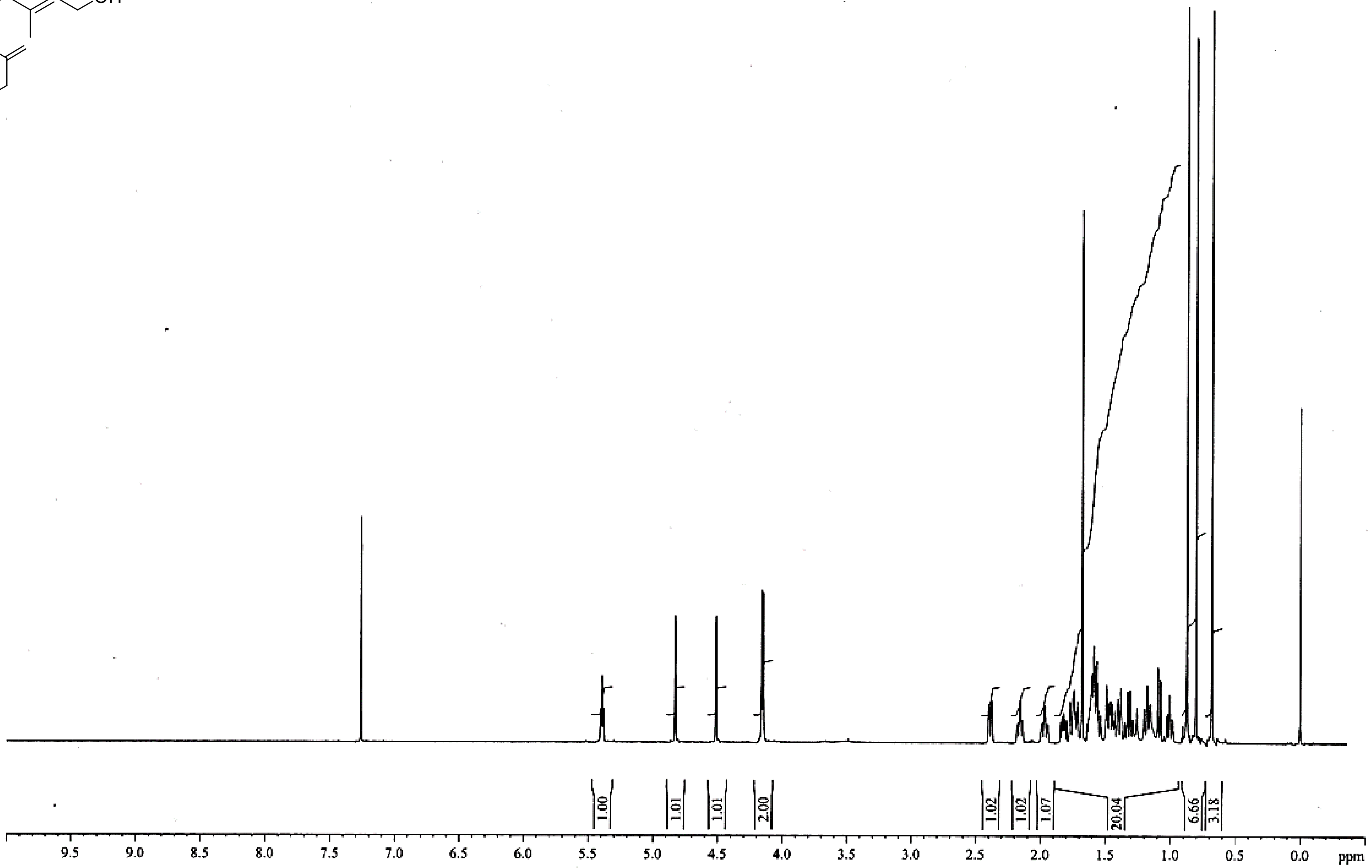

^{13}C NMR of compound **2** (600 MHz, $CDCl_3$)

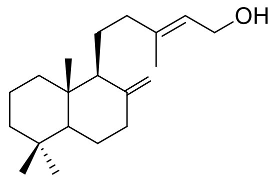

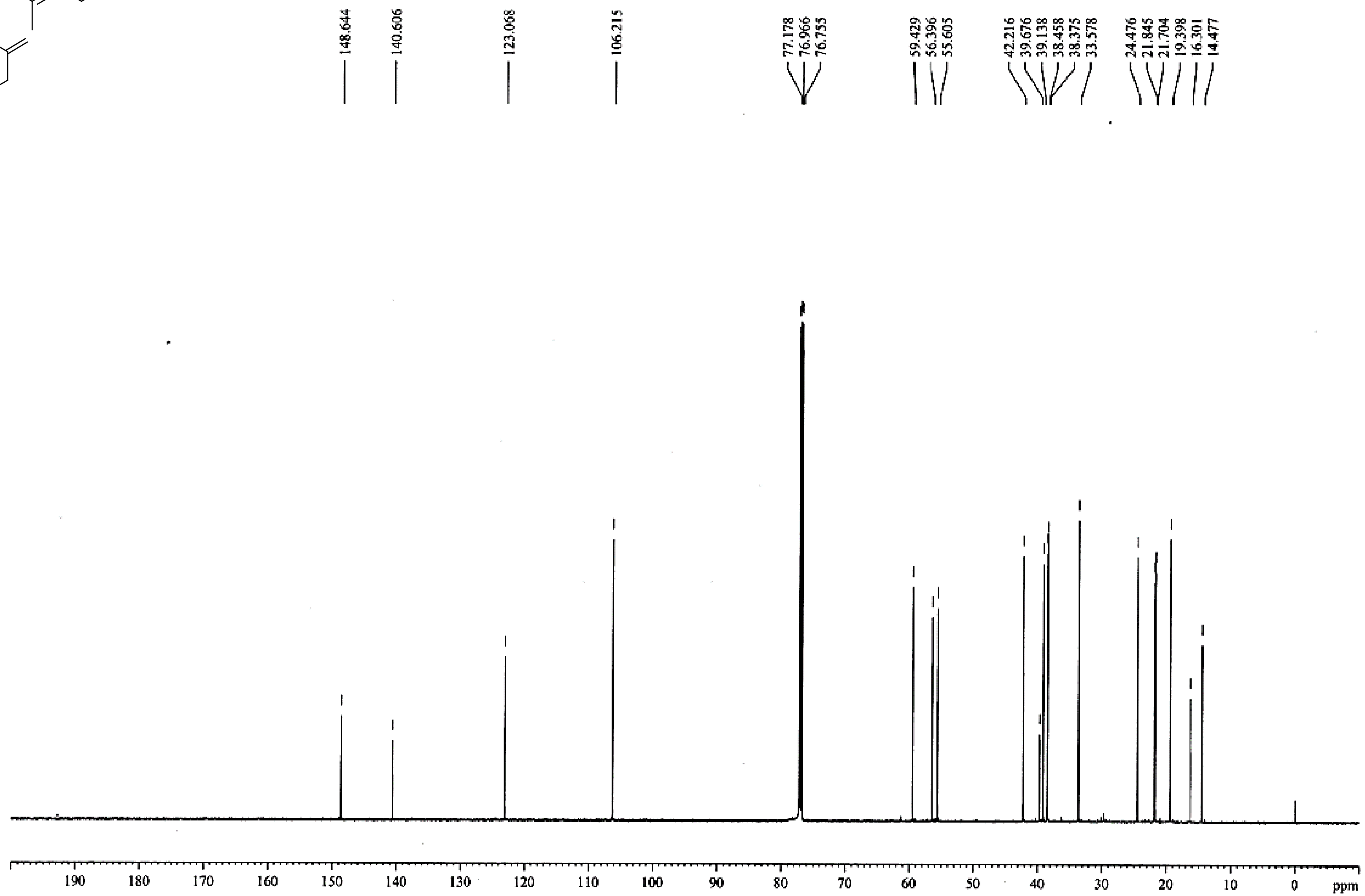

Mass spectrum of compound **2**

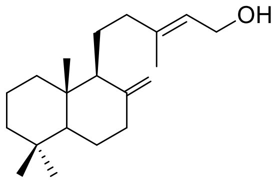

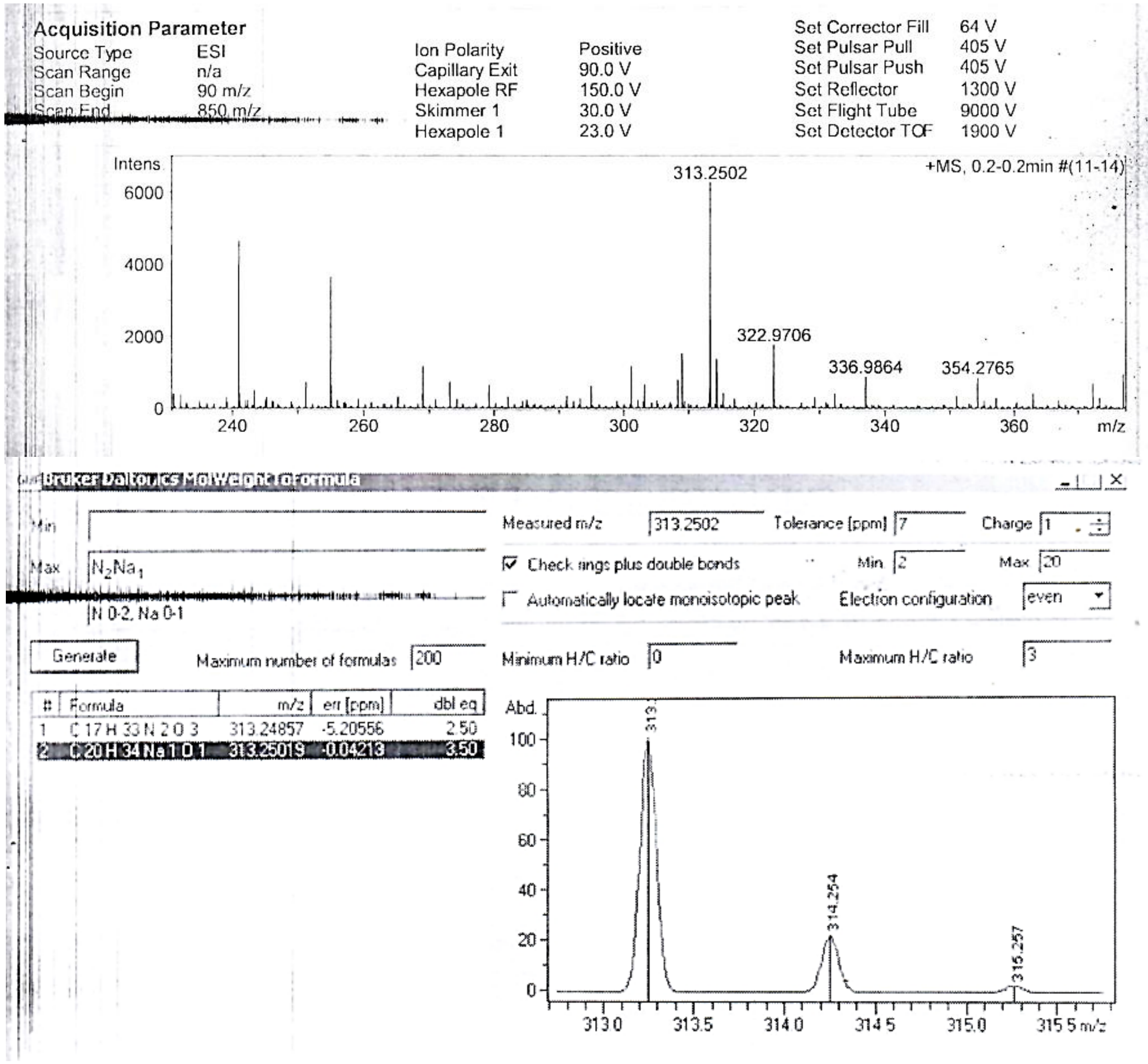

^{1}H NMR of compound **3** (400 MHz, $CDCl_3$)

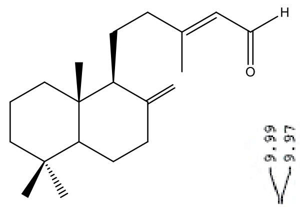

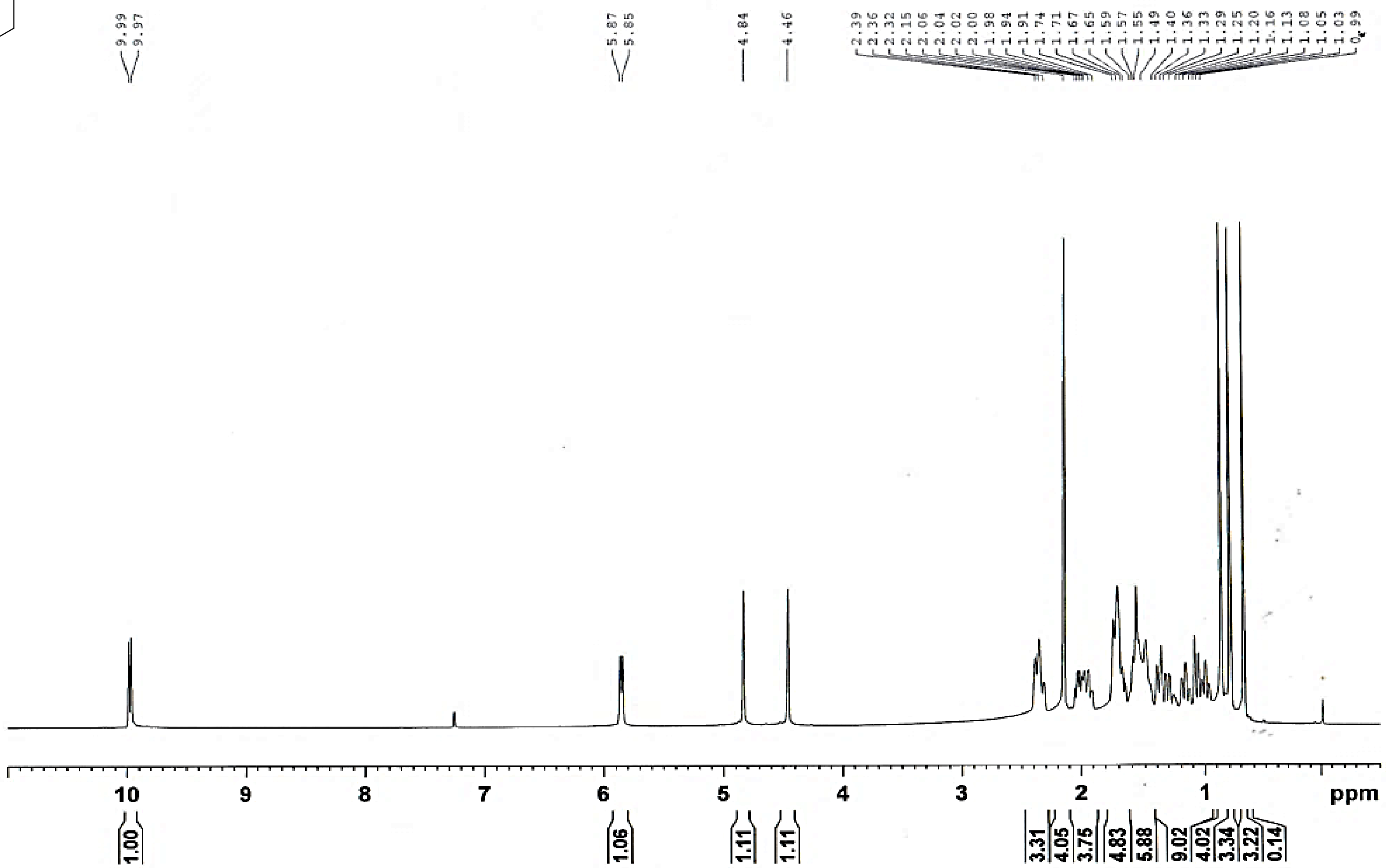

^{13}C NMR of compound **3** (100 MHz, $CDCl_3$)

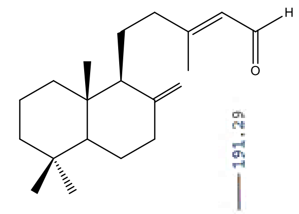

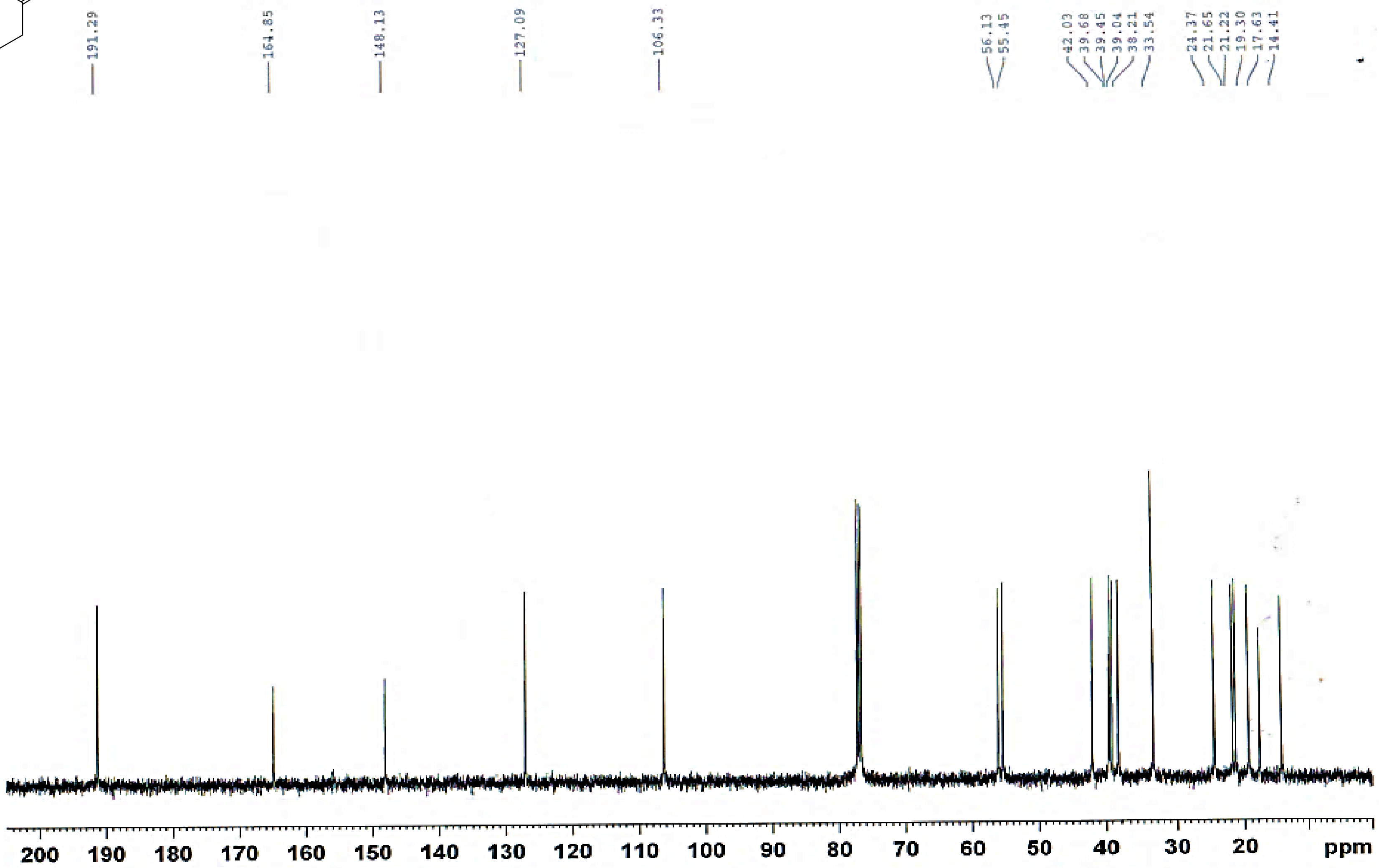

Mass spectrum of compound **3**

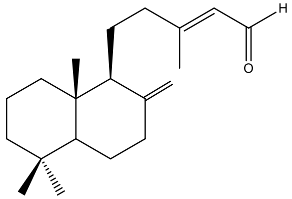

Acquisition Parameter

Source Type	ESI	Ion Polarity	Positive	Set Nebulizer	0.5 Bar
Focus	Not active	Set Capillary	4500 V	Set Dry Heater	89 °C
Scan Begin	50 m/z	Set End Plate Offset	-500 V	Set Dry Gas	6.0 l/min
Scan End	800 m/z	Set Charging Voltage	2000 V	Set Divert Valve	Source
		Set Corona	0 nA	Set APCI Heater	0 °C

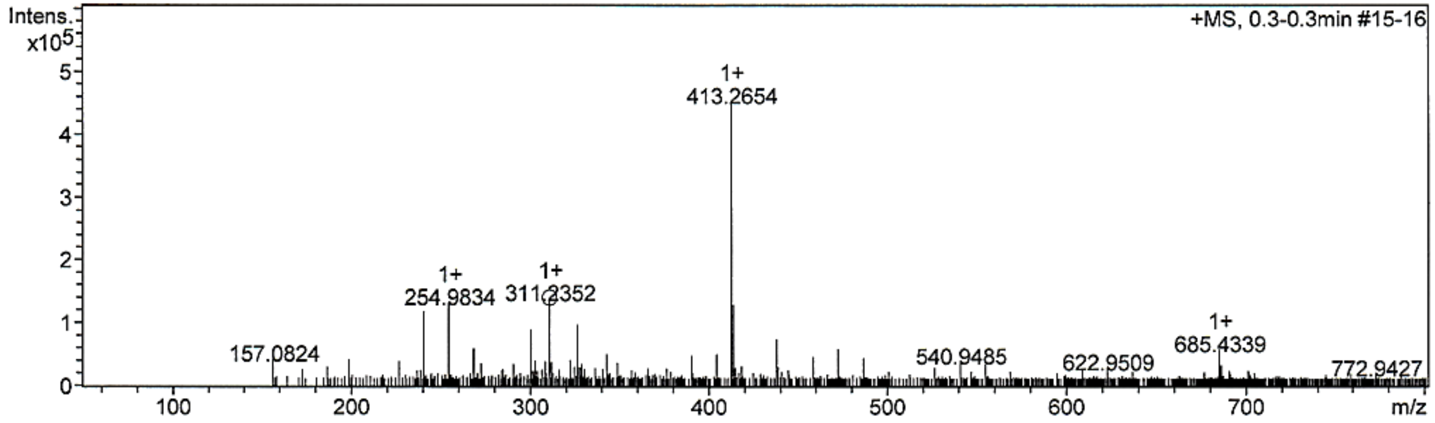

Meas. m/z	#	Ion Formula	Score	m/z	err [mDa]	err [ppm]	mSigma	rdb	e¯ Conf	N-Rule	Adduct
311.235221	1	C20H32NaO	100.00	311.234536	-0.7	-2.2	14.8	4.5	even	ok	M+H

^{1}H NMR of compound **4a** (400 MHz, $CDCl_3$)

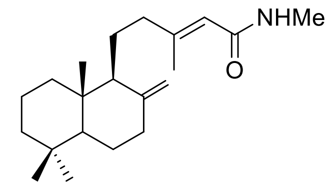

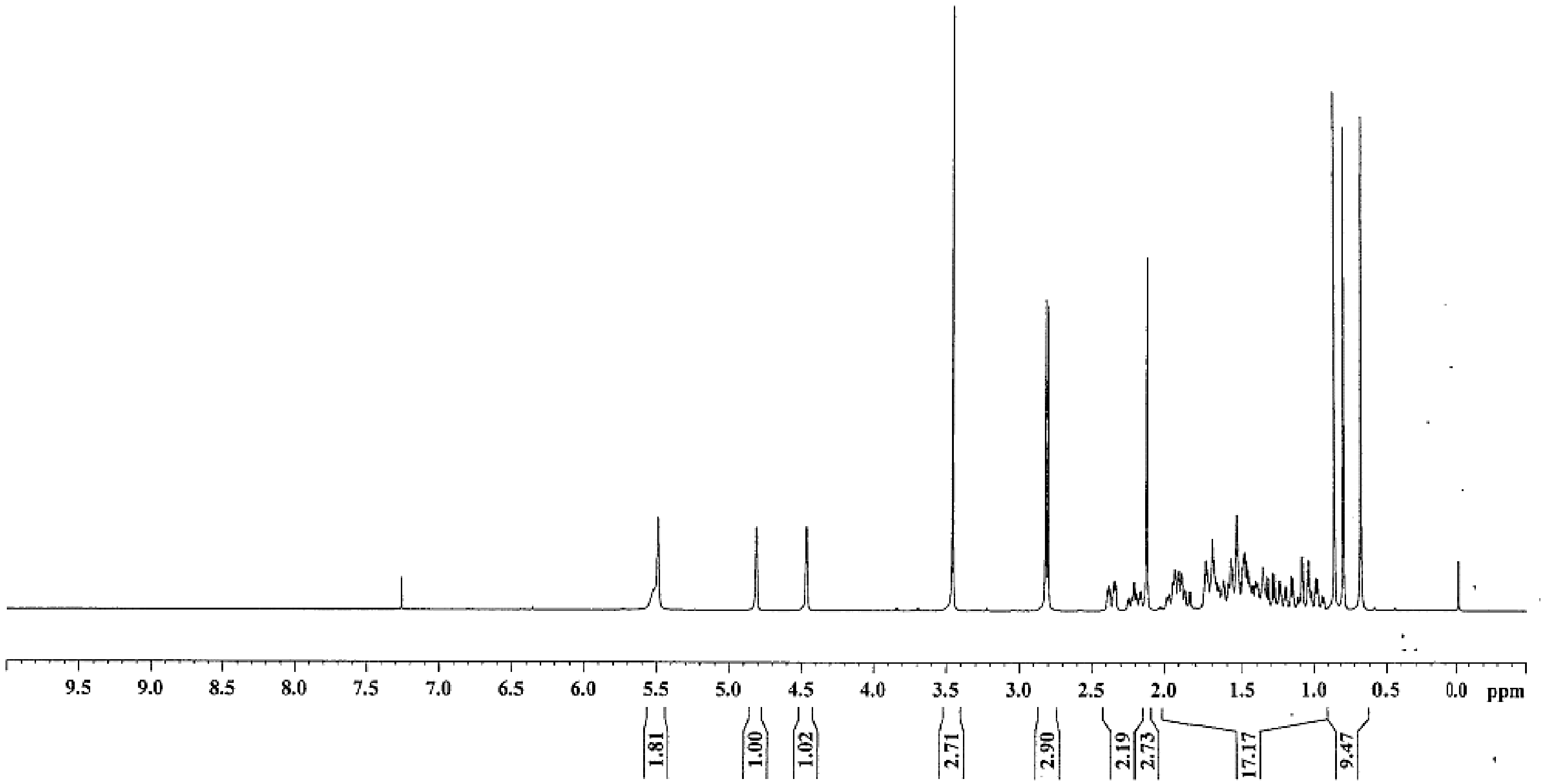

^{13}C NMR of compound **4a** (100 MHz, $CDCl_3$)

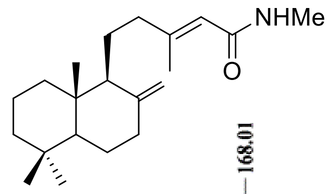

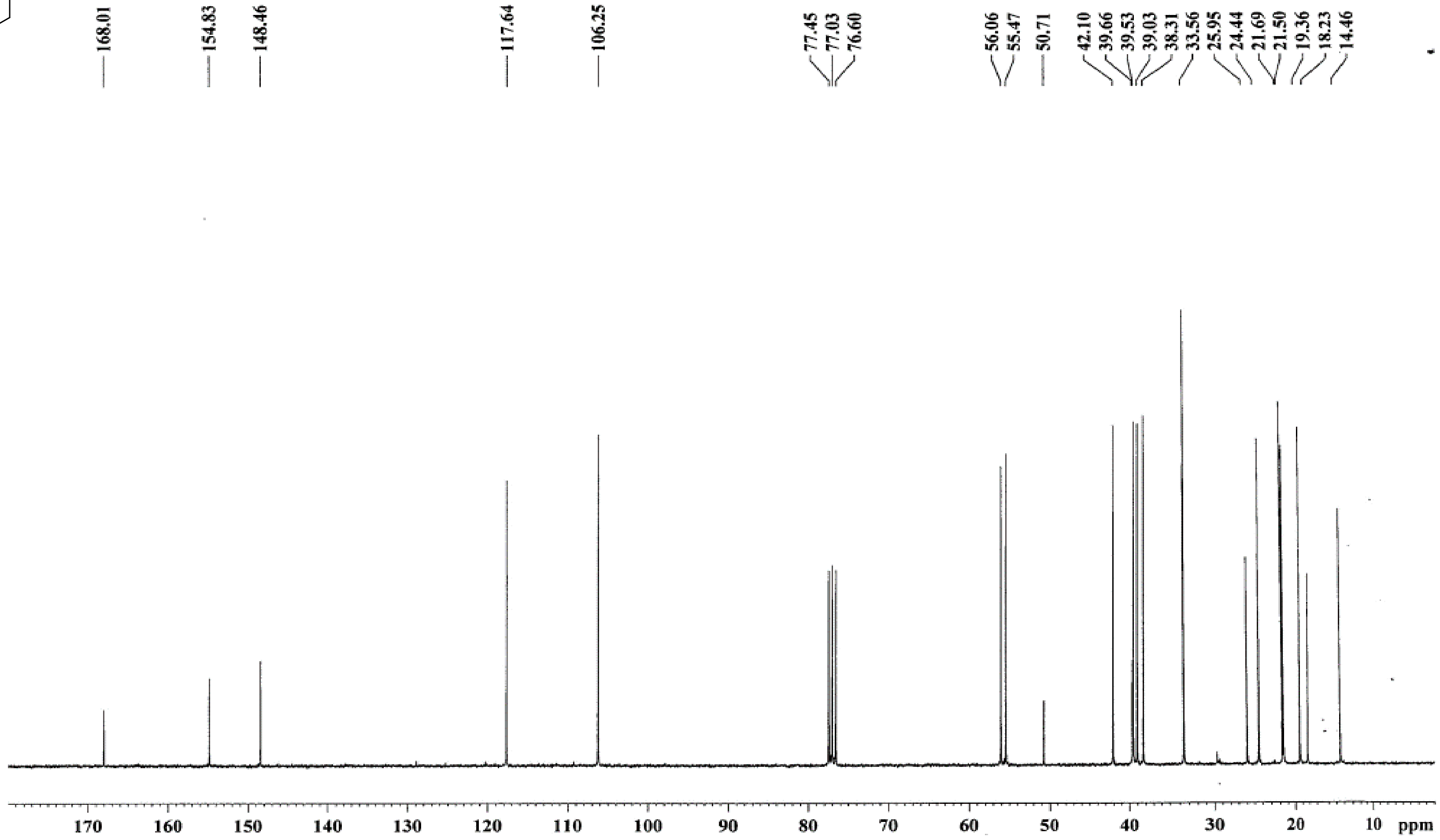

Mass spectrum of compound **4a**

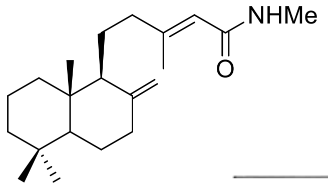

Acquisition Parameter

Source Type	ESI	Ion Polarity	Positive	Set Nebulizer	0.8 Bar
Focus	Not active	Set Capillary	3500 V	Set Dry Heater	100 °C
Scan Begin	100 m/z	Set End Plate Offset	-500 V	Set Dry Gas	5.6 l/min
Scan End	750 m/z	Set Charging Voltage	2000 V	Set Divert Valve	Source
		Set Corona	0 nA	Set APCI Heater	0 °C

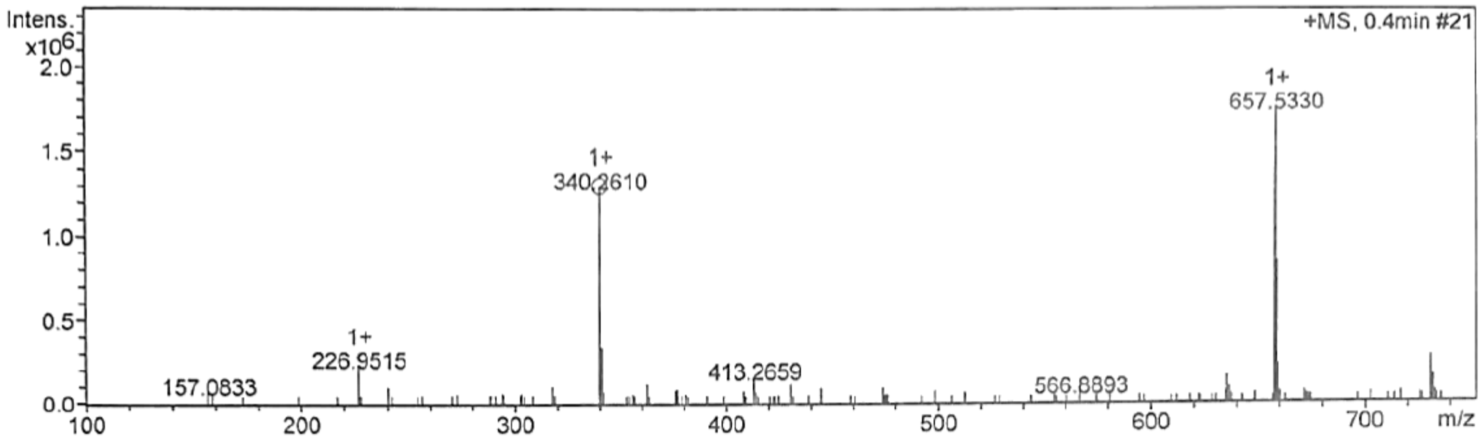

Meas. m/z	#	Ion Formula	Score	m/z	err [mDa]	err [ppm]	mSigma	rdb	e¯ Conf	N-Rule	Adduct
340.261026	1	C21H35NNaO	100.00	340.261085	0.1	0.2	0.8	4.5	even	ok	M+H

^{1}H NMR of compound **4b** (400 MHz, $CDCl_3$)

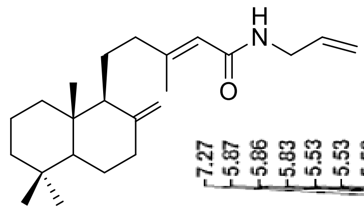

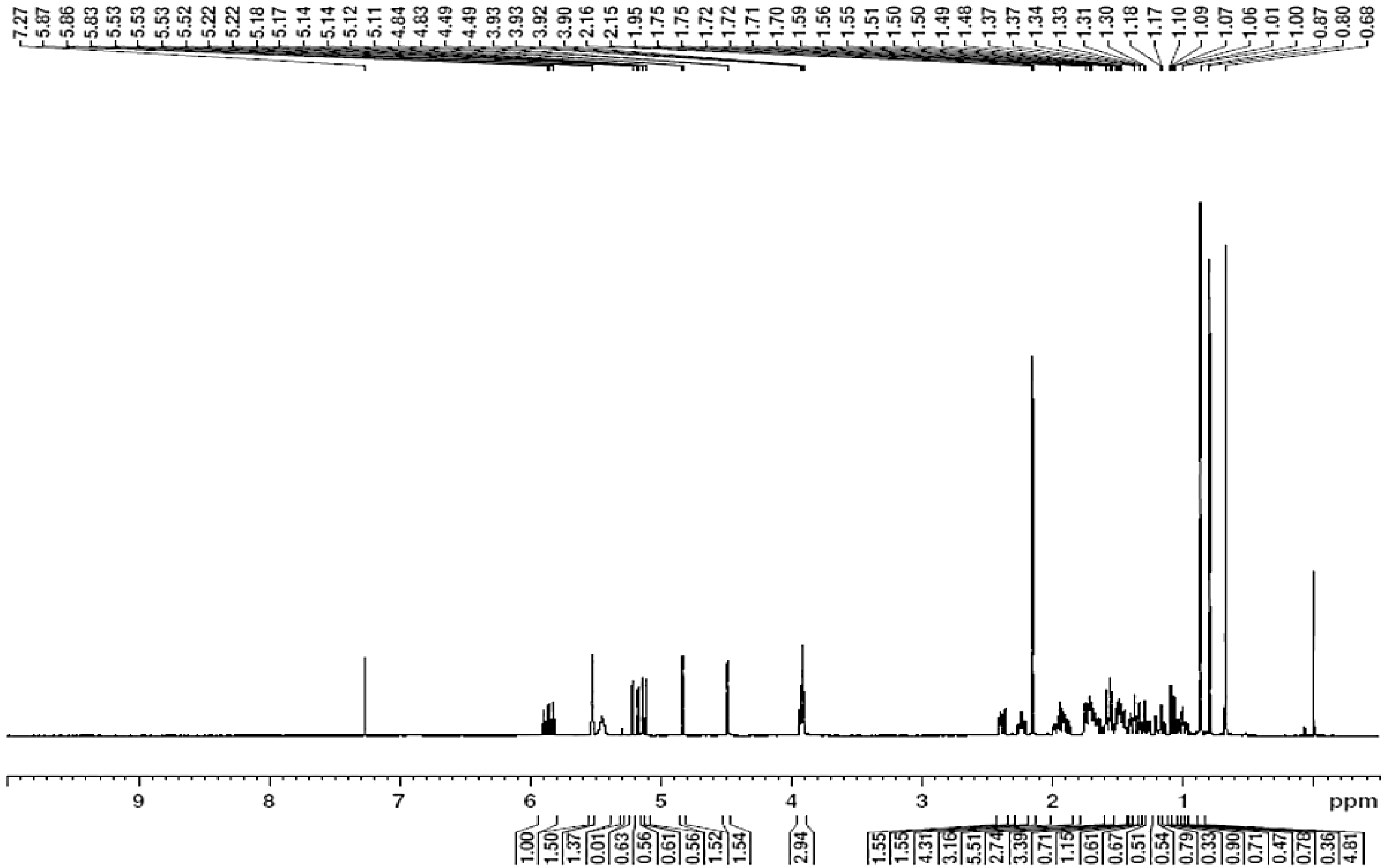

^{13}C NMR of compound **4b** (100 MHz, $CDCl_3$)

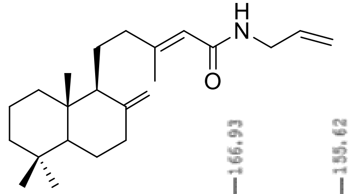

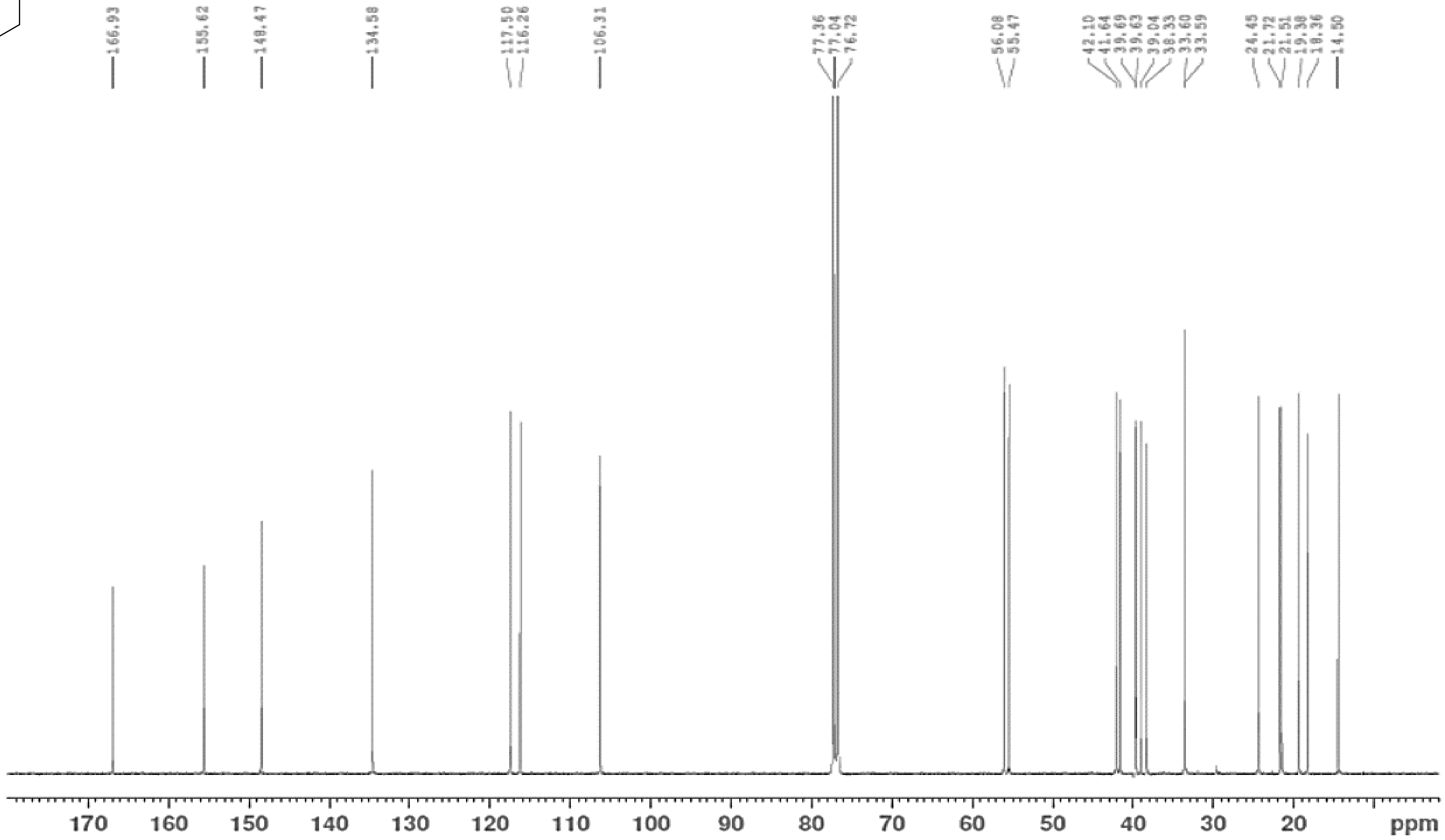

Mass spectrum of compound **4b**

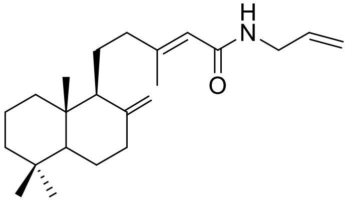

Acquisition Parameter

Source Type	ESI	Ion Polarity	Positive	Set Nebulizer	0.5 Bar
Focus	Not active	Set Capillary	4500 V	Set Dry Heater	89 °C
Scan Begin	50 m/z	Set End Plate Offset	-500 V	Set Dry Gas	6.0 l/min
Scan End	800 m/z	Set Charging Voltage	2000 V	Set Divert Valve	Source
		Set Corona	0 nA	Set APCI Heater	0 °C

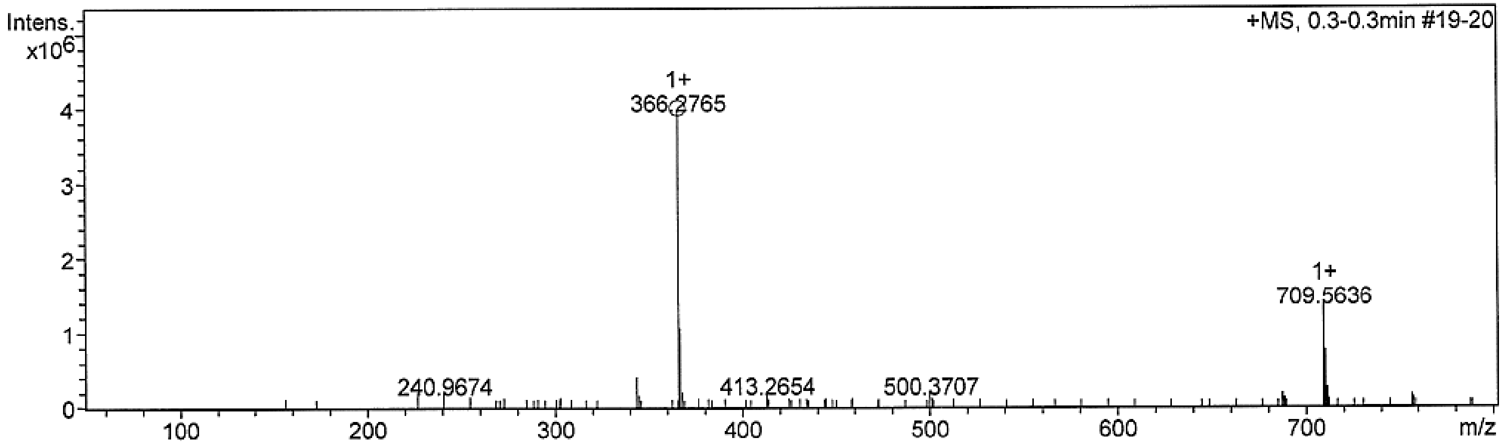

Meas. m/z	#	Ion Formula	Score	m/z	err [mDa]	err [ppm]	mSigma	rdb	e¯ Conf	N-Rule	Adduct
366.276547	1	C23H37NNaO	100.00	366.276736	0.2	0.5	4.1	5.5	even	ok	M+H

^{1}H NMR of compound **4c** (400 MHz, $CDCl_3$)

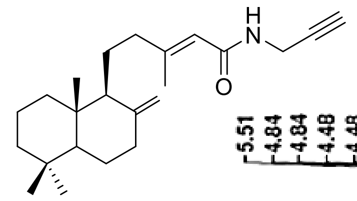

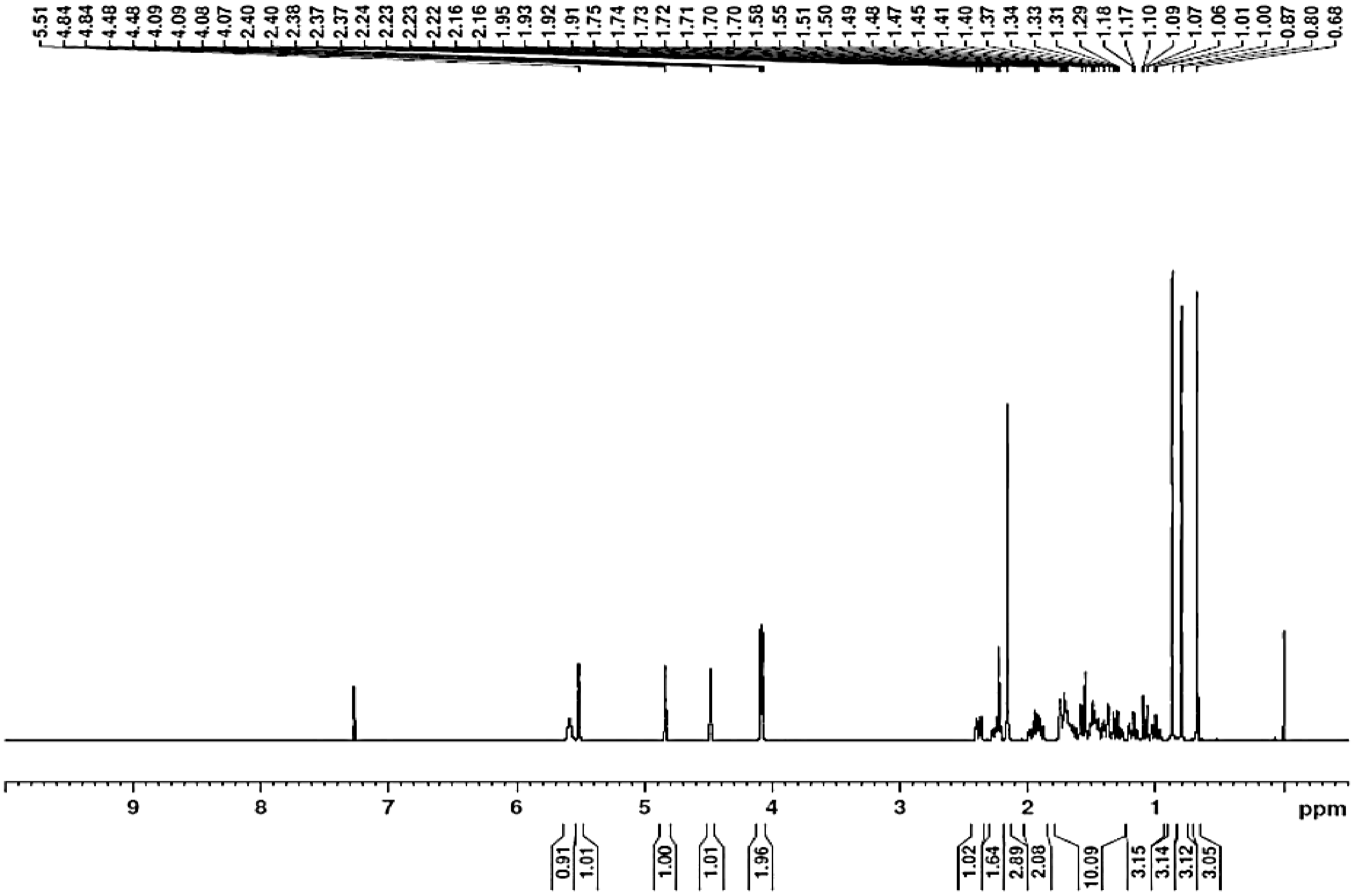

^{13}C NMR of compound **4c** (100 MHz, $CDCl_3$)

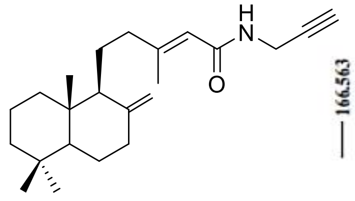

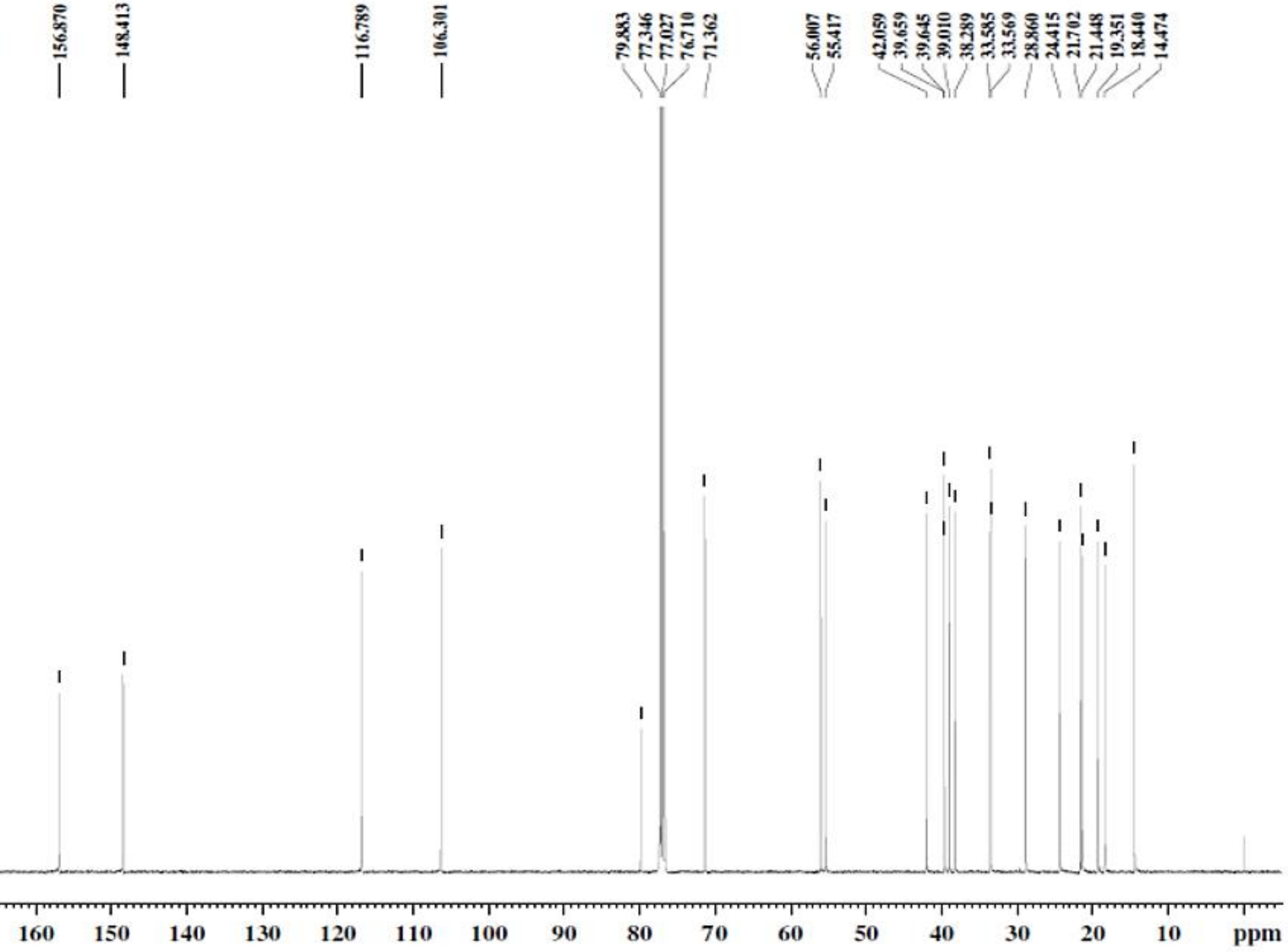

Mass spectrum of compound **4c**

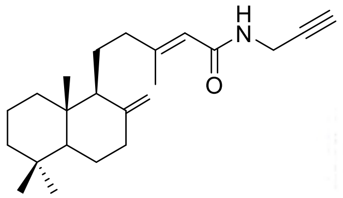

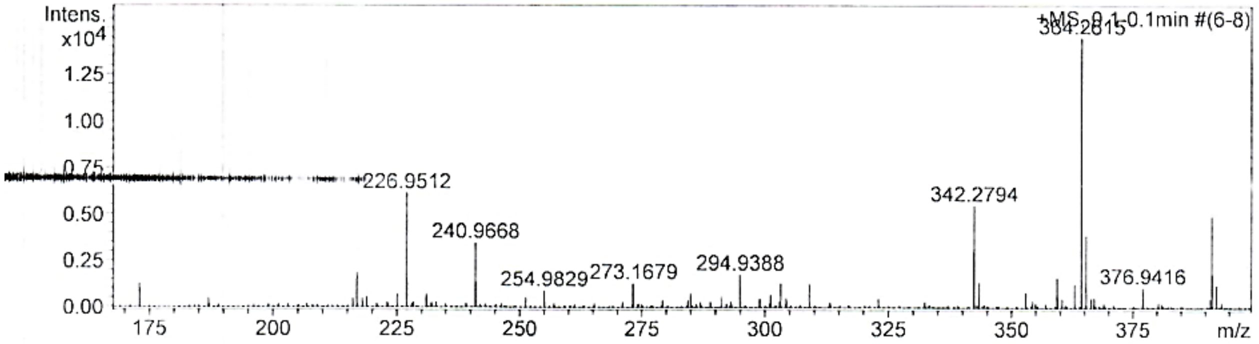

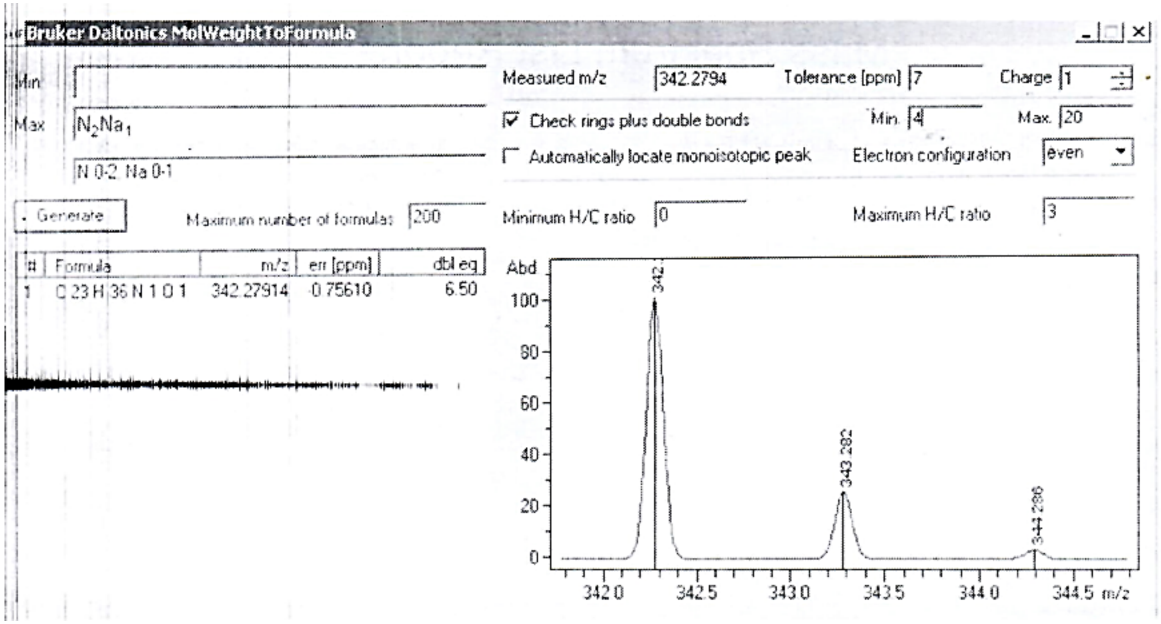

^{1}H NMR of compound **4d** (400 MHz, $CDCl_3$)

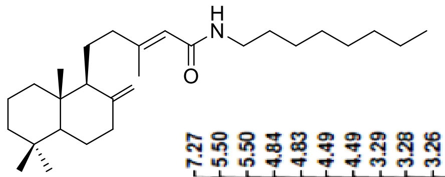

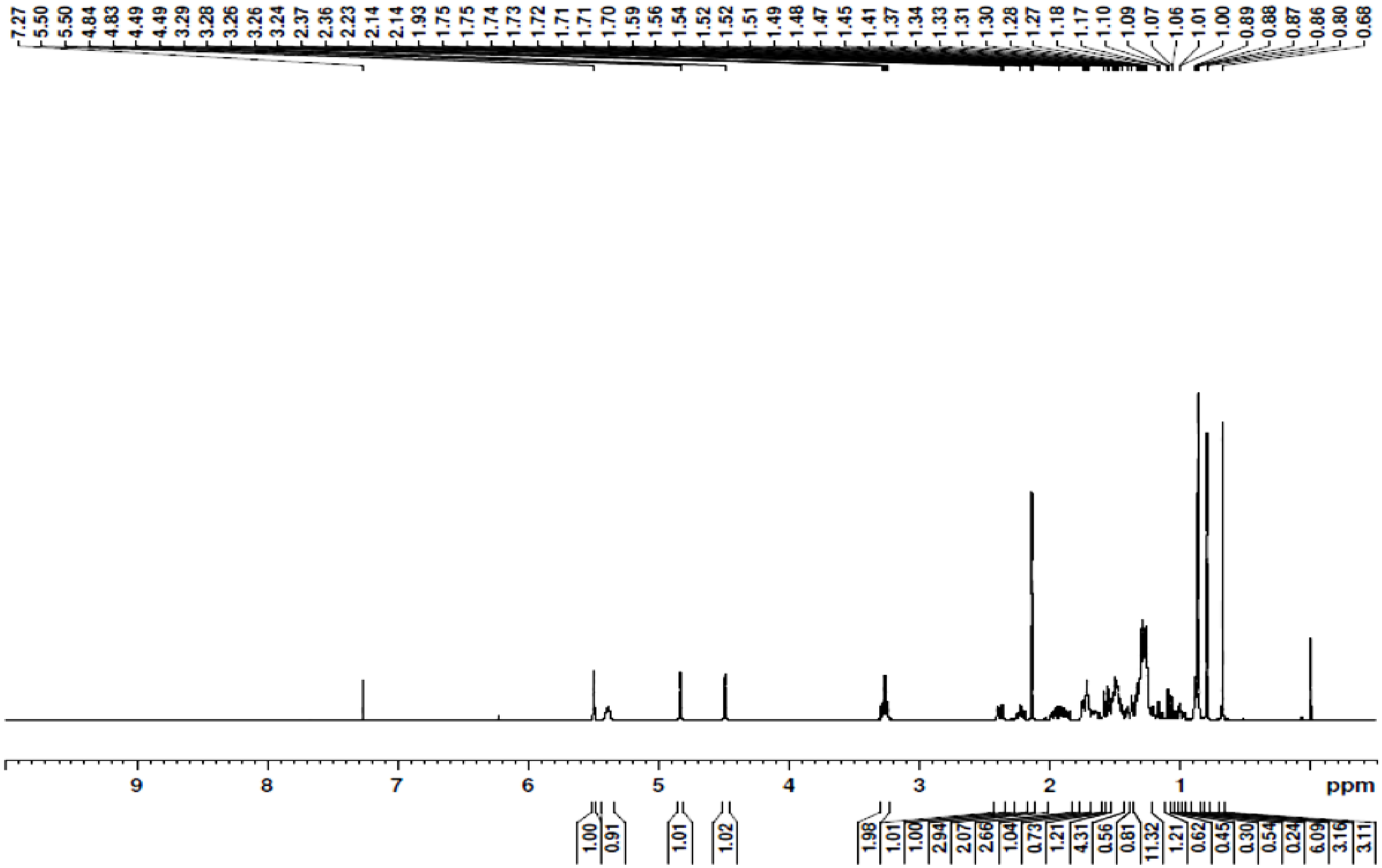

^{13}C NMR of compound **4d** (100 MHz, $CDCl_3$)

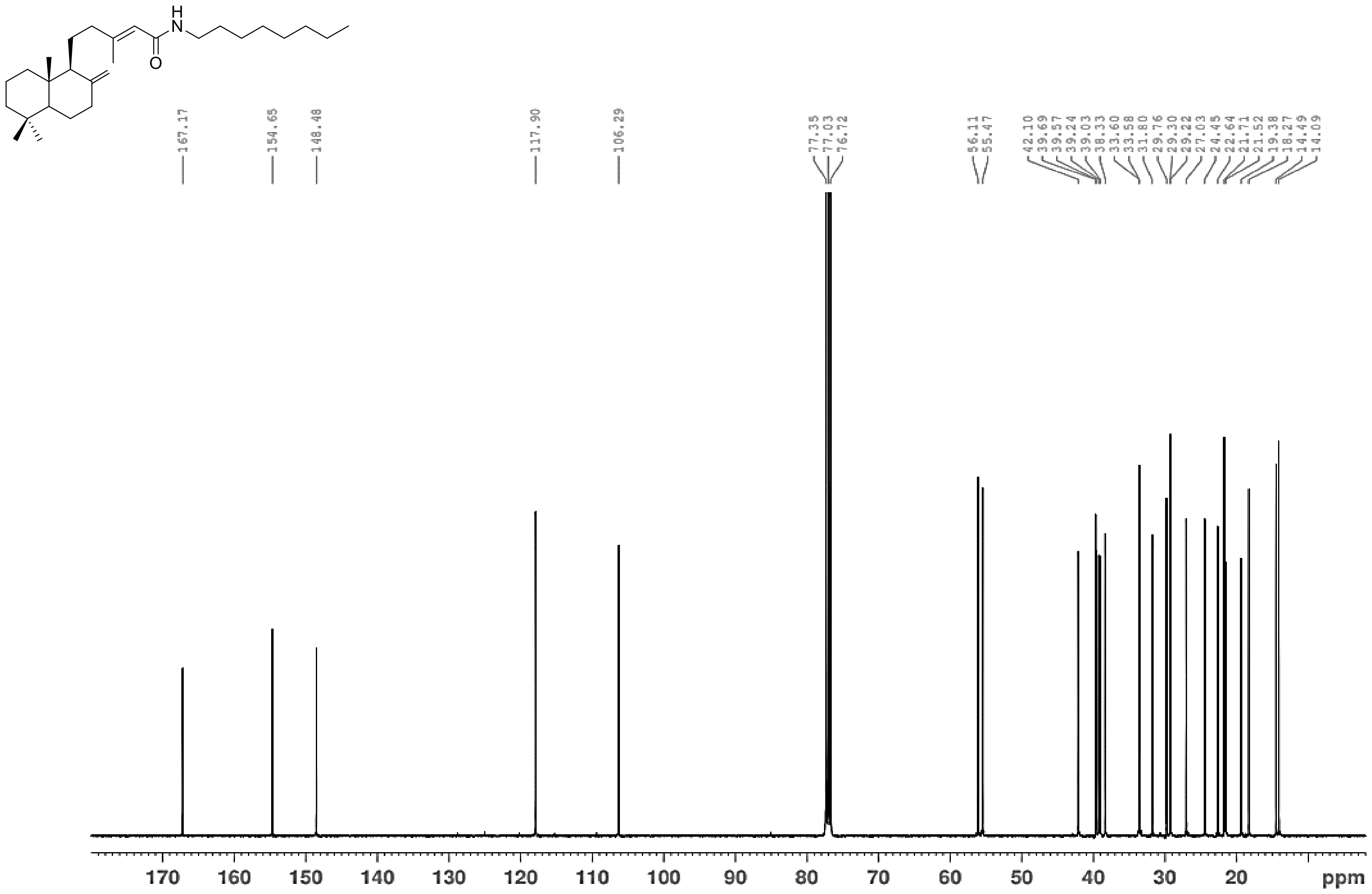

Mass spectrum of compound **4d**

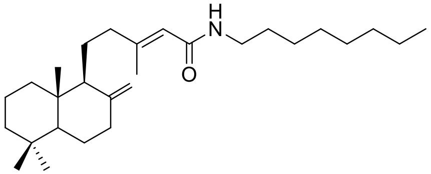

Analysis Info

Analysis Name	D:\Data\CRI\QCR00706 Pornsuda Copa-56-C1 E+.d	Acquisition Date	1/17/2017 2:20:53 PM
Method	Nitirat esi pos low may2016.m	Operator	BDAL@DE
Sample Name	ESIpos	Instrument	compact 8255754.20094
Comment			

Acquisition Parameter

Source Type	ESI	Ion Polarity	Positive	Set Nebulizer	0.5 Bar
Focus	Not active	Set Capillary	4500 V	Set Dry Heater	89 °C
Scan Begin	50 m/z	Set End Plate Offset	-500 V	Set Dry Gas	6.0 l/min
Scan End	800 m/z	Set Charging Voltage	2000 V	Set Divert Valve	Source
		Set Corona	0 nA	Set APCI Heater	0 °C

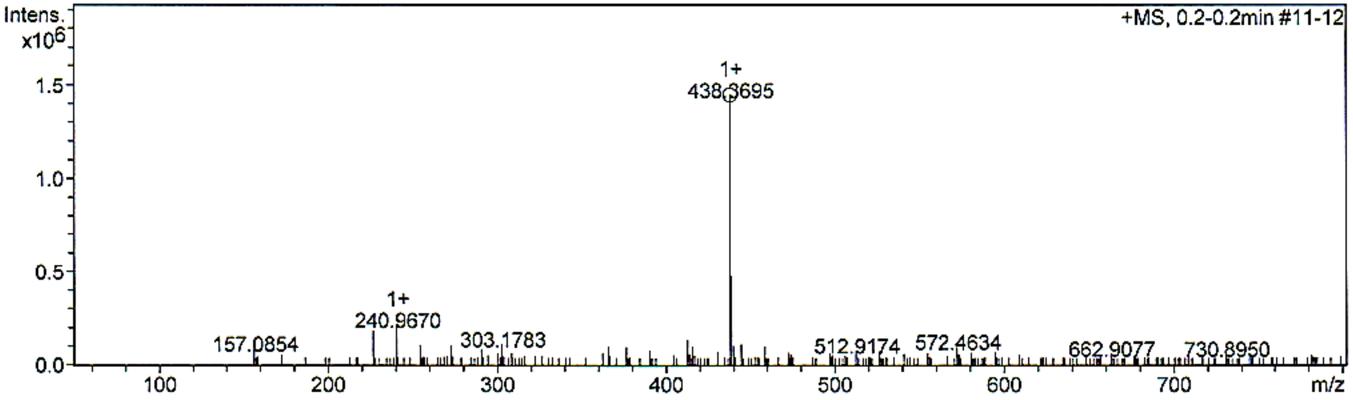

Meas. m/z	#	Ion Formula	Score	m/z	err [mDa]	err [ppm]	mSigma	rdb	e⁻ Conf	N-Rule	Adduct
438.369456	1	C28H49NNaO	100.00	438.370636	1.2	2.7	1.3	4.5	even	ok	M+H

^{1}H NMR of compound **4e** (400 MHz, $CDCl_3$)

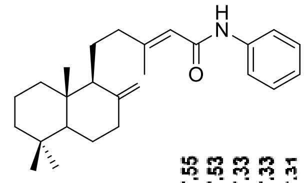

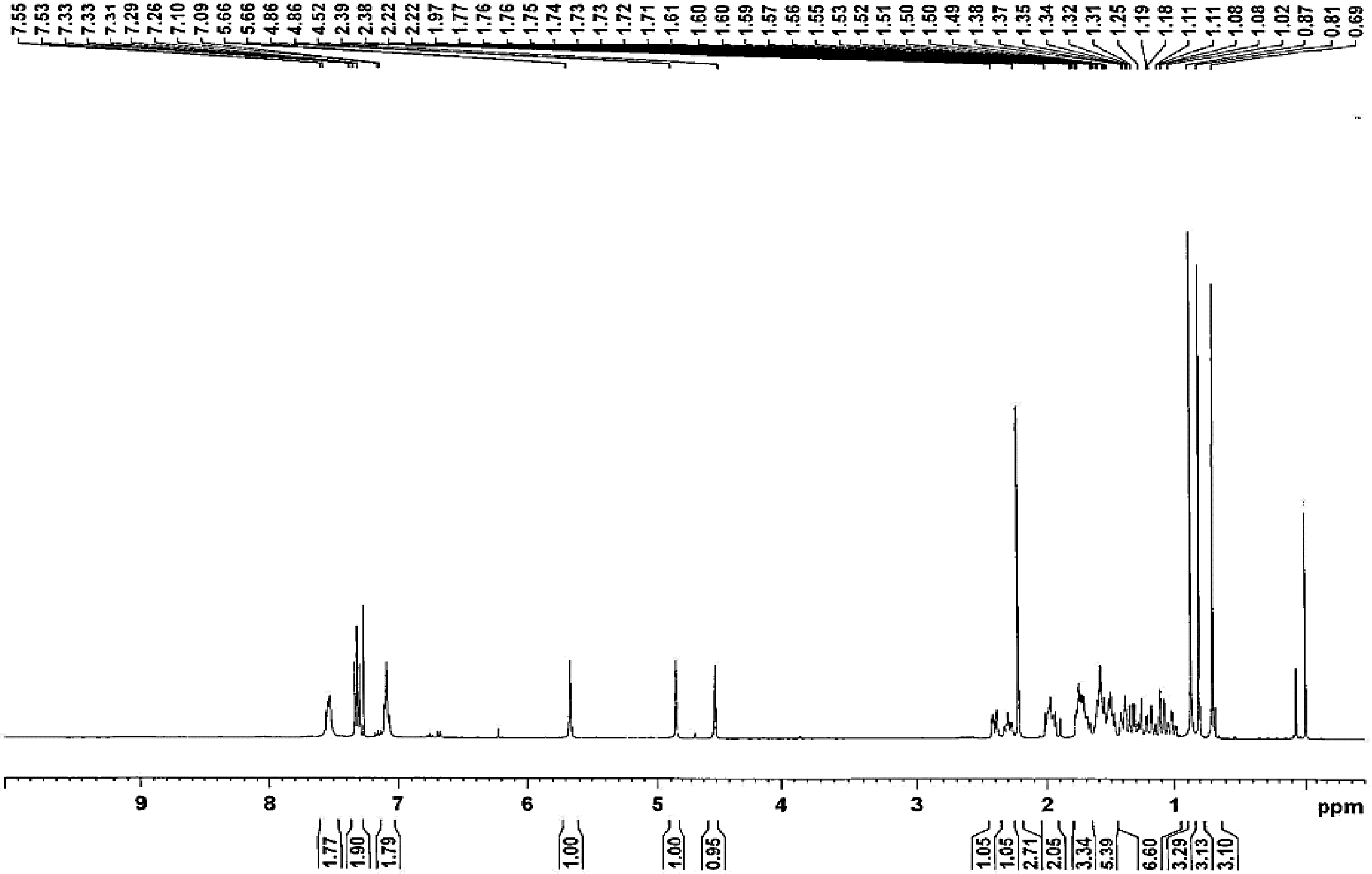

^{13}C NMR of compound **4e** (100 MHz, $CDCl_3$)

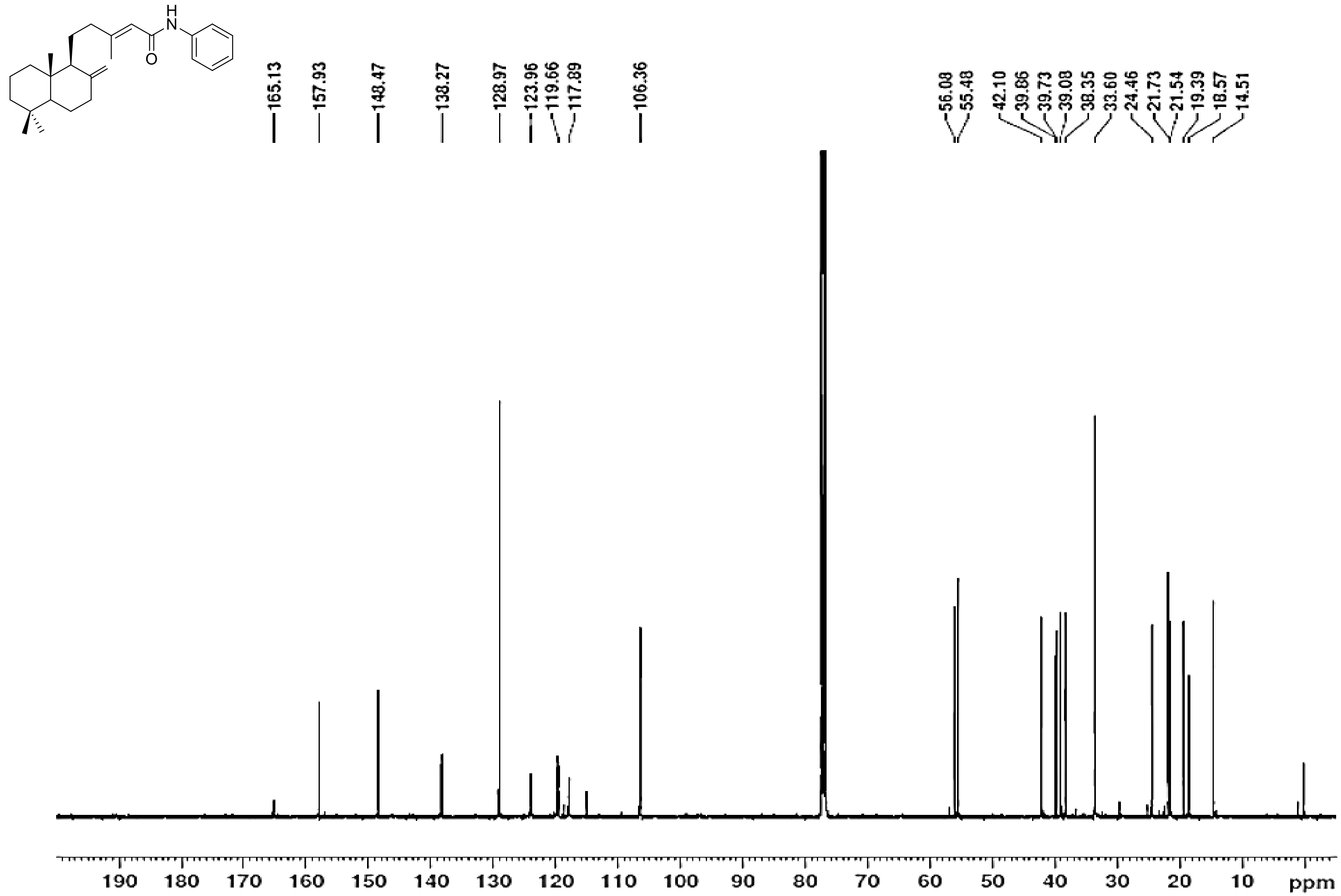

Mass spectrum of compound **4e**

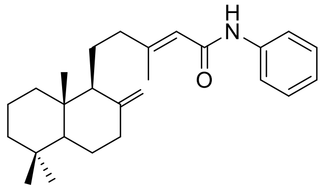

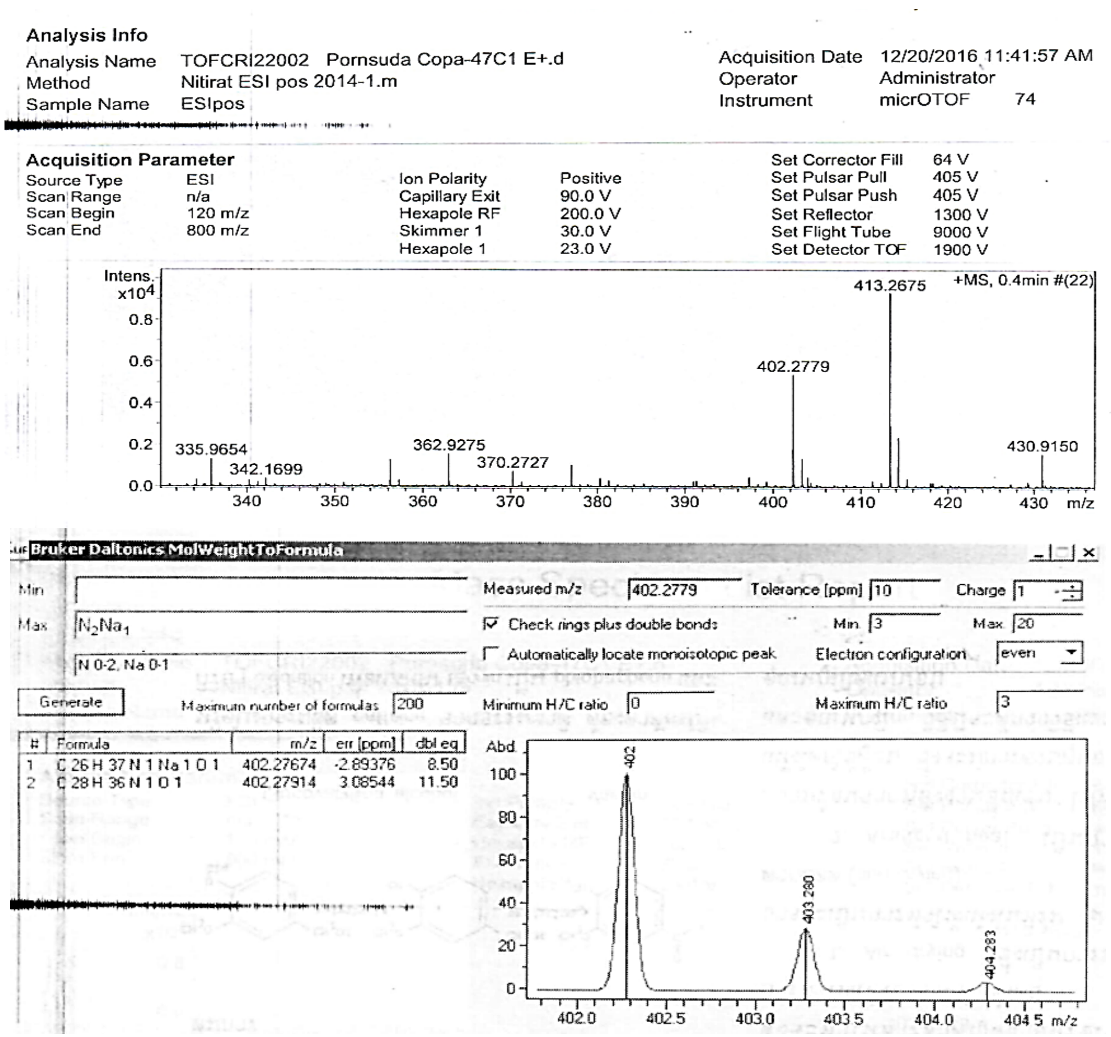

^{1}H NMR of compound **4f** (400 MHz, $CDCl_3$)

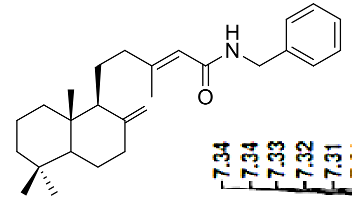

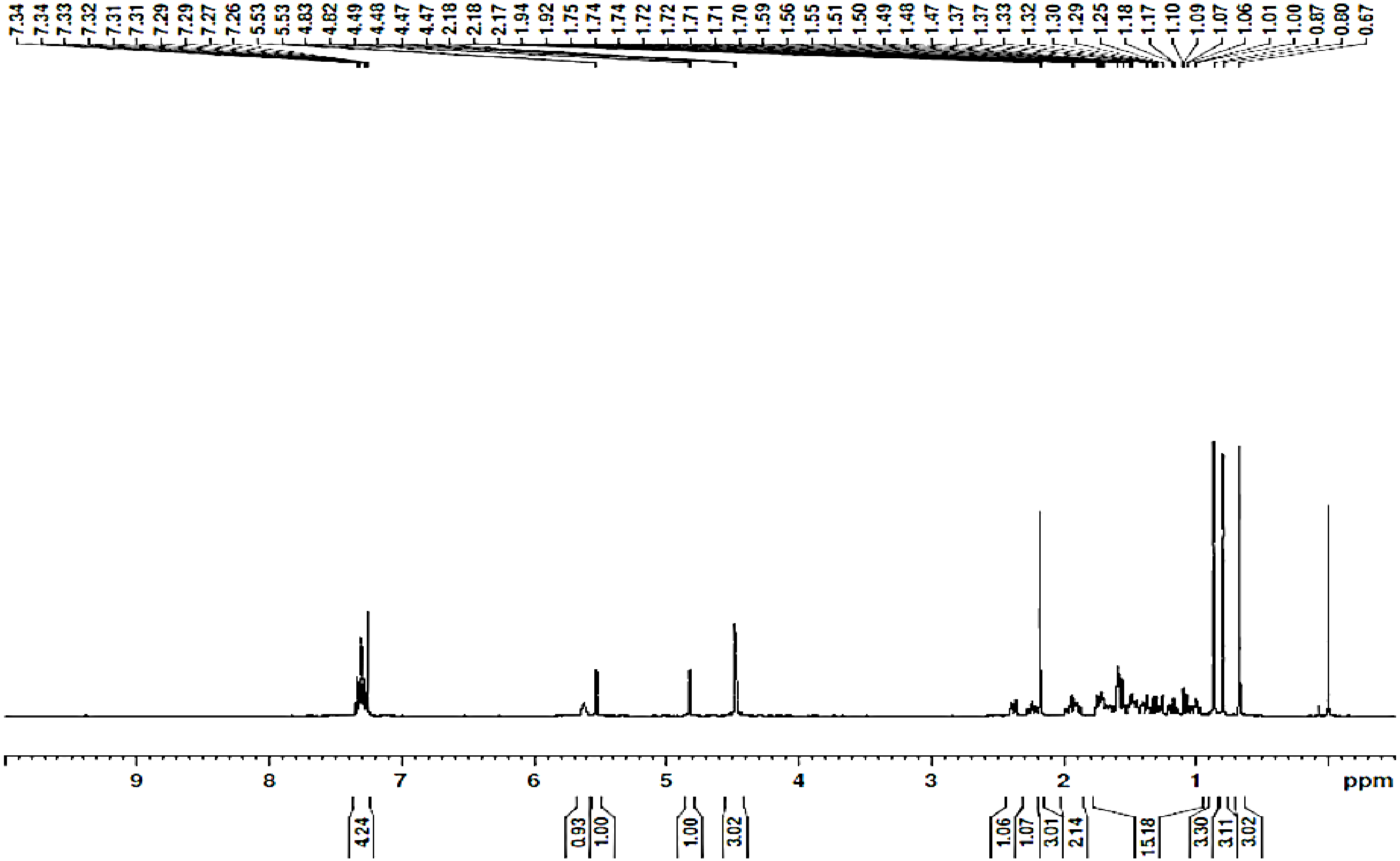

^{13}C NMR of compound **4f** (100 MHz, $CDCl_3$)

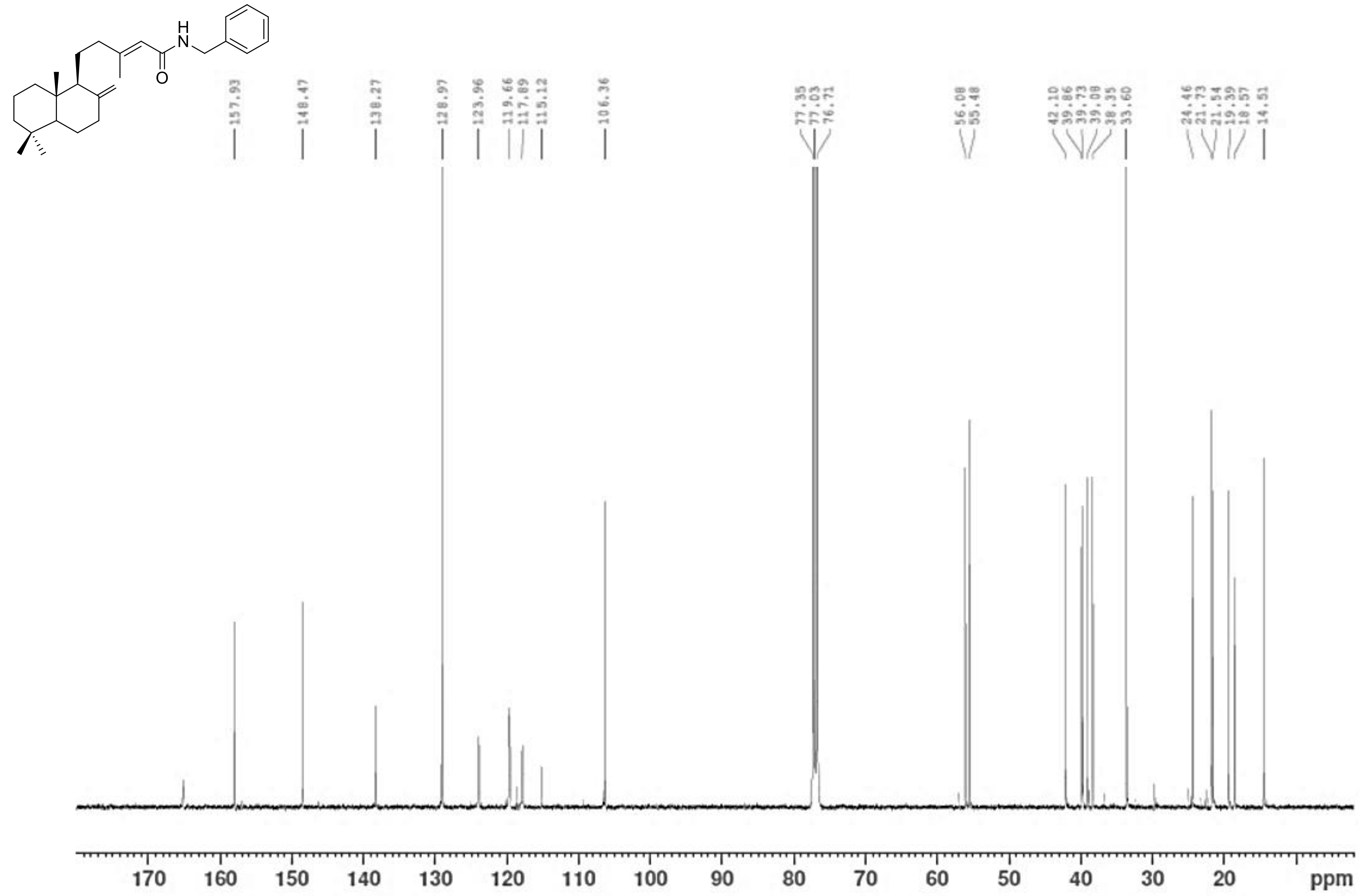

Mass spectrum of compound **4f**

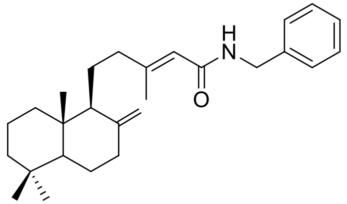

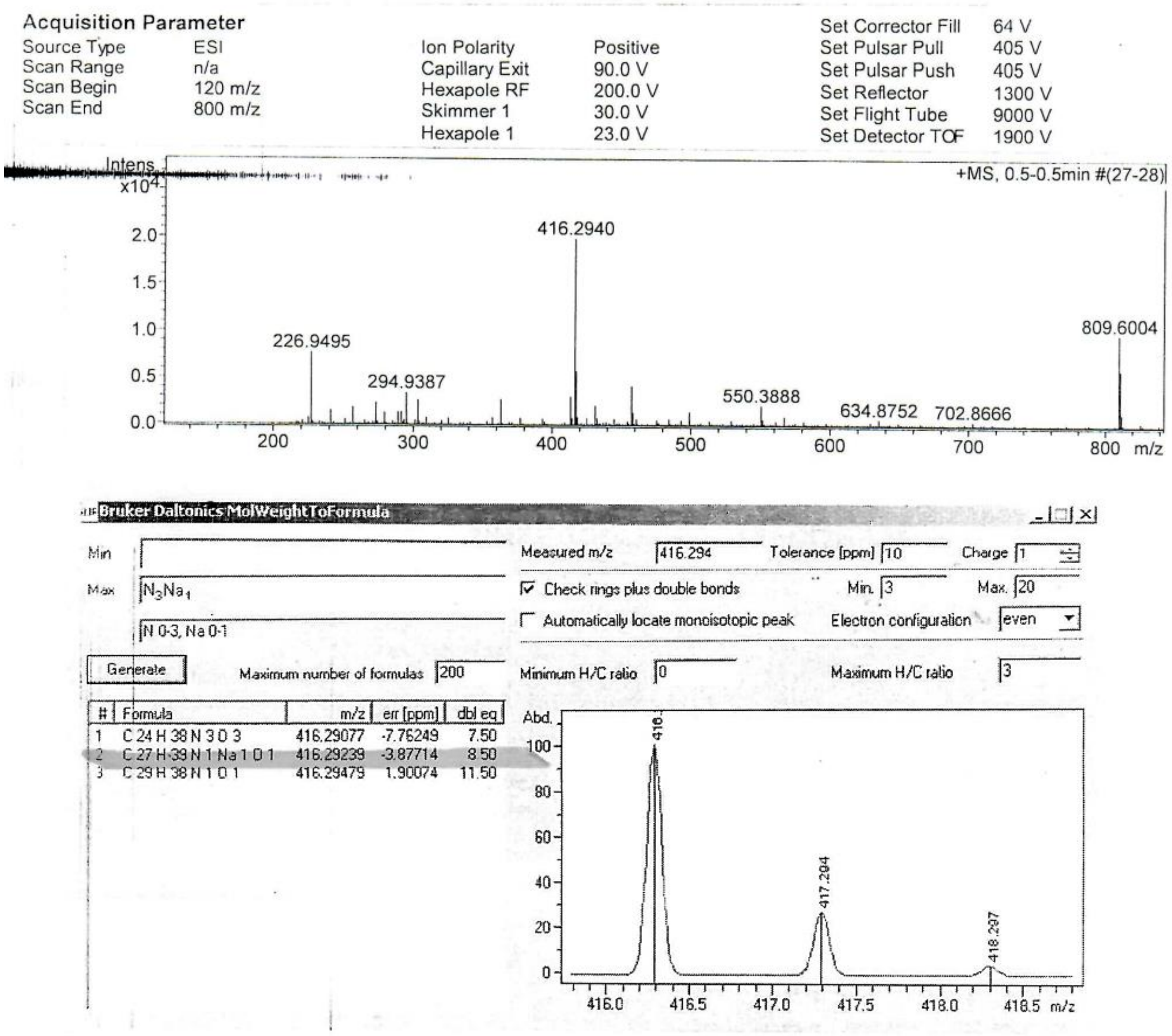

^{1}H NMR of compound **4g** (400 MHz, $CDCl_3$)

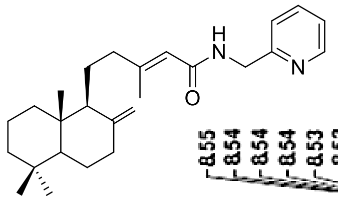

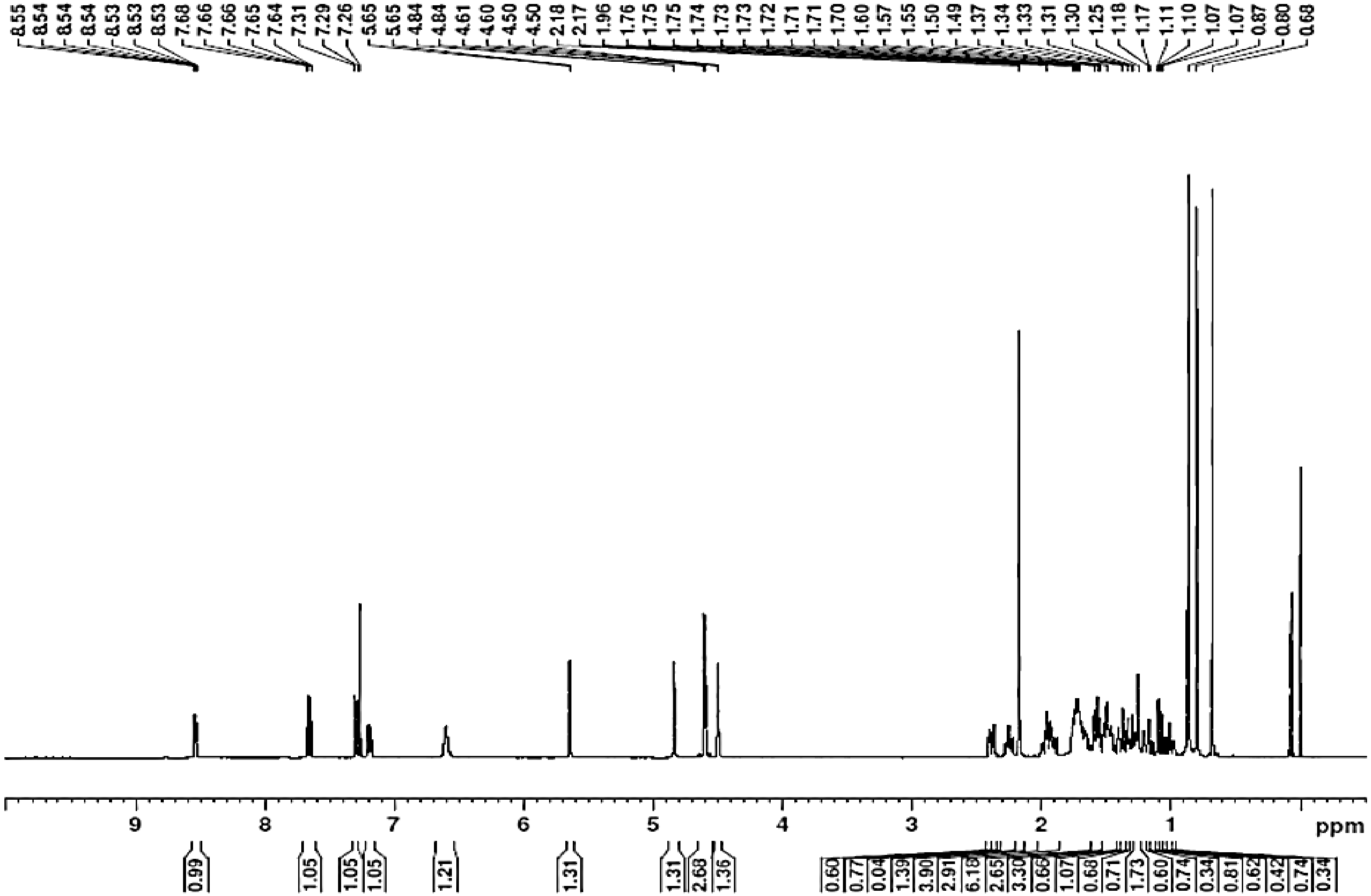

^{13}C NMR of compound **4g** (100 MHz, $CDCl_3$)

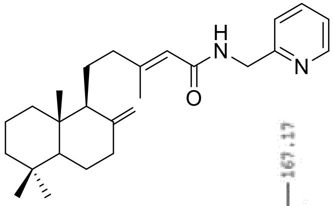

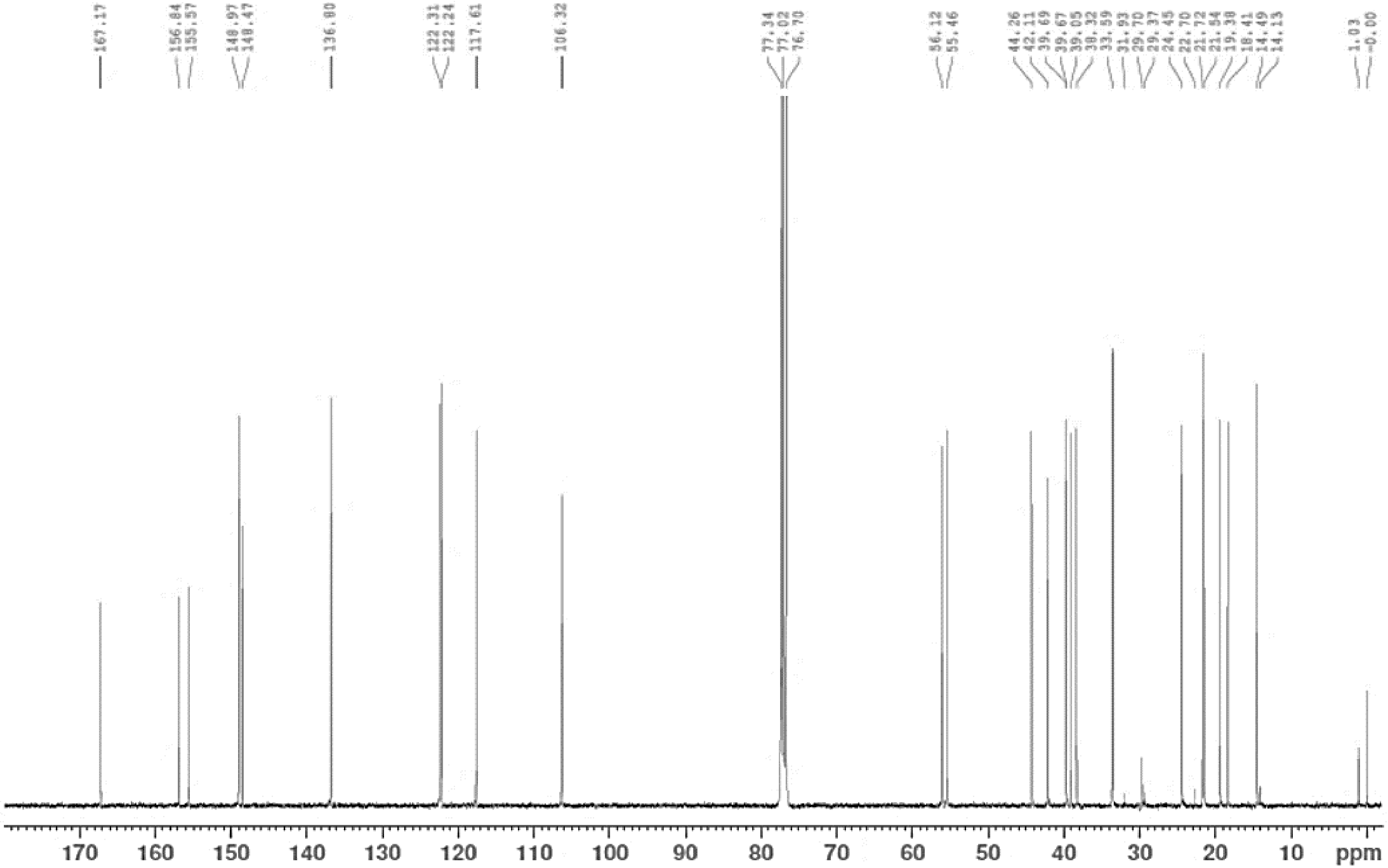

Mass spectrum of compound **4g**

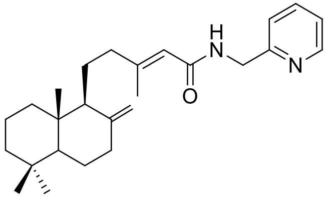

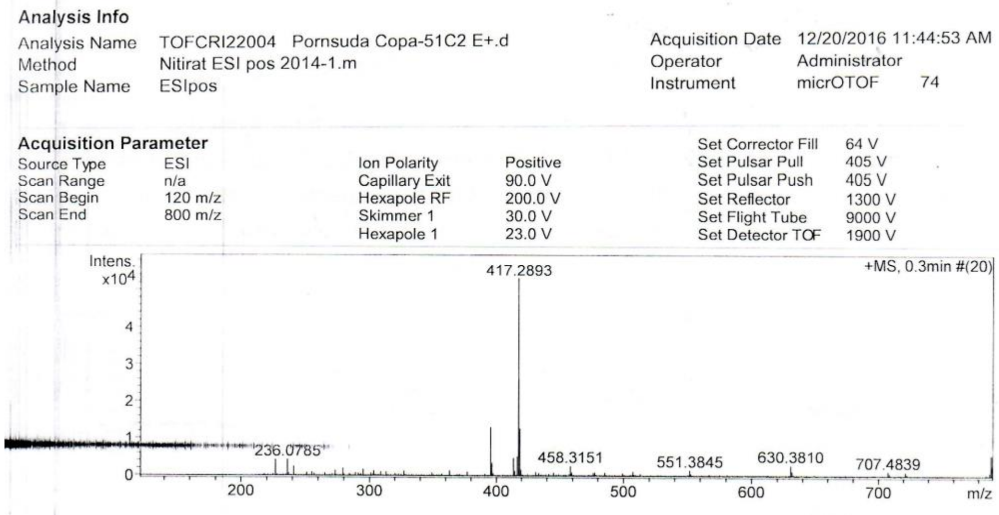

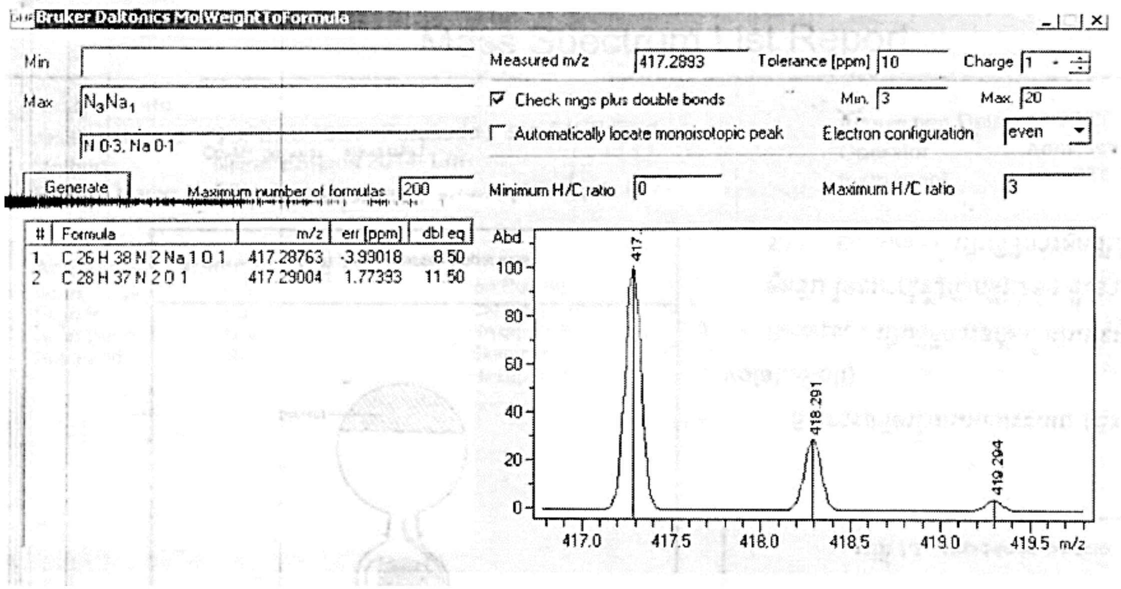

^{1}H NMR of compound **4h** (400 MHz, $CDCl_3$)

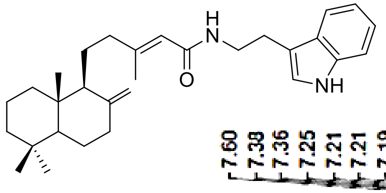

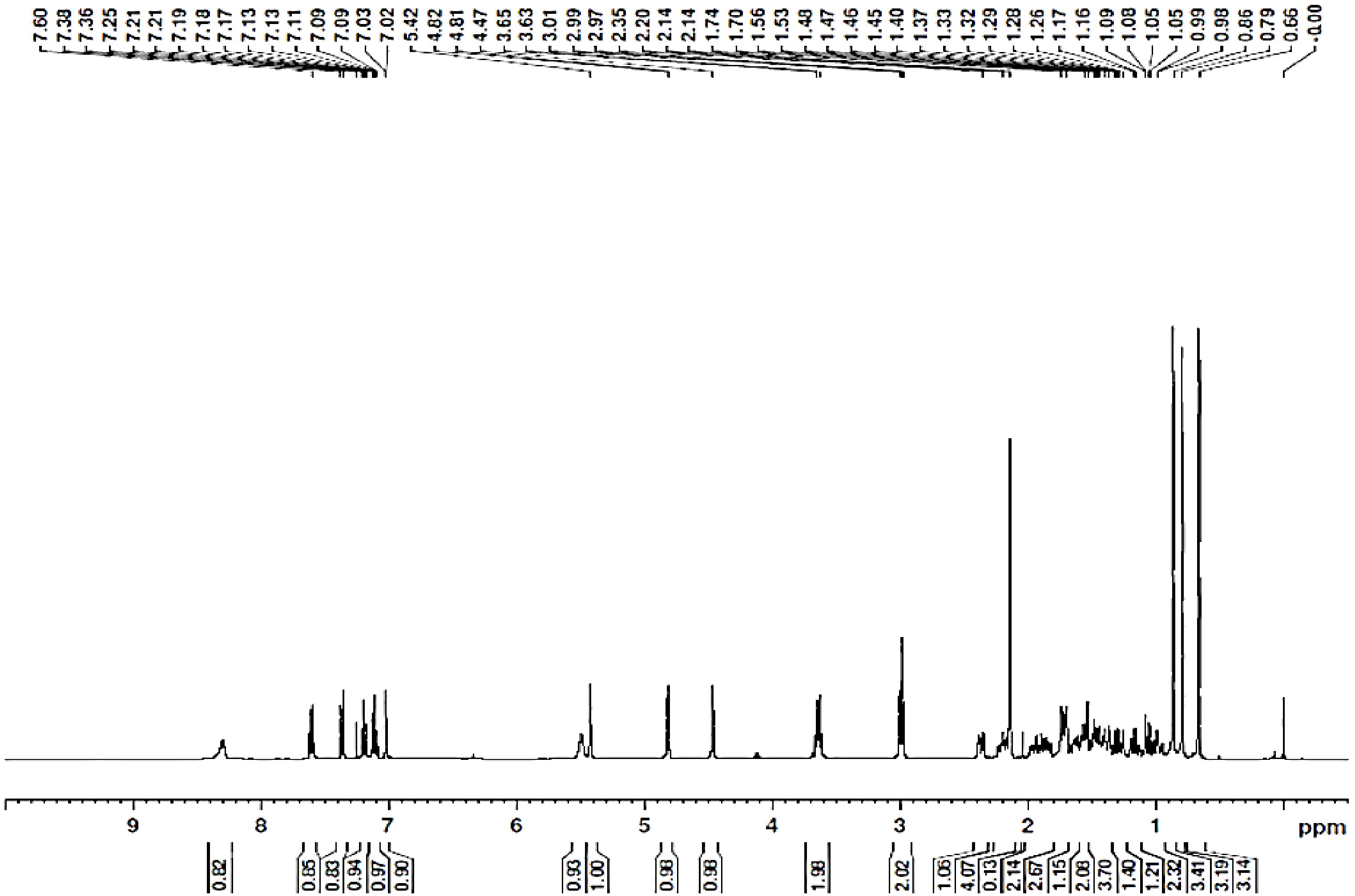

^{13}C NMR of compound **4h** (100 MHz, $CDCl_3$)

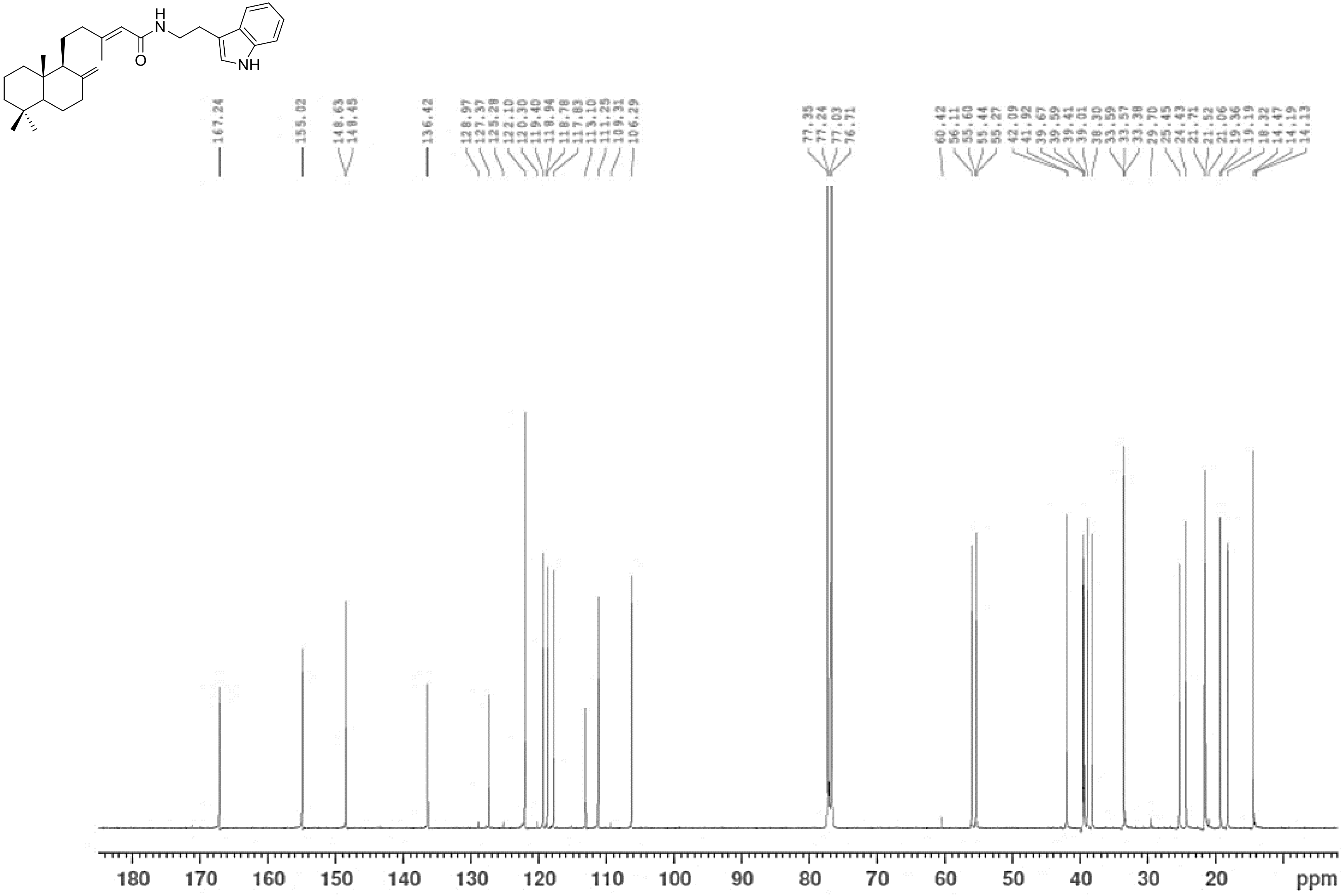

Mass spectrum of compound **4h**

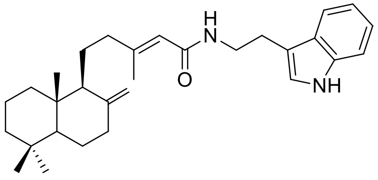

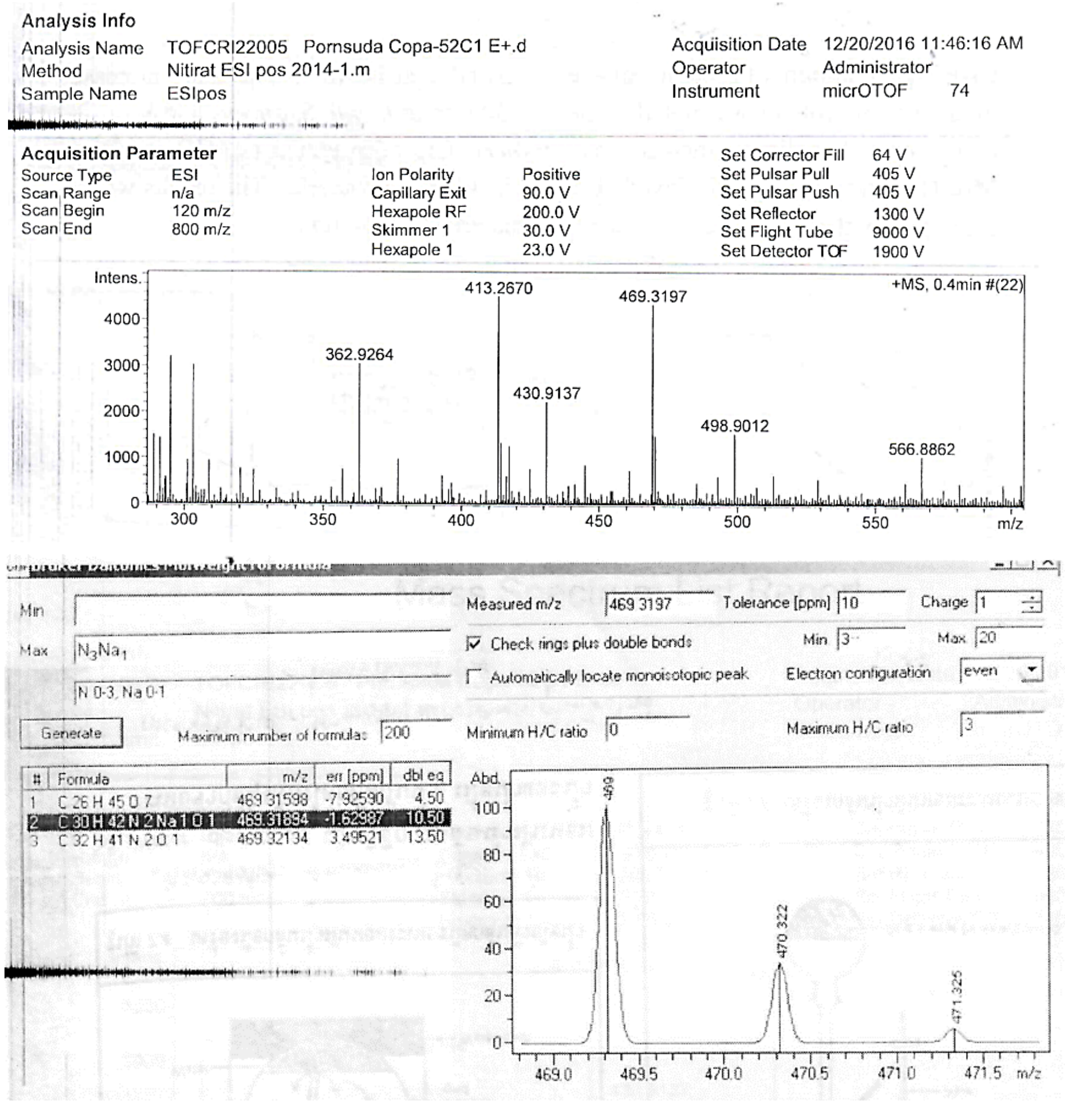

#	Formula	m/z	err [ppm]	dbl eq
1	C 26 H 45 O 7	469.31598	-7.92590	4.50
2	C 30 H 42 N 2 Na 1 O 1	469.31894	-1.62987	10.50
3	C 32 H 41 N 2 O 1	469.32134	3.49521	13.50

^{1}H NMR of compound **4i** (400 MHz, $CDCl_3$)

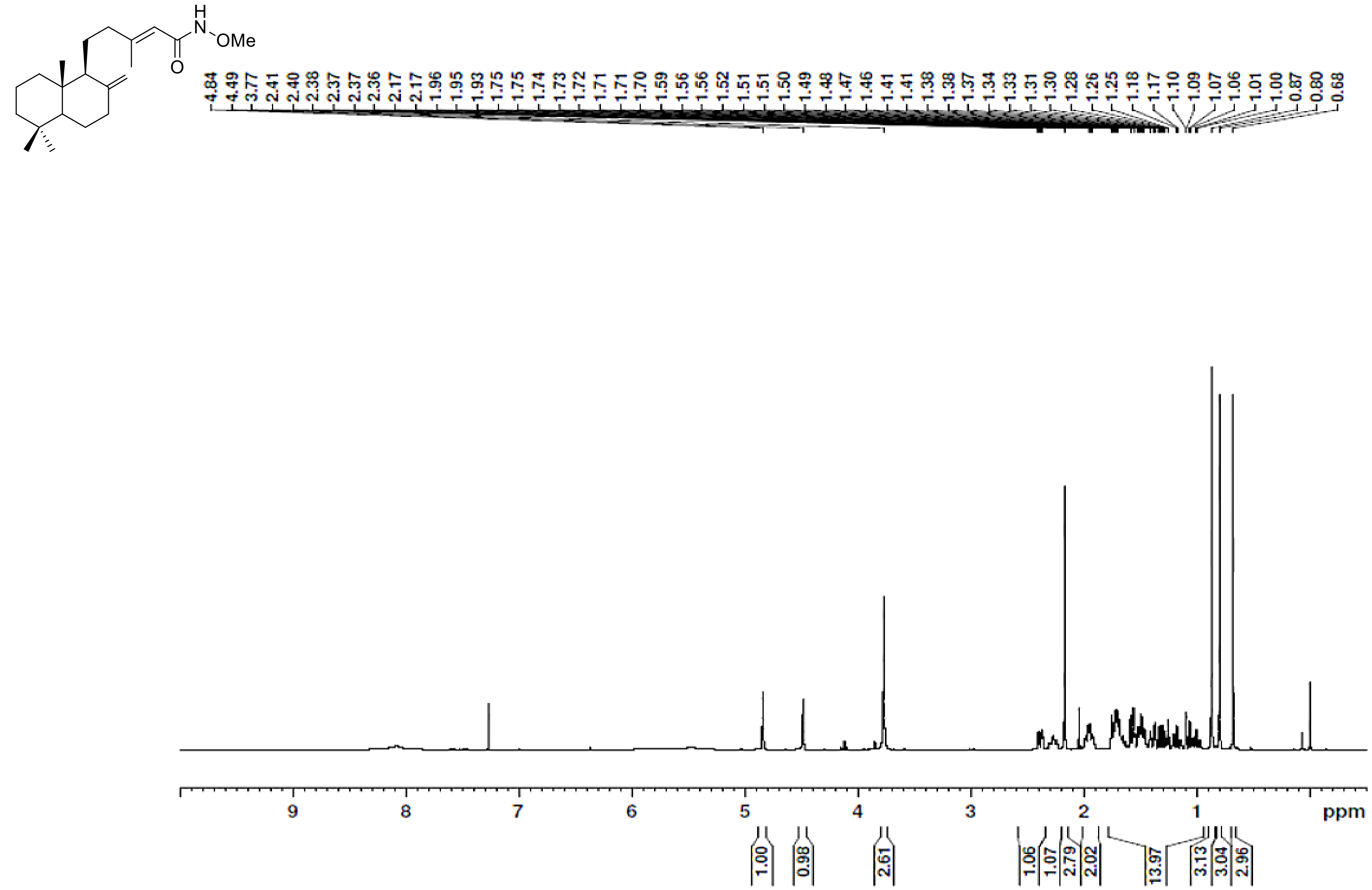

^{13}C NMR of compound **4i** (100 MHz, $CDCl_3$)

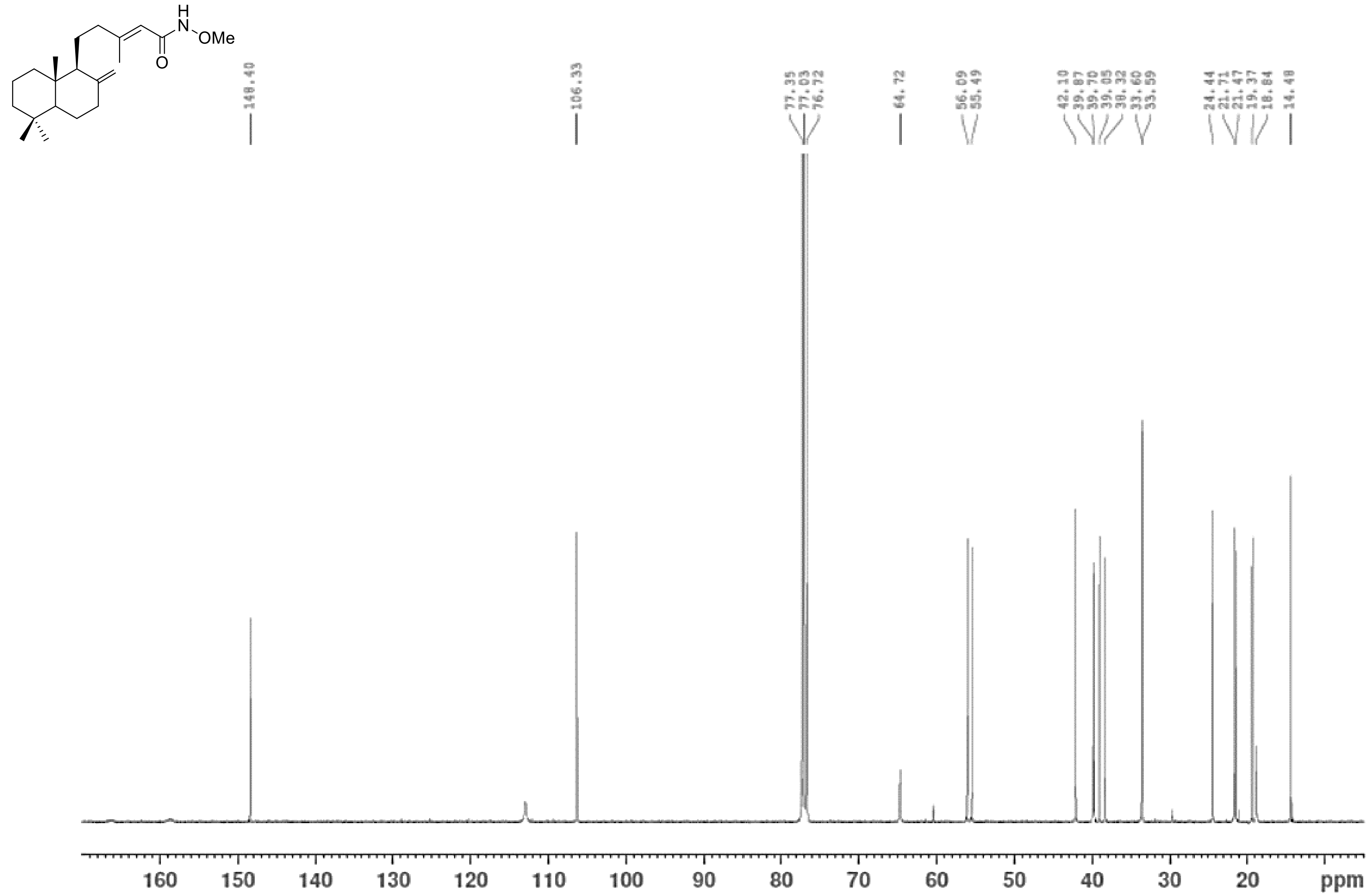

Mass spectrum of compound **4i**

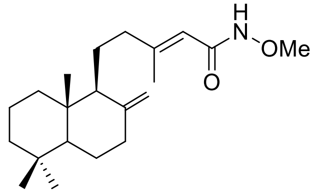

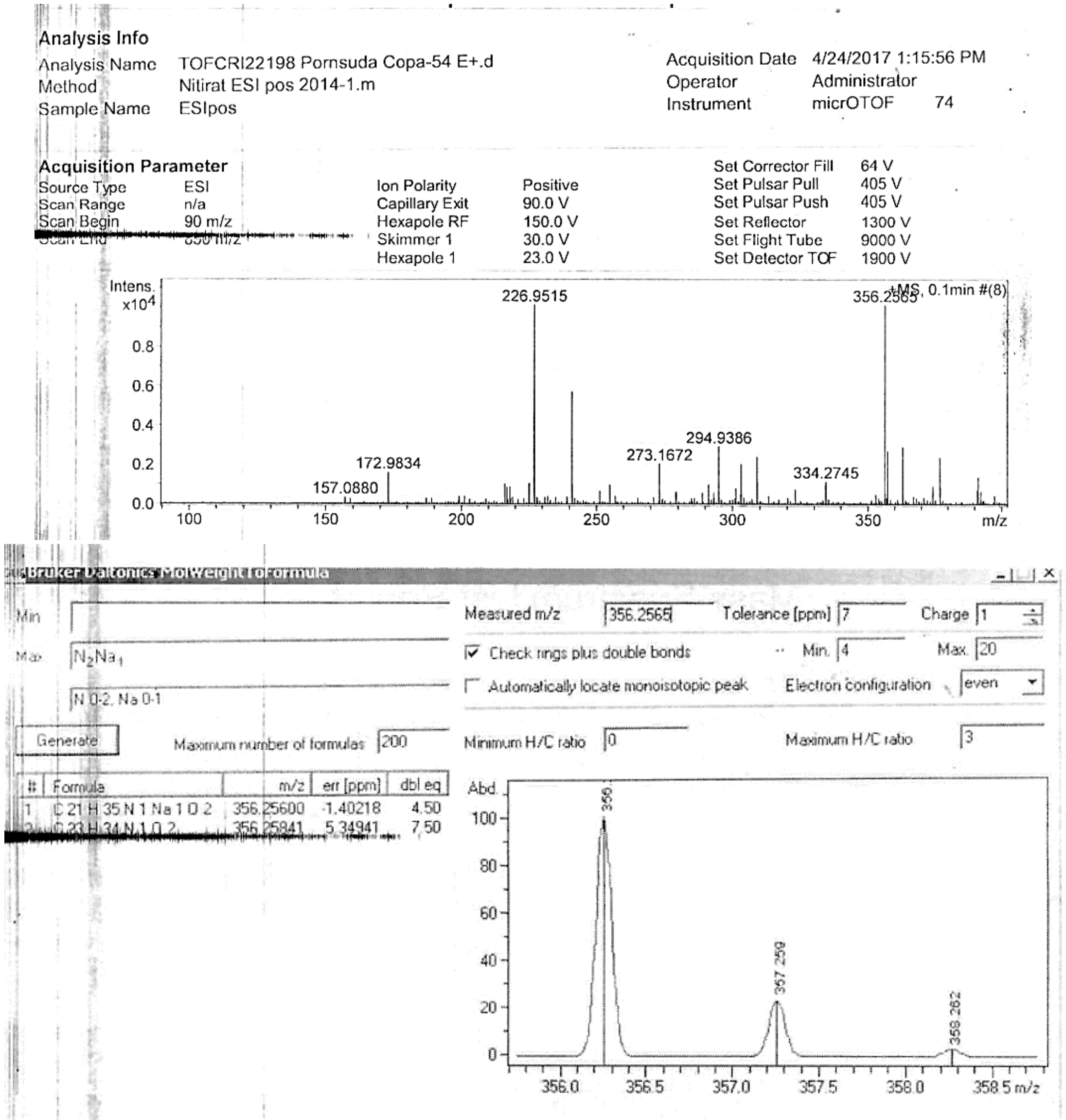

^{1}H NMR of compound **4j** (400 MHz, $CDCl_3$)

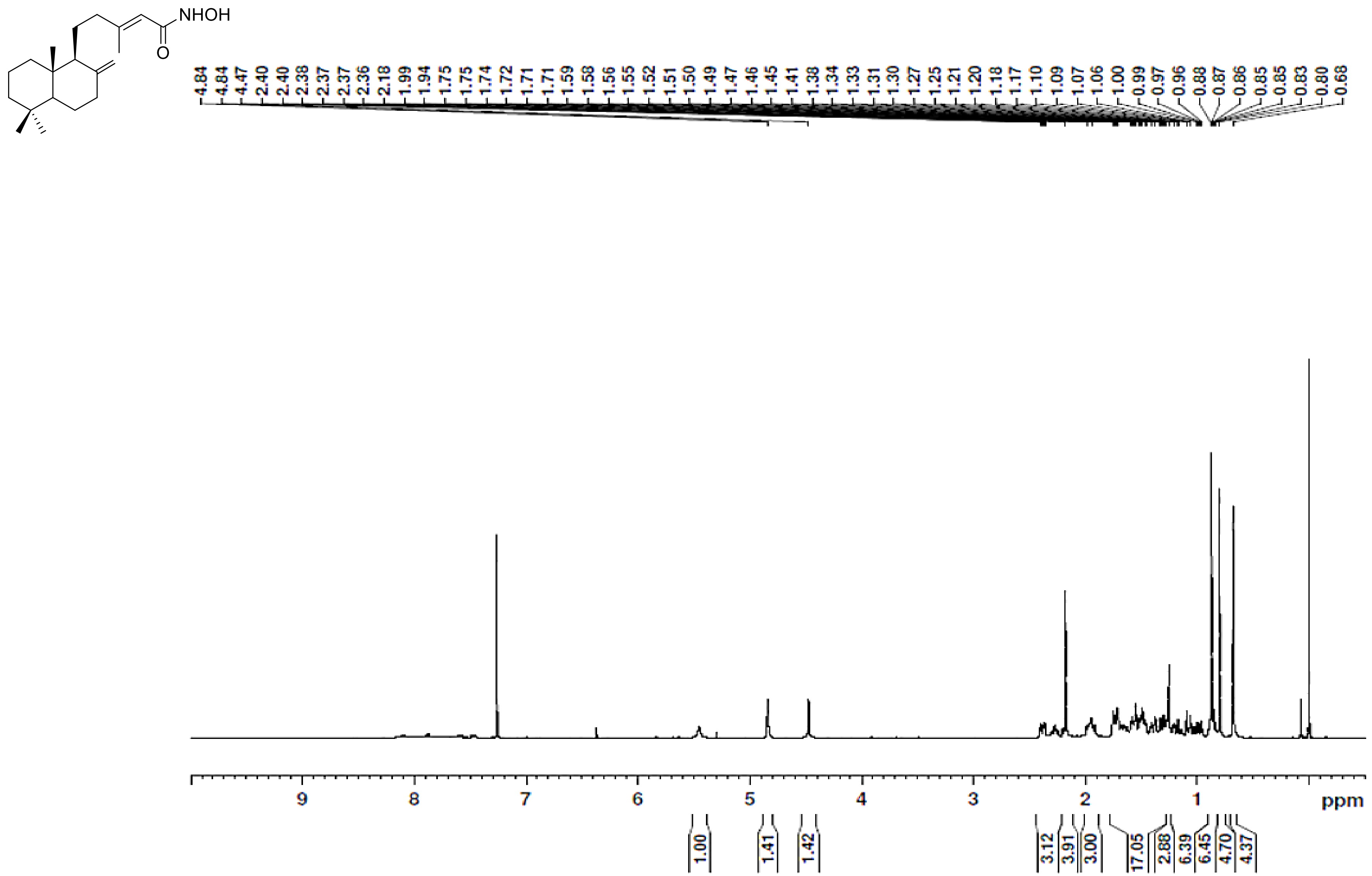

^{13}C NMR of compound **4j** (100 MHz, CDCl$_3$)

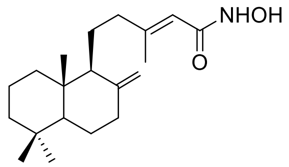

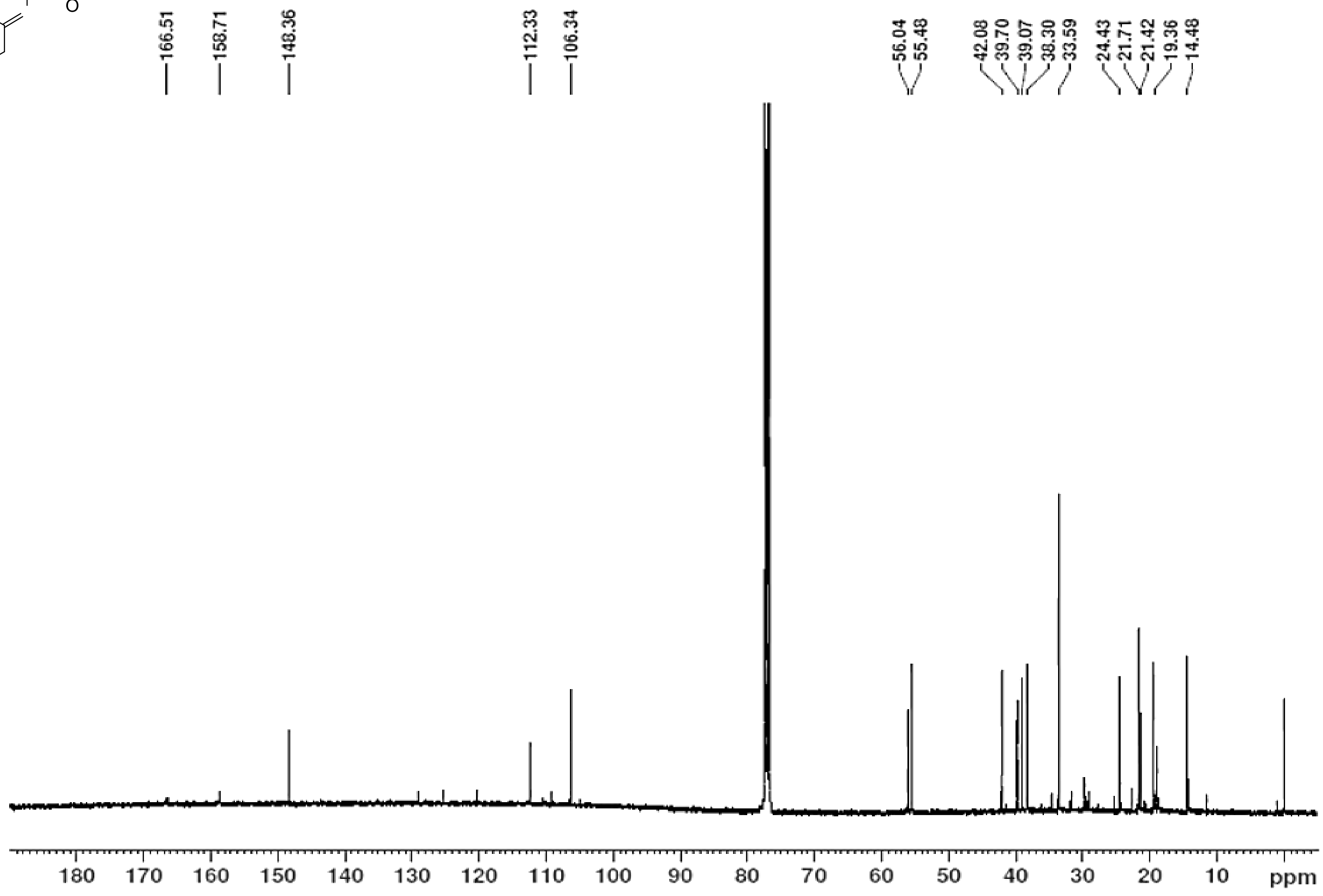

Mass spectrum of compound **4j**

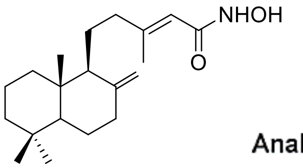

Analysis Info

Analysis Name	D:\Data\CRI\QCR01426 Pornsuda Copa-119-C1 E+.d	Acquisition Date	5/5/2017 5:44:04 PM
Method	Nitirat esi pos low may2017-1.m	Operator	BDAL@DE
Sample Name	ESIpos	Instrument	compact 8255754.20094
Comment			

Acquisition Parameter

Source Type	ESI	Ion Polarity	Positive	Set Nebulizer	1.0 Bar
Focus	Not active	Set Capillary	3500 V	Set Dry Heater	85 °C
Scan Begin	50 m/z	Set End Plate Offset	-500 V	Set Dry Gas	6.0 l/min
Scan End	800 m/z	Set Charging Voltage	2000 V	Set Divert Valve	Source
		Set Corona	0 nA	Set APCI Heater	0 °C

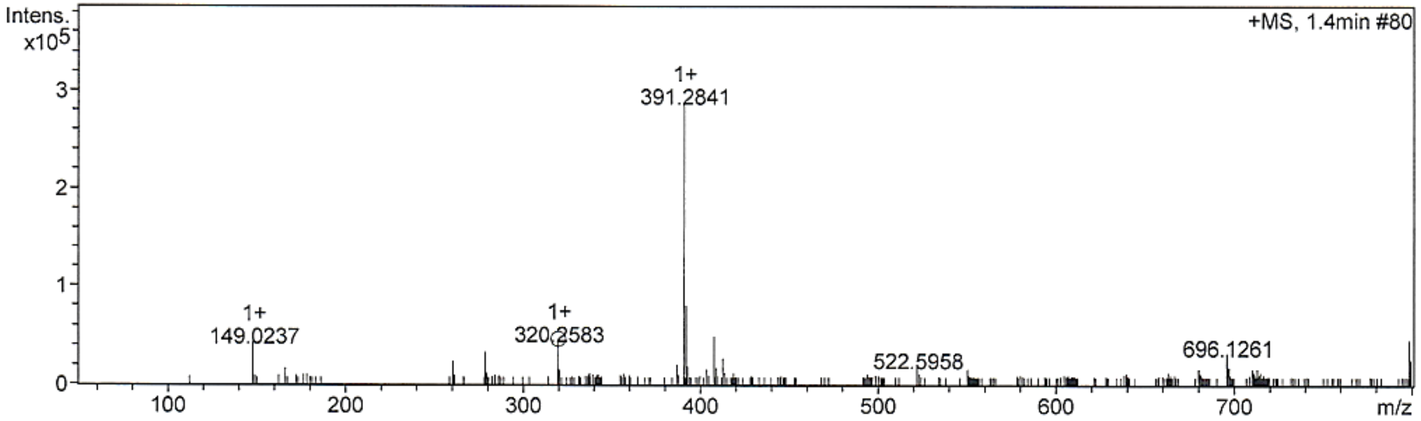

Meas. m/z	#	Ion Formula	Score	m/z	err [mDa]	err [ppm]	mSigma	rdb	e¯ Conf	N-Rule	Adduct
320.258347	1	C20H34NO2	100.00	320.258406	0.1	0.2	2.3	4.5	even	ok	M+H

^{1}H NMR of compound **4k** (400 MHz, $CDCl_3$)

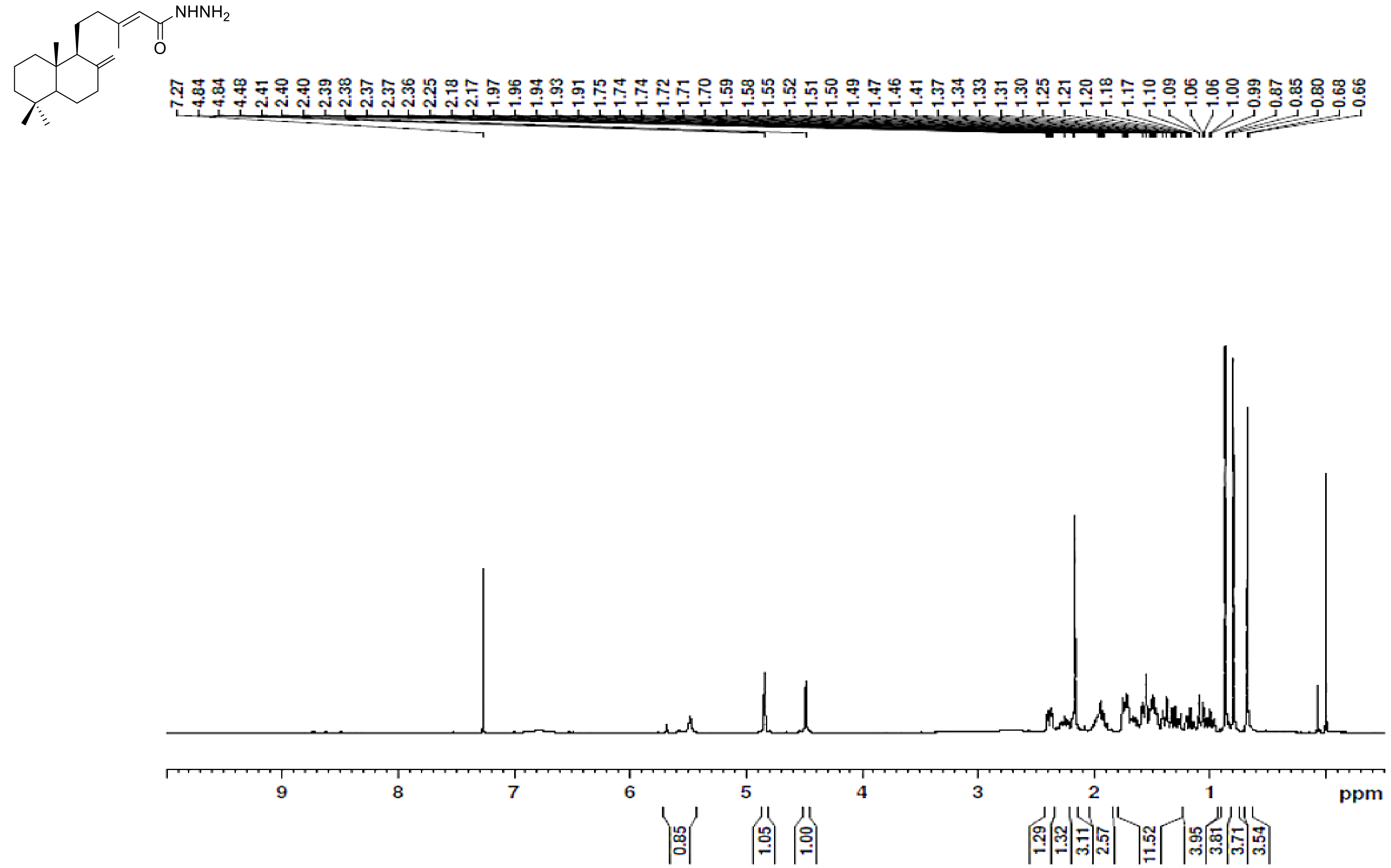

^{13}C NMR of compound **4k** (100 MHz, $CDCl_3$)

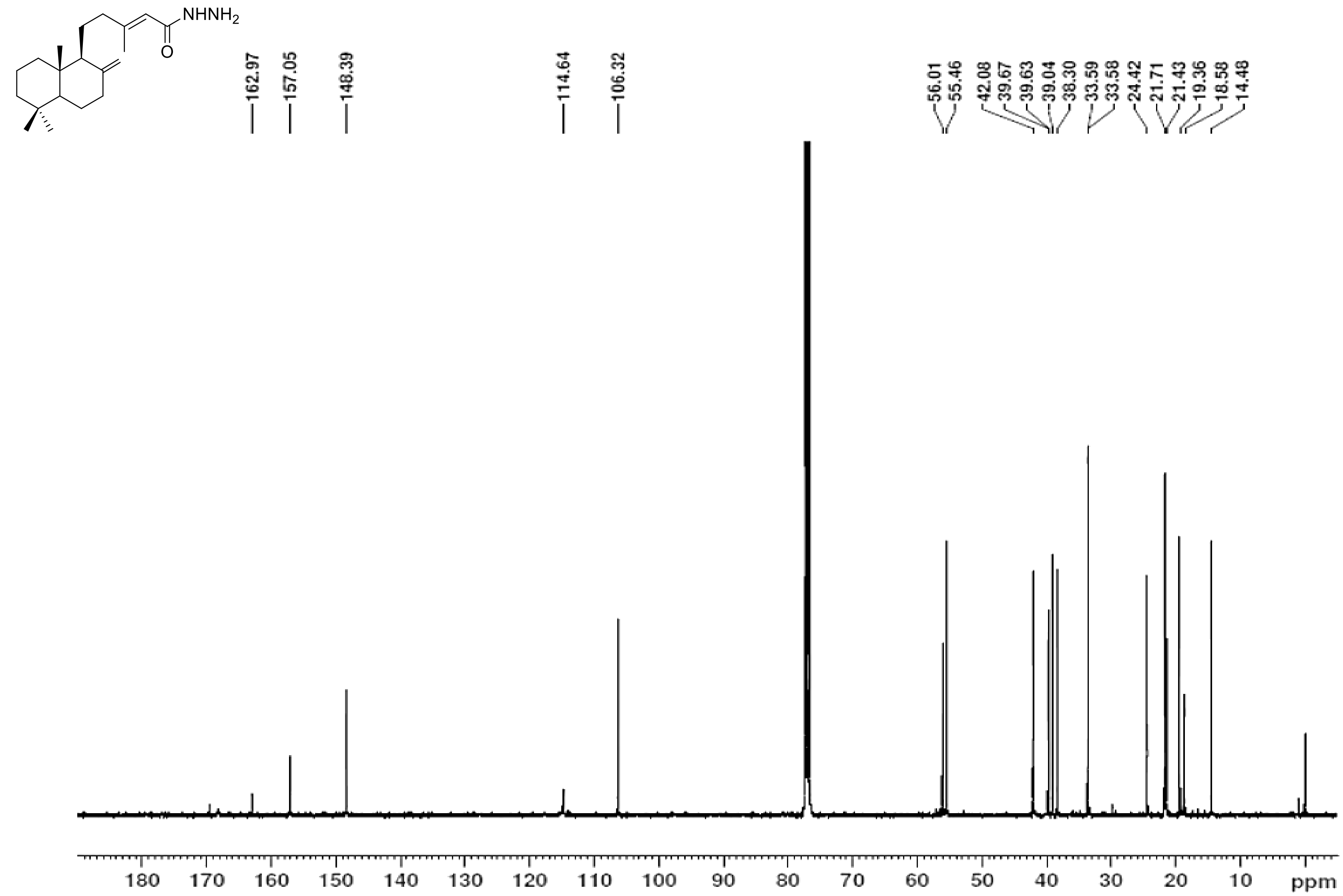

Mass spectrum of compound **4k**

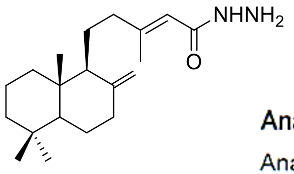

Analysis Info

Analysis Name	D:\Data\CRI\QCR01466 Pornsuda Copa-123c2 E+.d	Acquisition Date	5/15/2017 4:23:35 PM
Method	Nitirat esi pos low may2017-1.m	Operator	BDAL@DE
Sample Name	ESIpos	Instrument	compact 8255754.20094
Comment			

Acquisition Parameter

Source Type	ESI	Ion Polarity	Positive	Set Nebulizer	1.0 Bar
Focus	Not active	Set Capillary	3500 V	Set Dry Heater	80 °C
Scan Begin	100 m/z	Set End Plate Offset	-500 V	Set Dry Gas	6.0 l/min
Scan End	800 m/z	Set Charging Voltage	2000 V	Set Divert Valve	Source
		Set Corona	0 nA	Set APCI Heater	0 °C

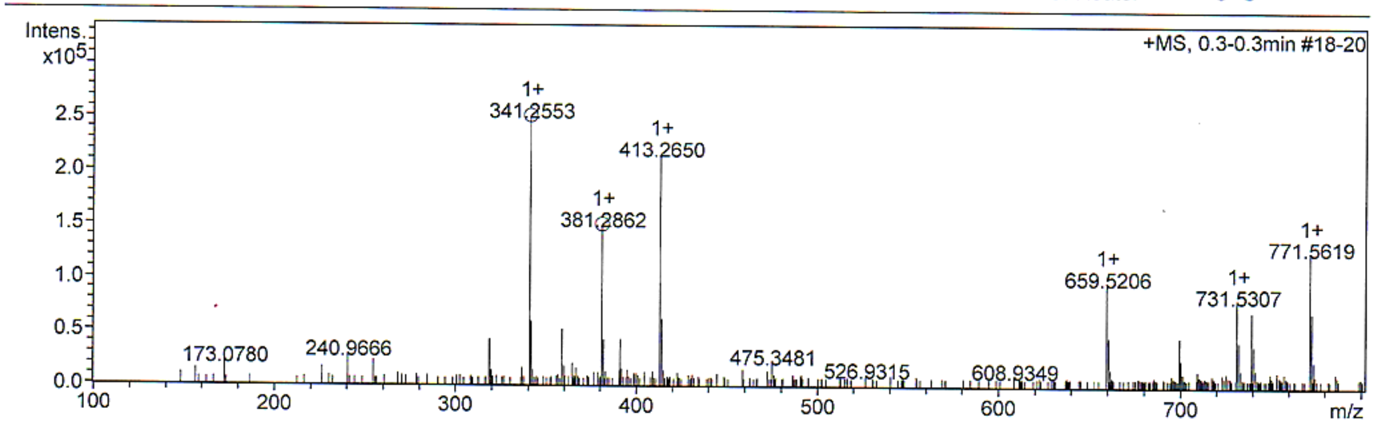

Meas. m/z	#	Ion Formula	Score	m/z	err [mDa]	err [ppm]	mSigma	rdb	e⁻ Conf	N-Rule	Adduct
341.255256	1	C20H34N2NaO	76.32	341.256334	1.1	3.2	8.5	4.5	even	ok	M+H
	2	C17H33N4O3	100.00	341.254717	-0.5	-1.6	9.5	3.5	even	ok	M+H
381.286162	1	C23H38N2NaO	55.43	381.287635	1.5	3.9	10.2	5.5	even	ok	M+H
	2	C20H37N4O3	100.00	381.286017	-0.1	-0.4	16.5	4.5	even	ok	M+H

^{1}H NMR of compound **4l** (400 MHz, $CDCl_3$)

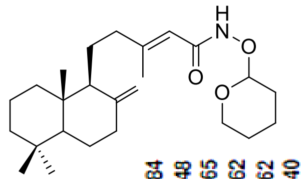

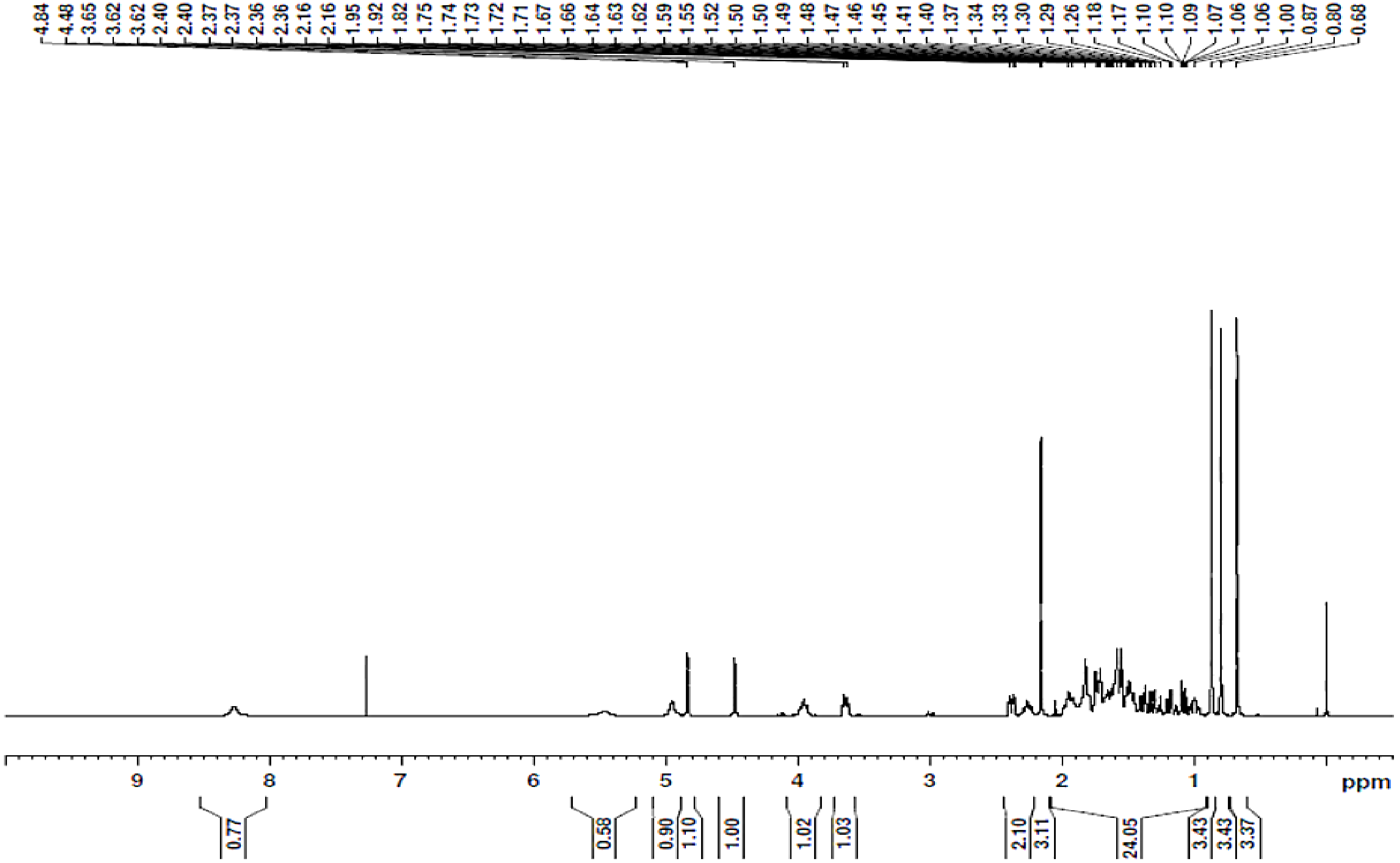

^{13}C NMR of compound **4l** (100 MHz, $CDCl_3$)

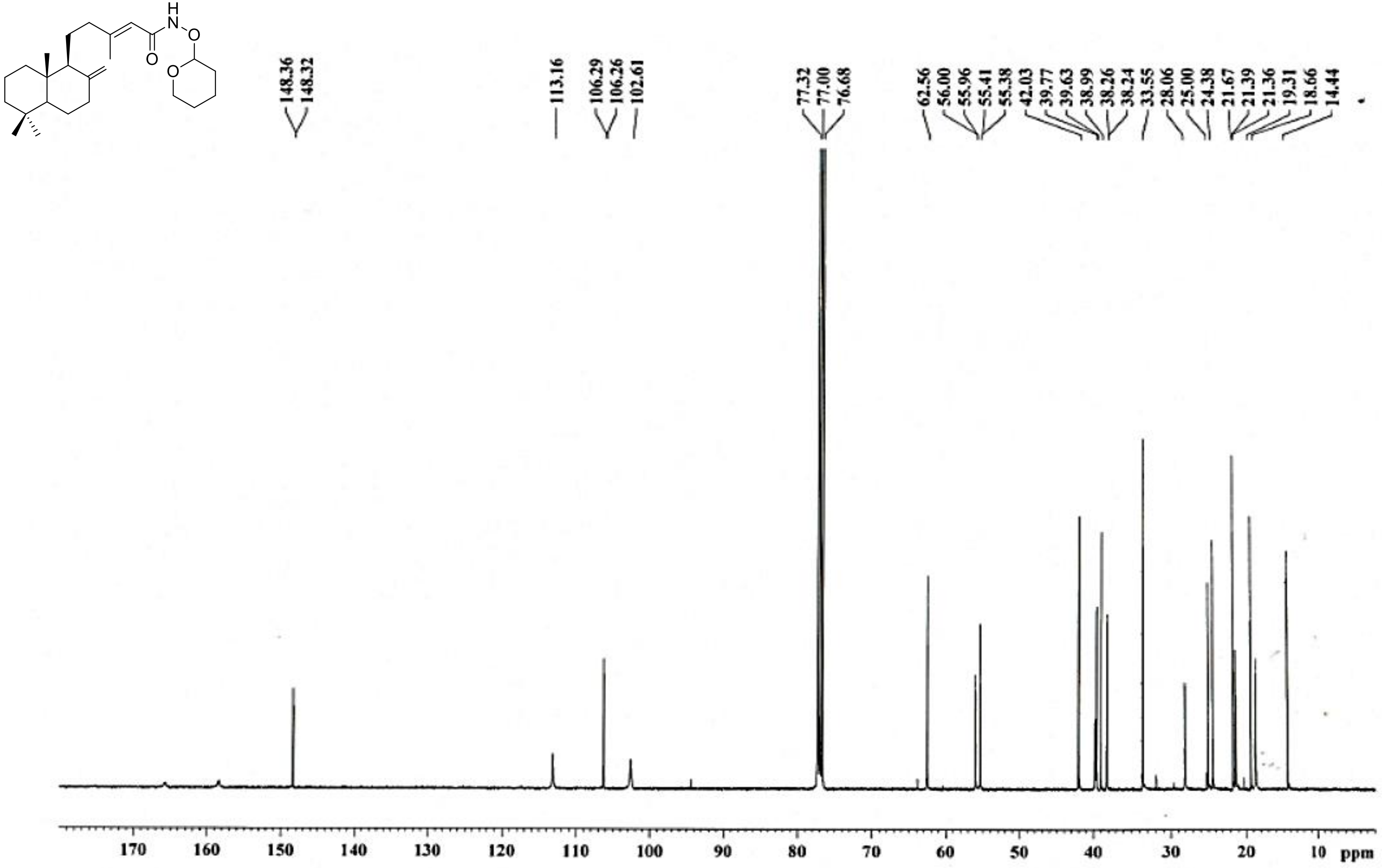

Mass spectrum of compound **4l**

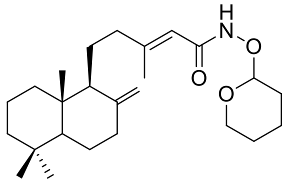

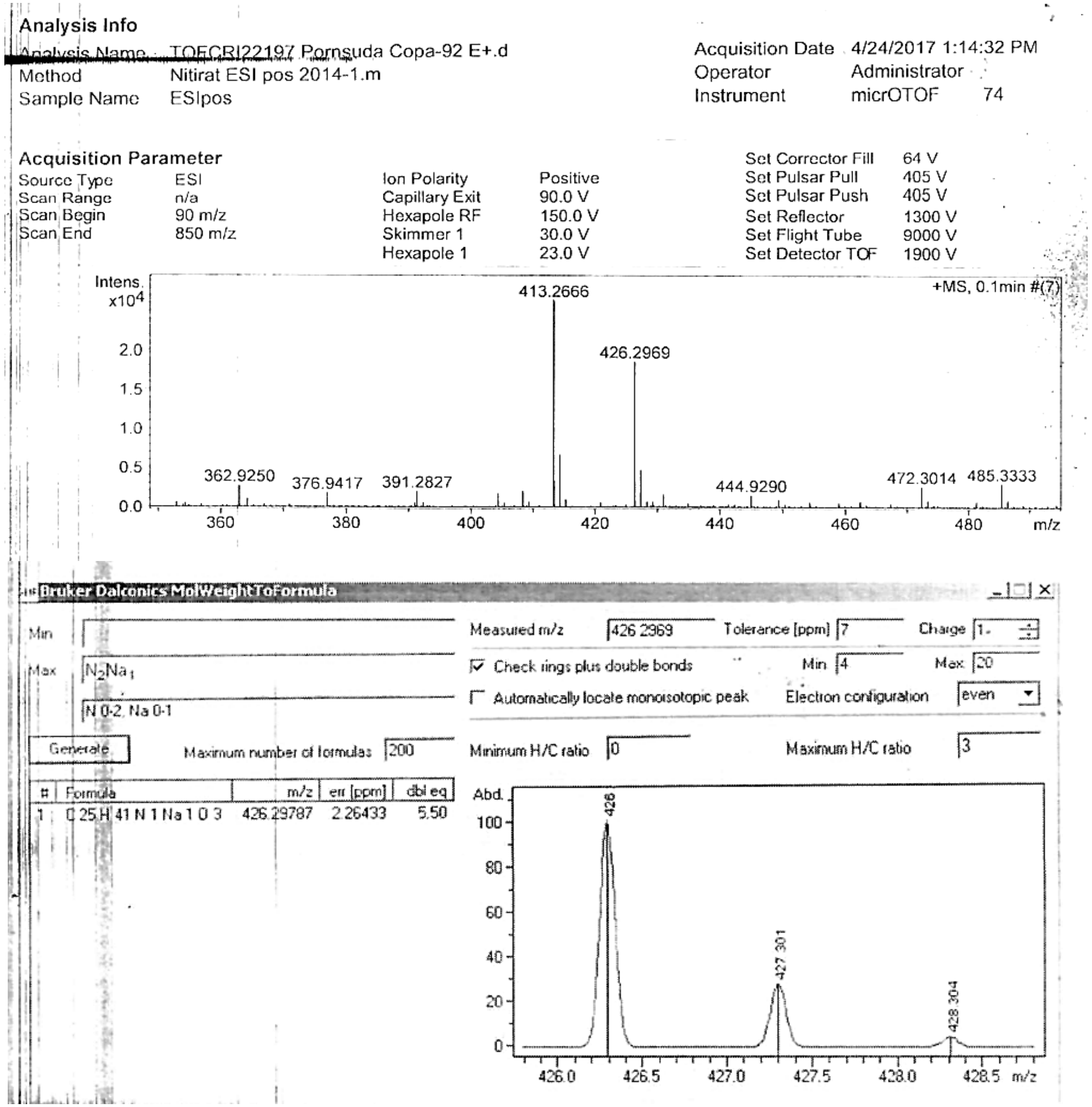

^{1}H NMR of compound **4m** (400 MHz, $CDCl_3$)

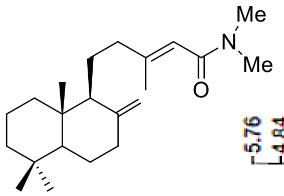

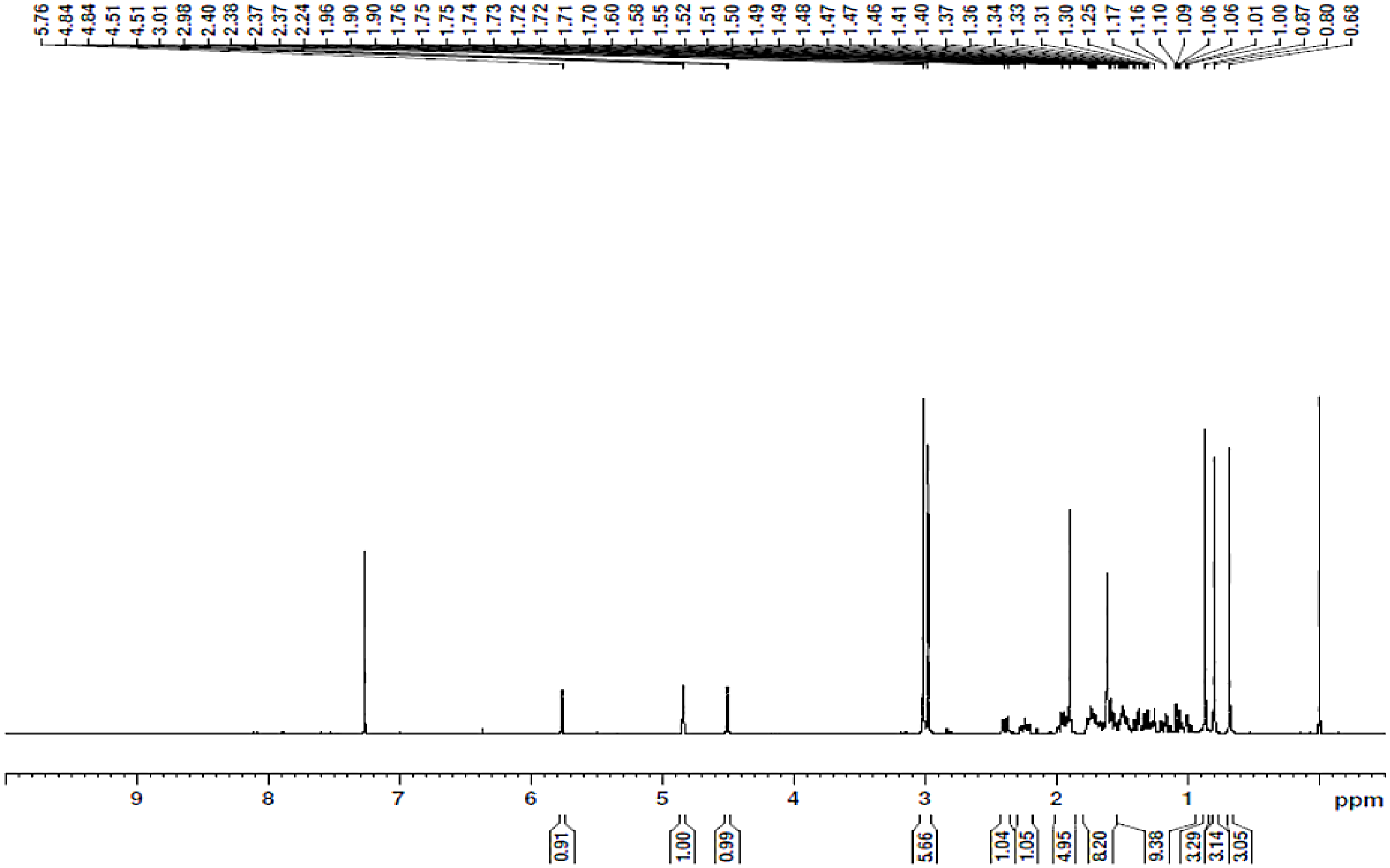

^{13}C NMR of compound **4m** (100 MHz, $CDCl_3$)

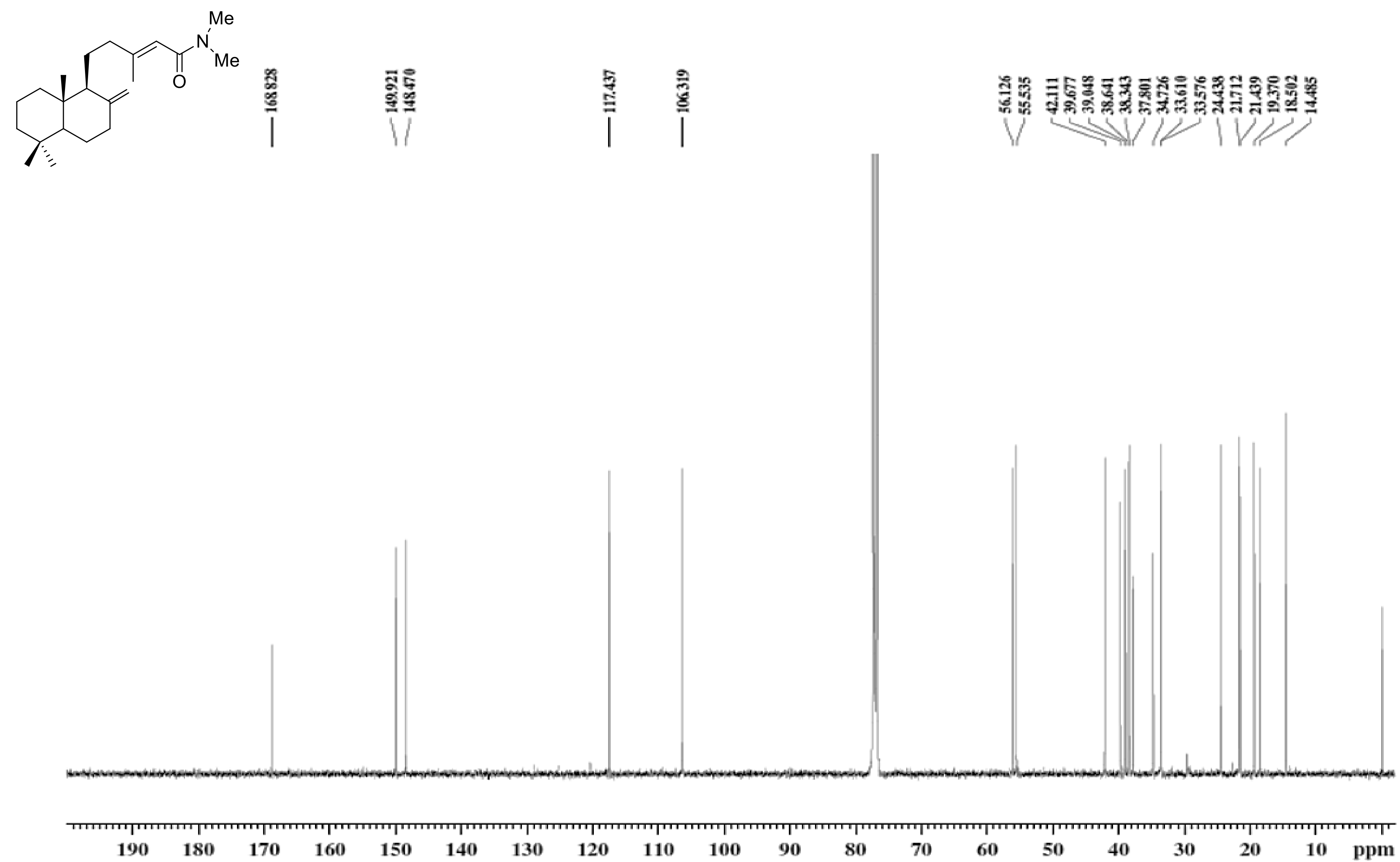

Mass spectrum of compound **4m**

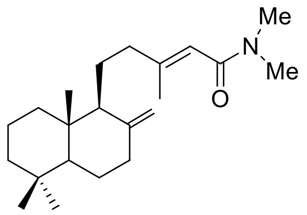

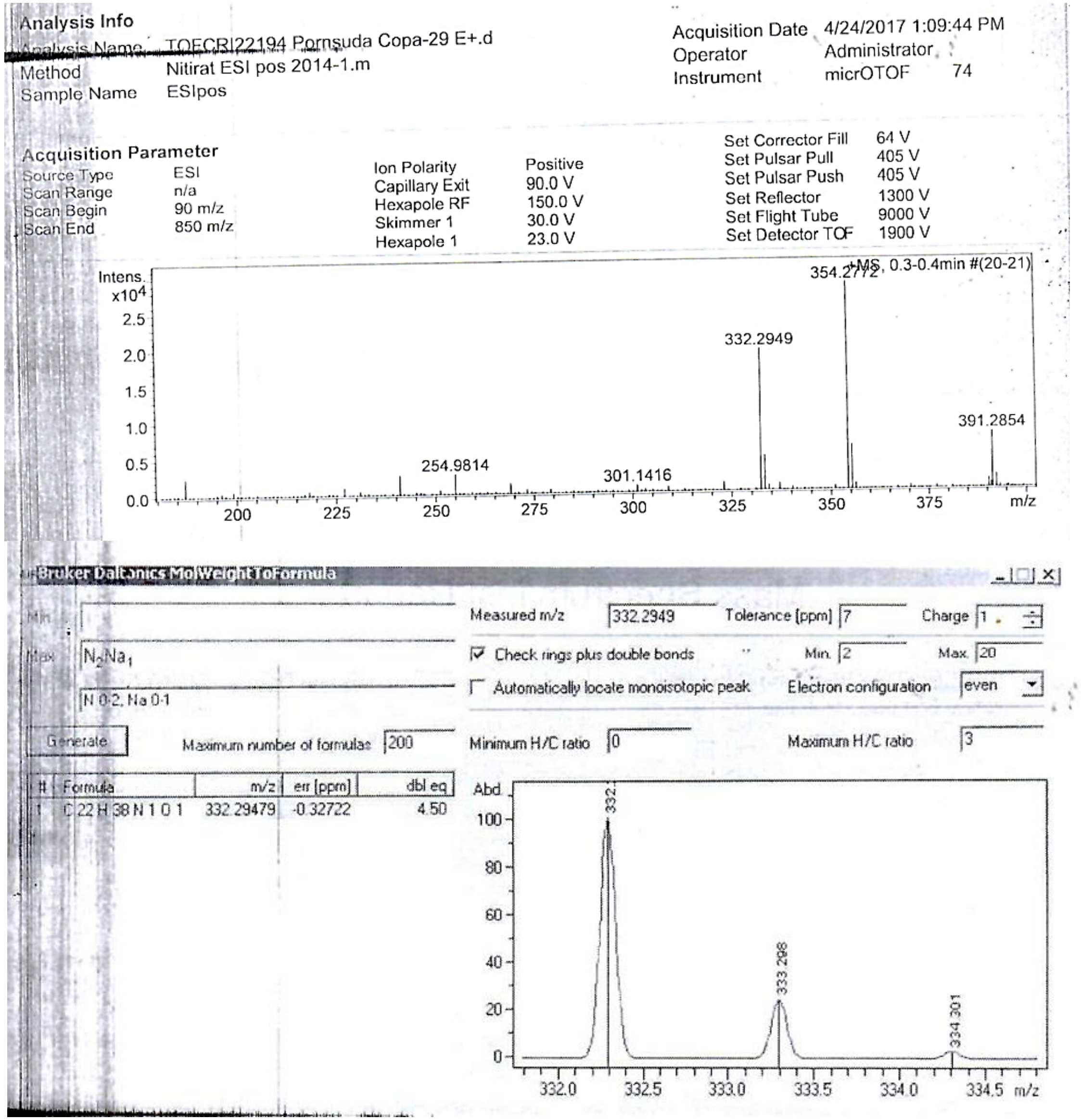

^{1}H NMR of compound **4n** (400 MHz, $CDCl_3$)

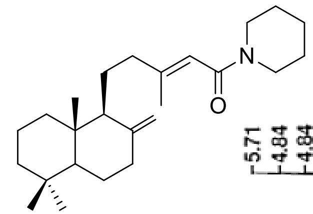

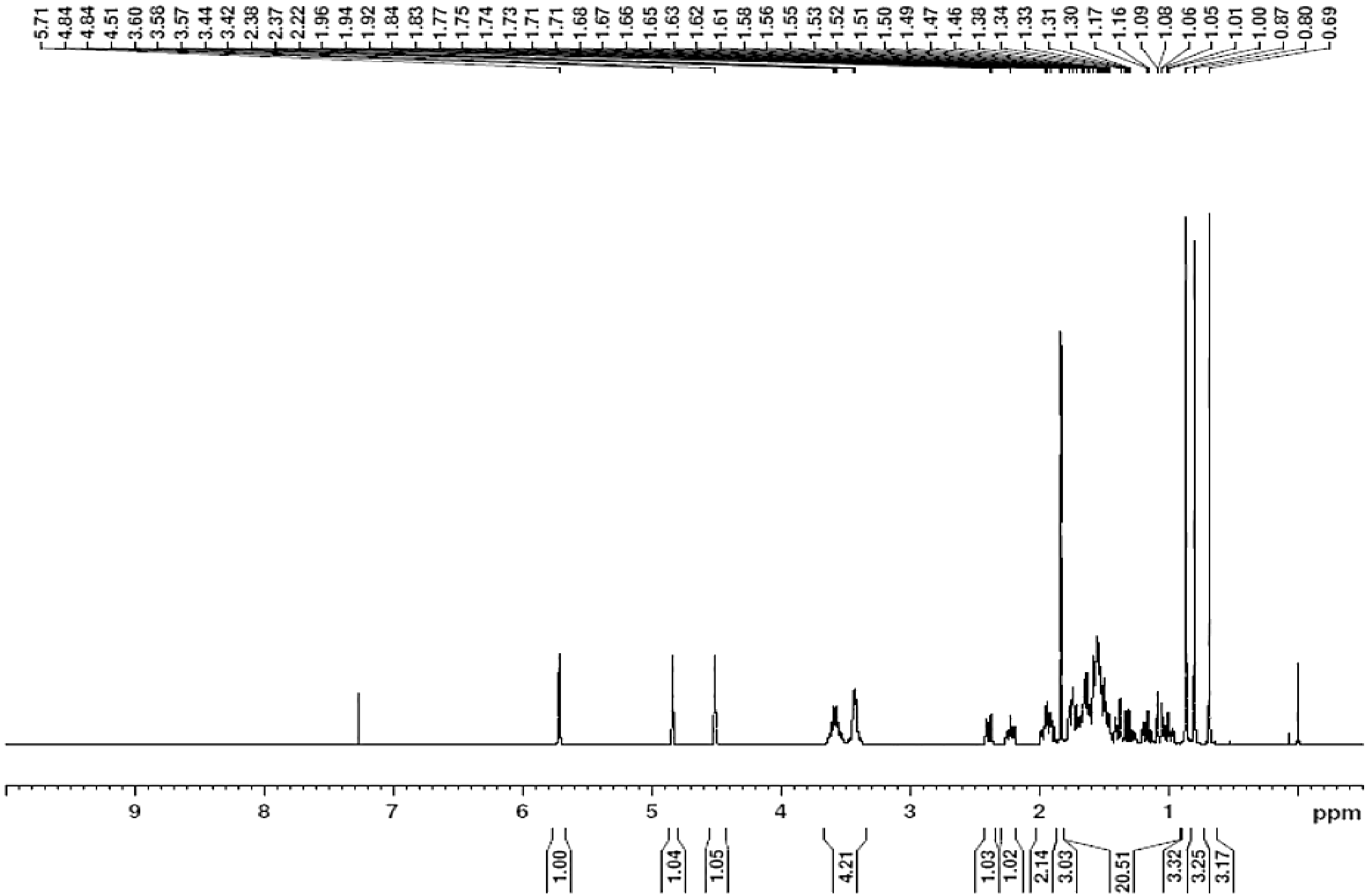

^{13}C NMR of compound **4n** (100 MHz, $CDCl_3$)

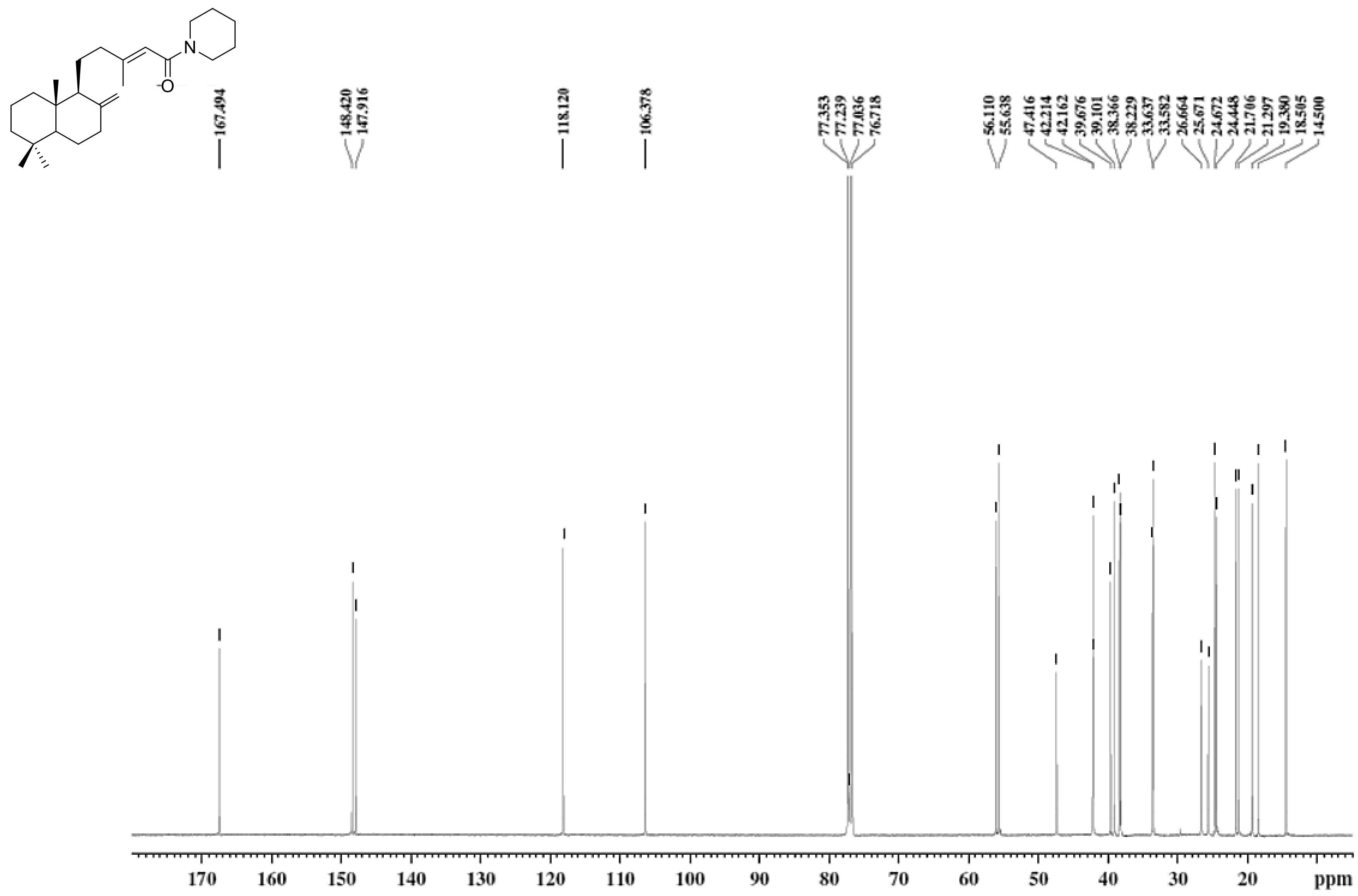

Mass spectrum of compound **4n**

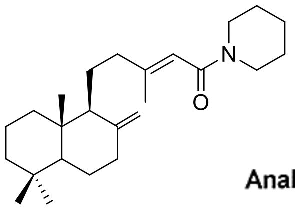

Analysis Info

Analysis Name	D:\Data\CRI\QCR00703 Pornsuda Copa-62-C1 E+.d	Acquisition Date	1/17/2017 2:15:36 PM	
Method	Nitirat esi pos low may2016.m	Operator	BDAL@DE	
Sample Name	ESIpos	Instrument	compact	8255754.20094
Comment				

Acquisition Parameter

Source Type	ESI	Ion Polarity	Positive	Set Nebulizer	0.5 Bar
Focus	Not active	Set Capillary	4500 V	Set Dry Heater	89 °C
Scan Begin	50 m/z	Set End Plate Offset	-500 V	Set Dry Gas	6.0 l/min
Scan End	800 m/z	Set Charging Voltage	2000 V	Set Divert Valve	Source
		Set Corona	0 nA	Set APCI Heater	0 °C

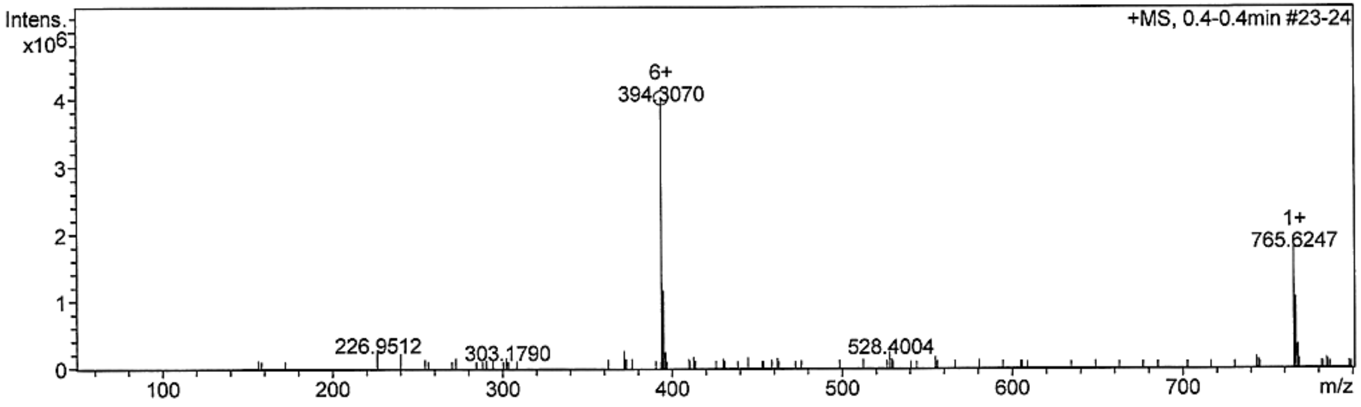

Meas. m/z	#	Ion Formula	Score	m/z	err [mDa]	err [ppm]	mSigma	rdb	e⁻ Conf	N-Rule	Adduct
394.306961	1	C25H41NNaO	100.00	394.308036	1.1	2.7	1.9	5.5	even	ok	M+H

^{1}H NMR of compound **4o** (400 MHz, $CDCl_3$)

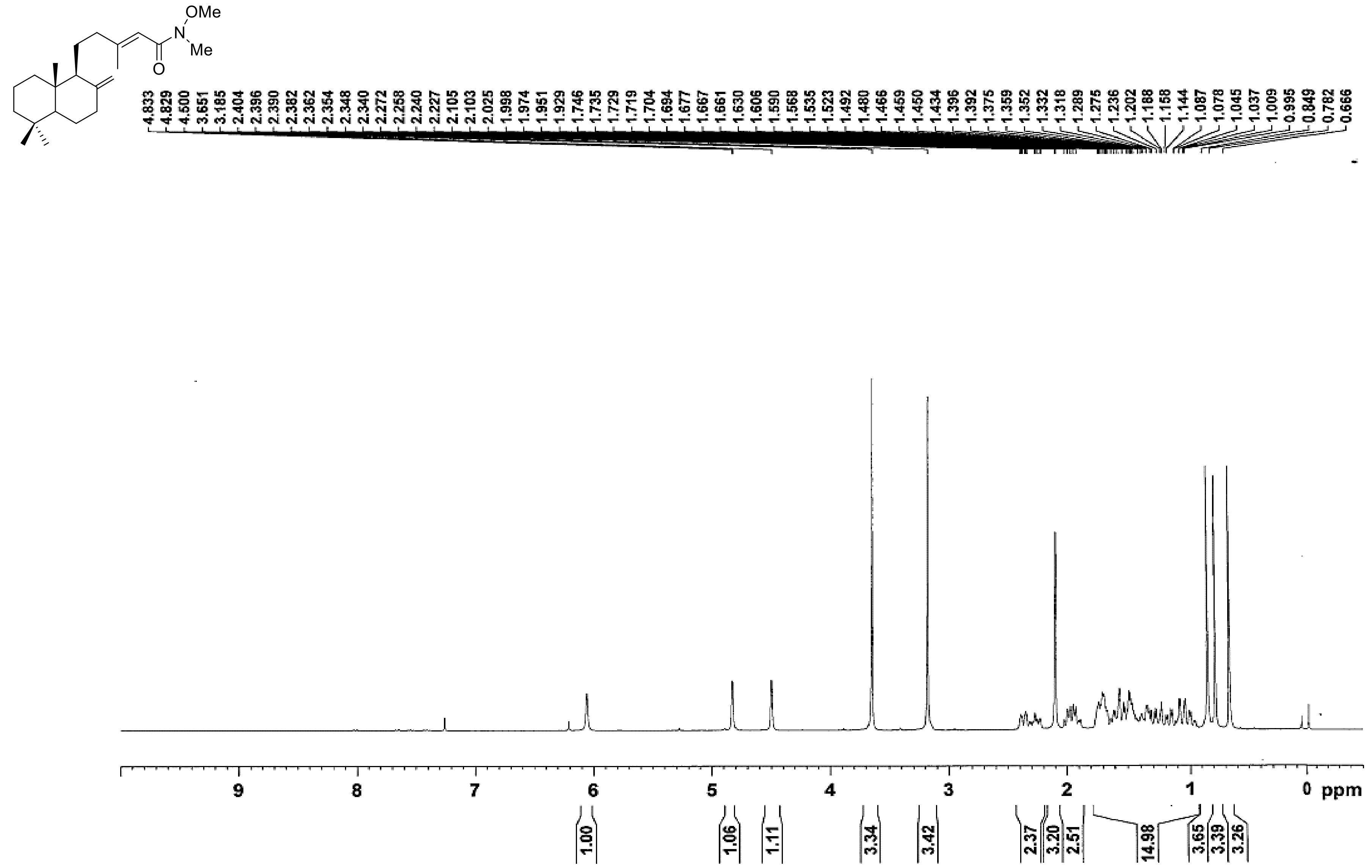

^{13}C NMR of compound **4o** (100 MHz, $CDCl_3$)

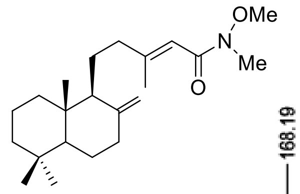

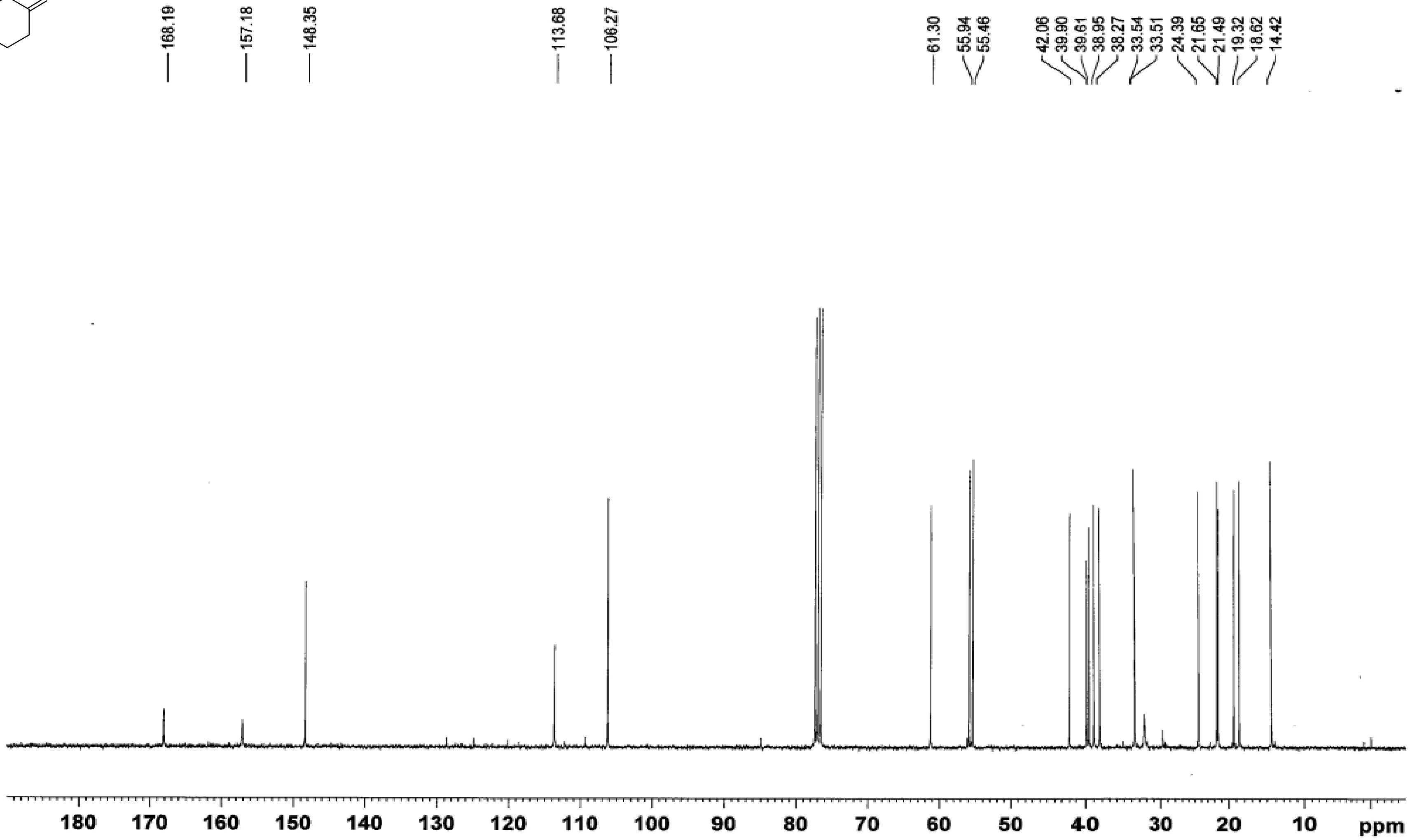

Mass spectrum of compound **4o**

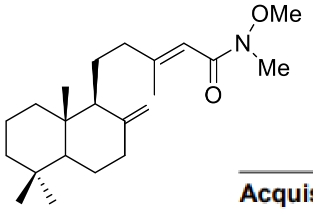

Acquisition Parameter

Source Type	ESI	Ion Polarity	Positive	Set Nebulizer	0.8 Bar
Focus	Not active			Set Dry Heater	100 °C
Scan Begin	120 m/z	Set Capillary	3000 V	Set Dry Gas	6.5 l/min
Scan End	800 m/z	Set End Plate Offset	-500 V	Set Divert Valve	Source

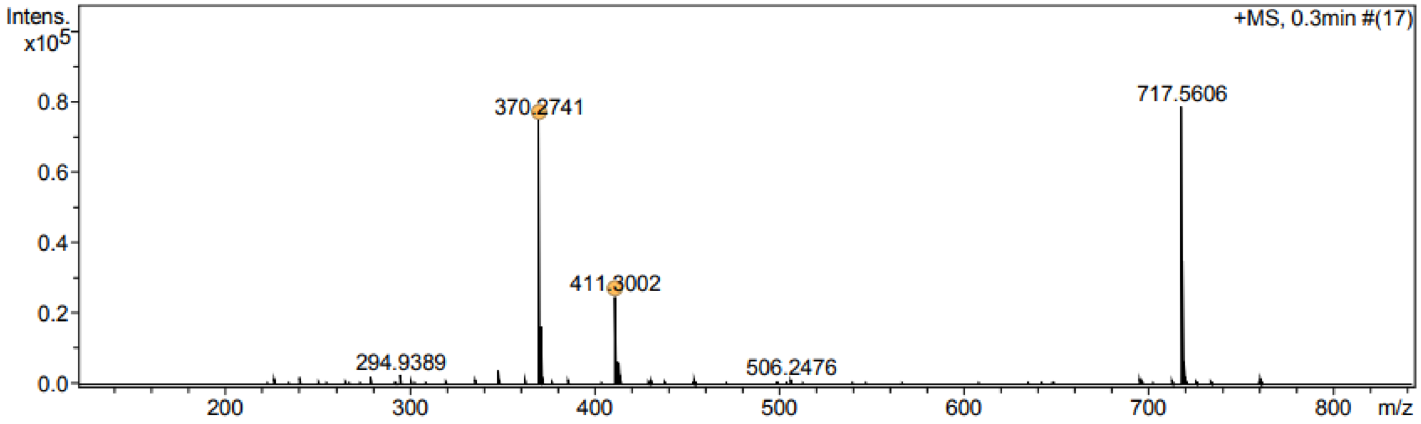

Meas. m/z	#	Ion Formula	Score	m/z	err [mDa]	err [ppm]	mSigma	rdb	e¯ Conf	N-Rule	Adduct
370.274145	1	C22H37NNaO2	28.98	370.271650	-2.5	-6.7	14.2	4.5	even	ok	M+H
	2	C24H36NO2	100.00	370.274056	-0.1	-0.2	27.0	7.5	even	ok	M+H
411.300152	1	C24H40N2NaO2	48.97	411.298199	-2.0	-4.7	23.7	5.5	even	ok	M+H
	2	C26H39N2O2	100.00	411.300605	0.5	1.1	32.2	8.5	even	ok	M+H
	3	C29H40Na	27.38	411.302222	2.1	5.0	43.2	9.5	even	ok	M+H

^{1}H NMR of compound **4p** (400 MHz, $CDCl_3$)

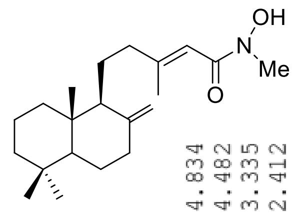

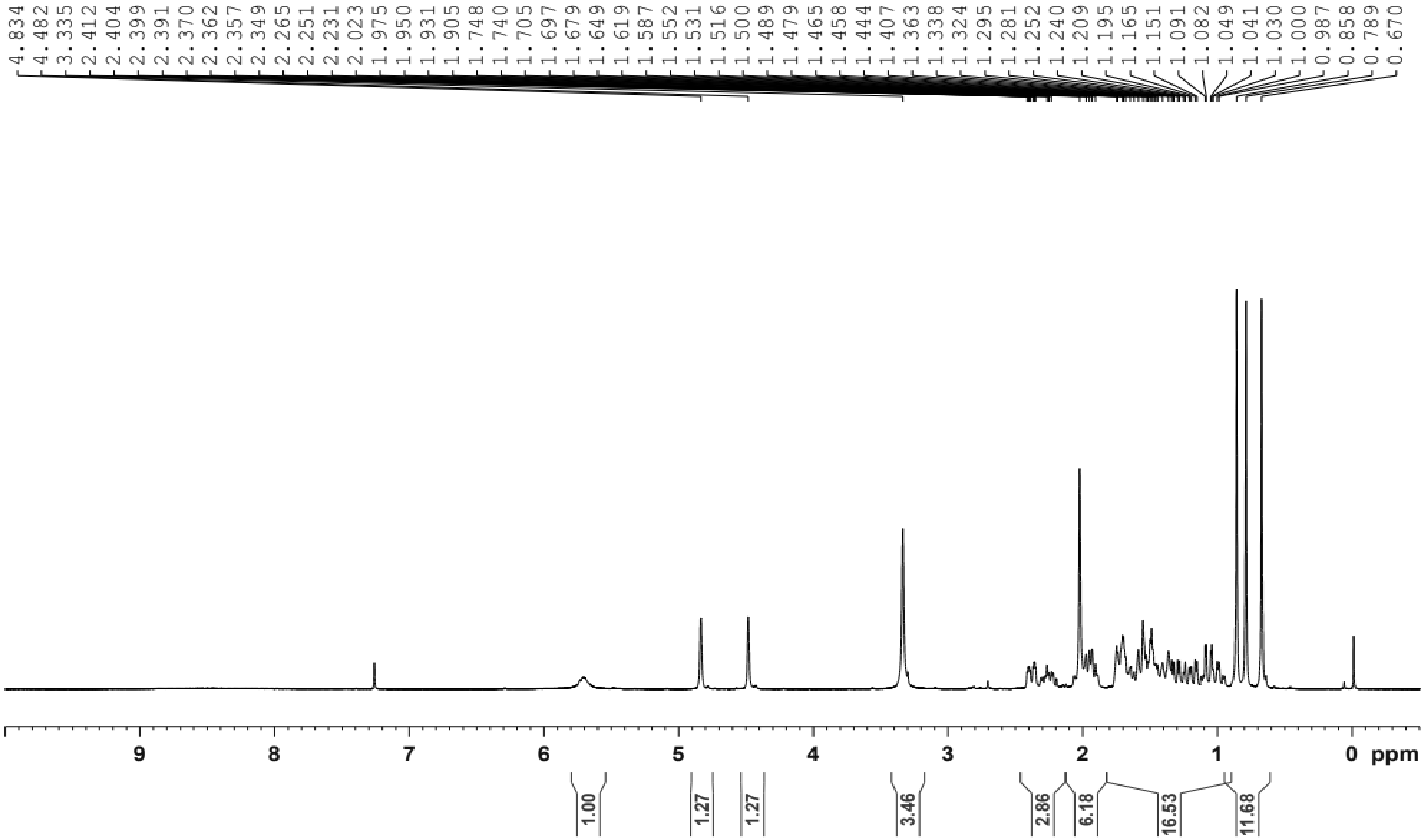

^{13}C NMR of compound **4p** (100 MHz, $CDCl_3$)

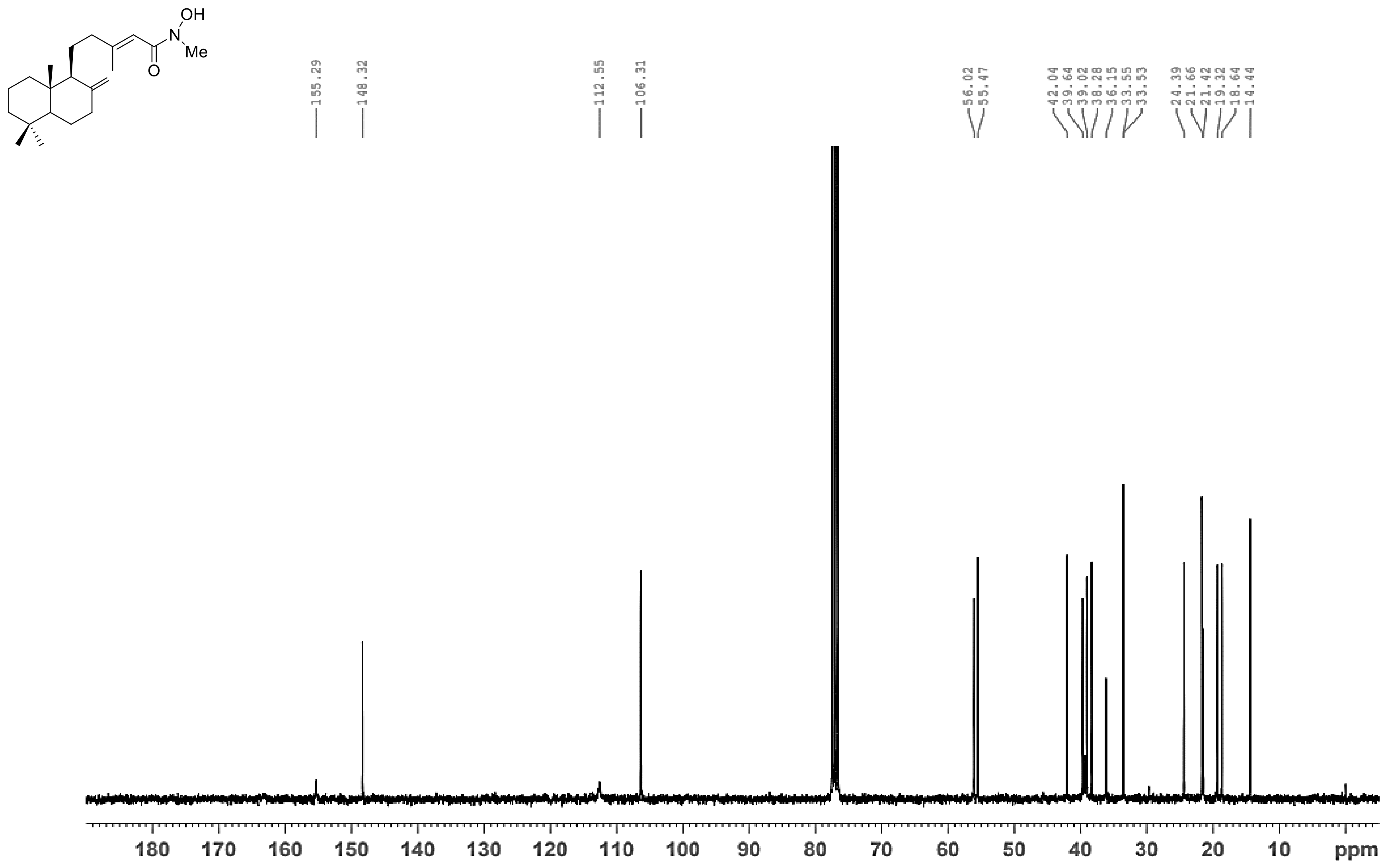

Mass spectrum of compound **4p**

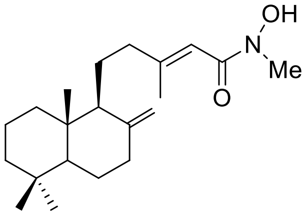

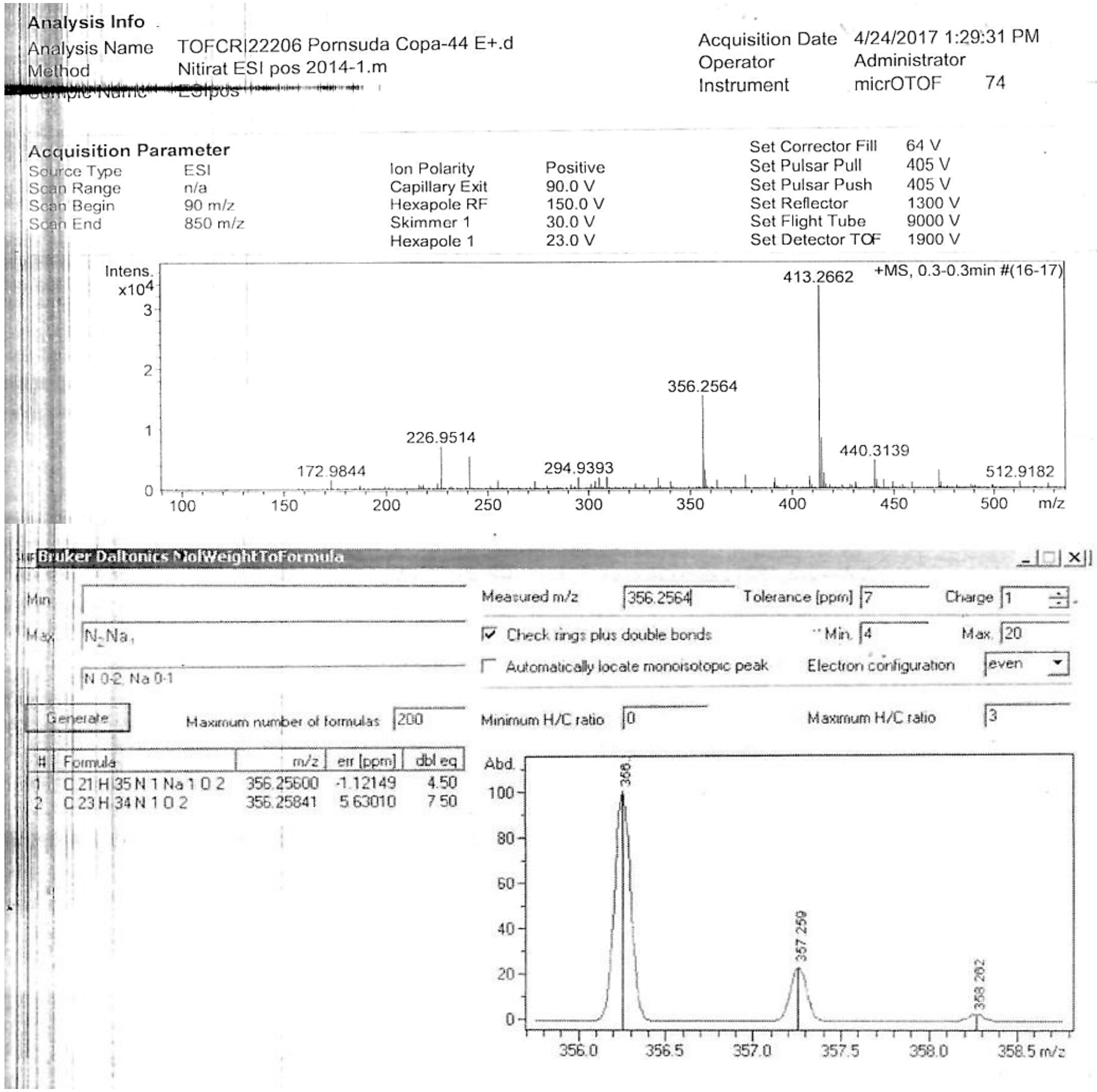

^{1}H NMR of compound **4q** (400 MHz, $CDCl_3$)

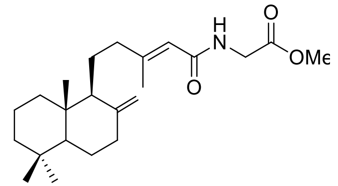

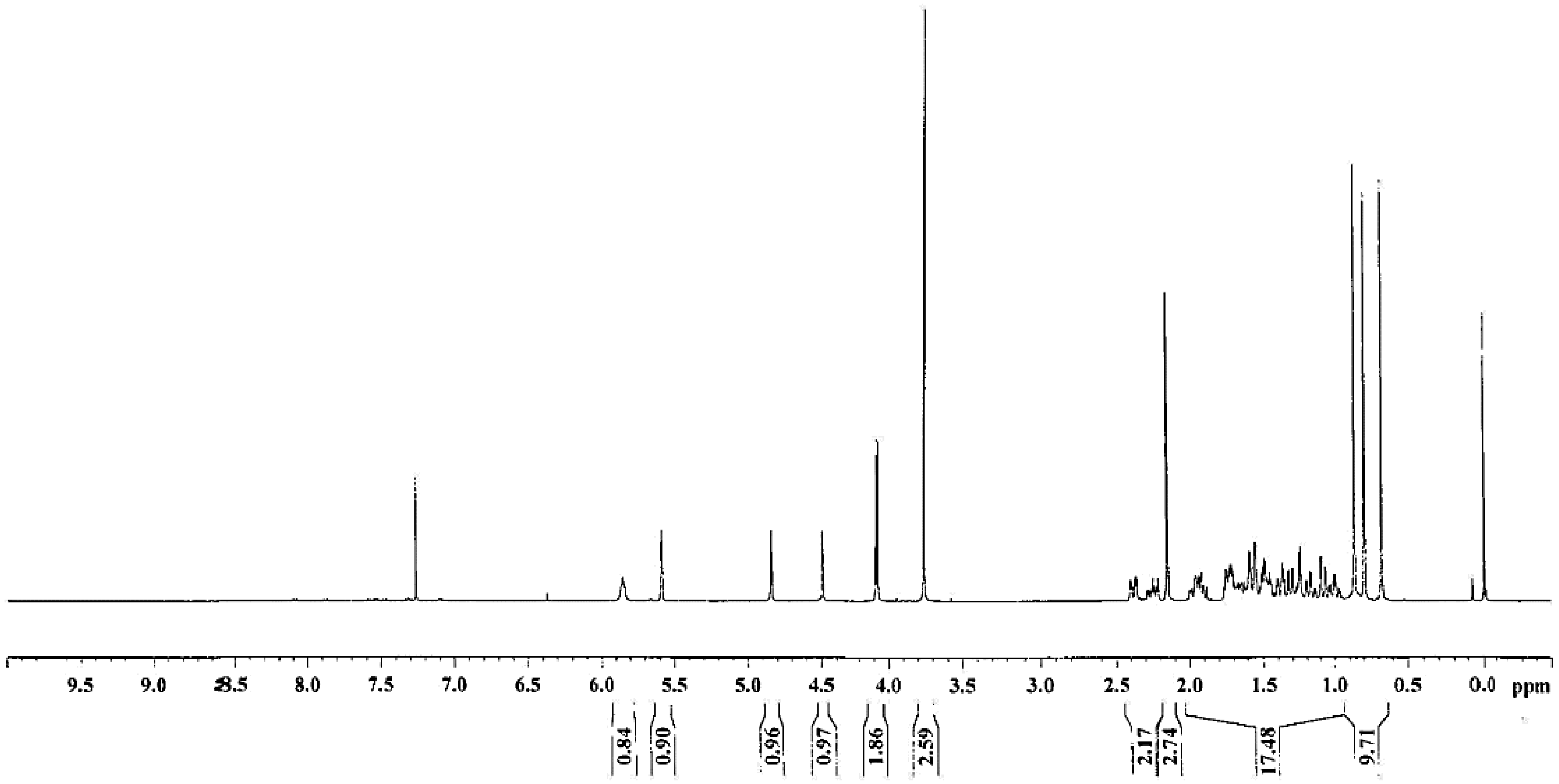

^{13}C NMR of compound **4q** (100 MHz, $CDCl_3$)

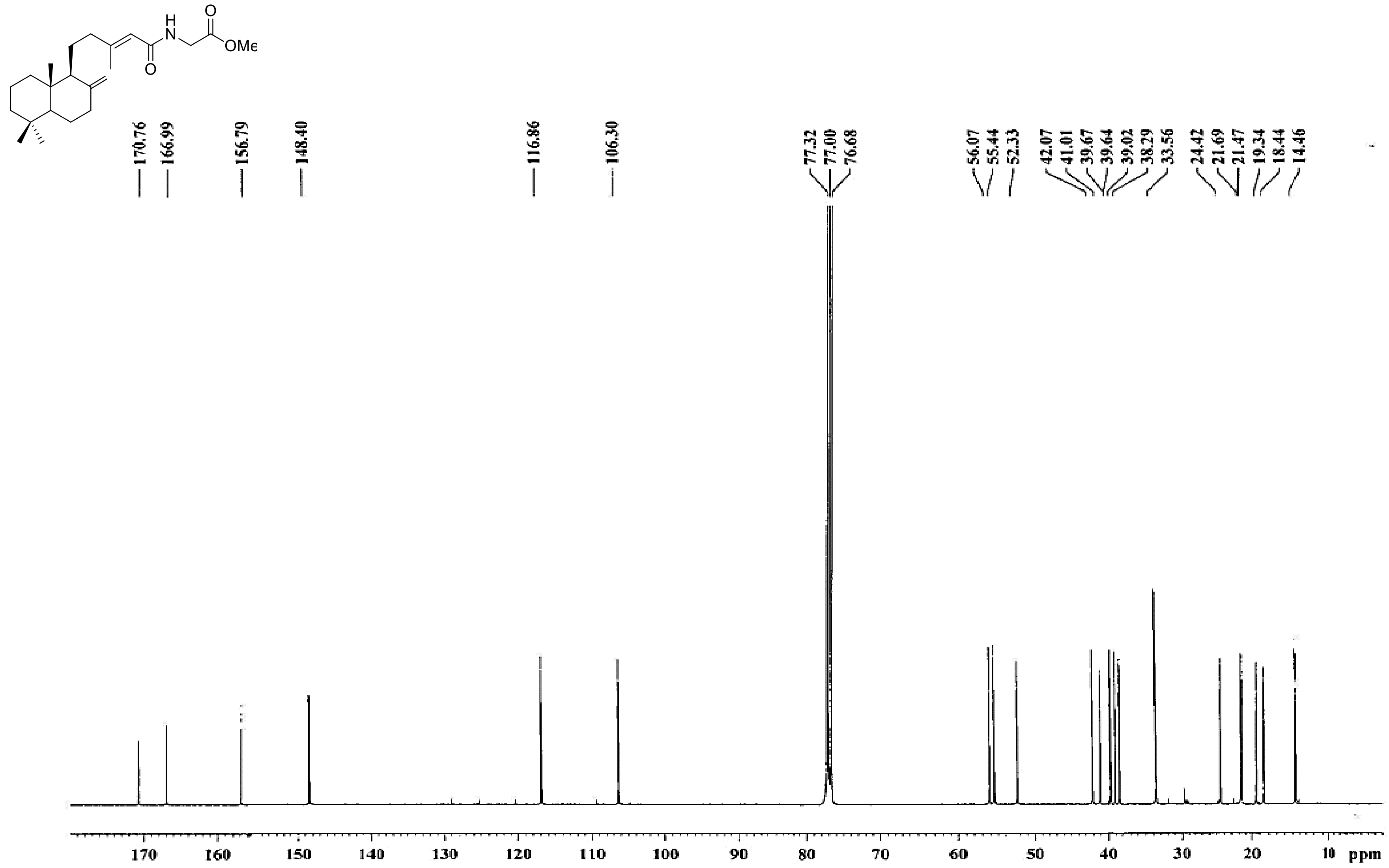

Mass spectrum of compound **4q**

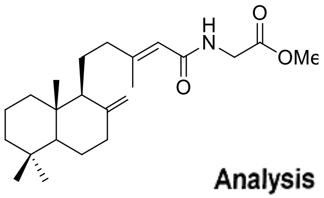

Analysis Info

Analysis Name	D:\Data\CRI\QCR00708 Pornsuda Copa-40C2C3 E+.d	Acquisition Date	1/17/2017 2:23:38 PM
Method	Nitirat esi pos low may2016.m	Operator	BDAL@DE
Sample Name	ESIpos	Instrument	compact 8255754.20094
Comment			

Acquisition Parameter

Source Type	ESI	Ion Polarity	Positive	Set Nebulizer	0.5 Bar
Focus	Not active	Set Capillary	4500 V	Set Dry Heater	89 °C
Scan Begin	50 m/z	Set End Plate Offset	-500 V	Set Dry Gas	6.0 l/min
Scan End	800 m/z	Set Charging Voltage	2000 V	Set Divert Valve	Source
		Set Corona	0 nA	Set APCI Heater	0 °C

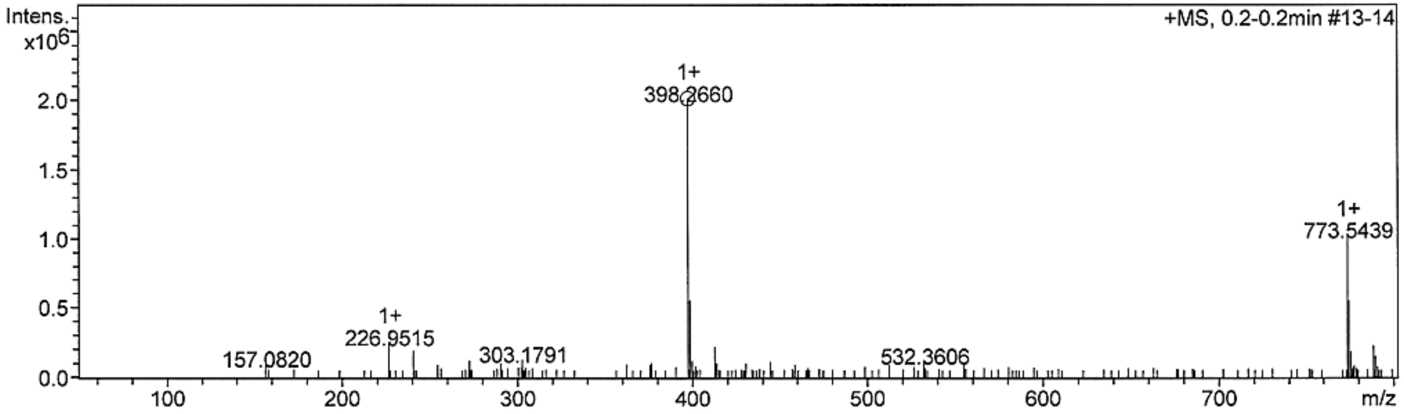

Meas. m/z	#	Ion Formula	Score	m/z	err [mDa]	err [ppm]	mSigma	rdb	e⁻ Conf	N-Rule	Adduct
398.265974	1	C23H37NNaO3	100.00	398.266565	0.6	1.5	0.3	5.5	even	ok	M+H

^{1}H NMR of compound **4r** (400 MHz, $CDCl_3$)

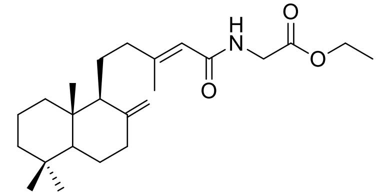

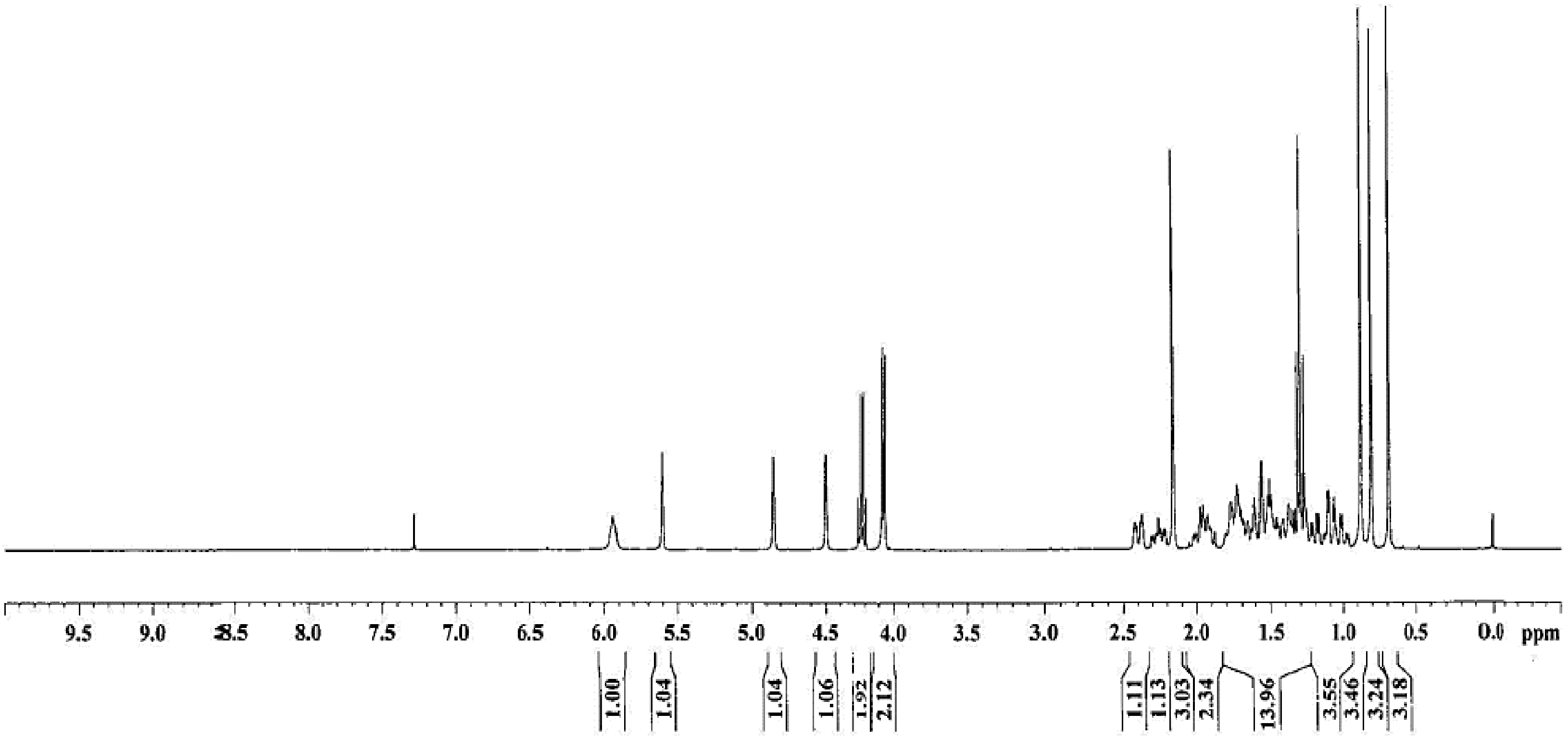

^{13}C NMR of compound **4r** (100 MHz, $CDCl_3$)

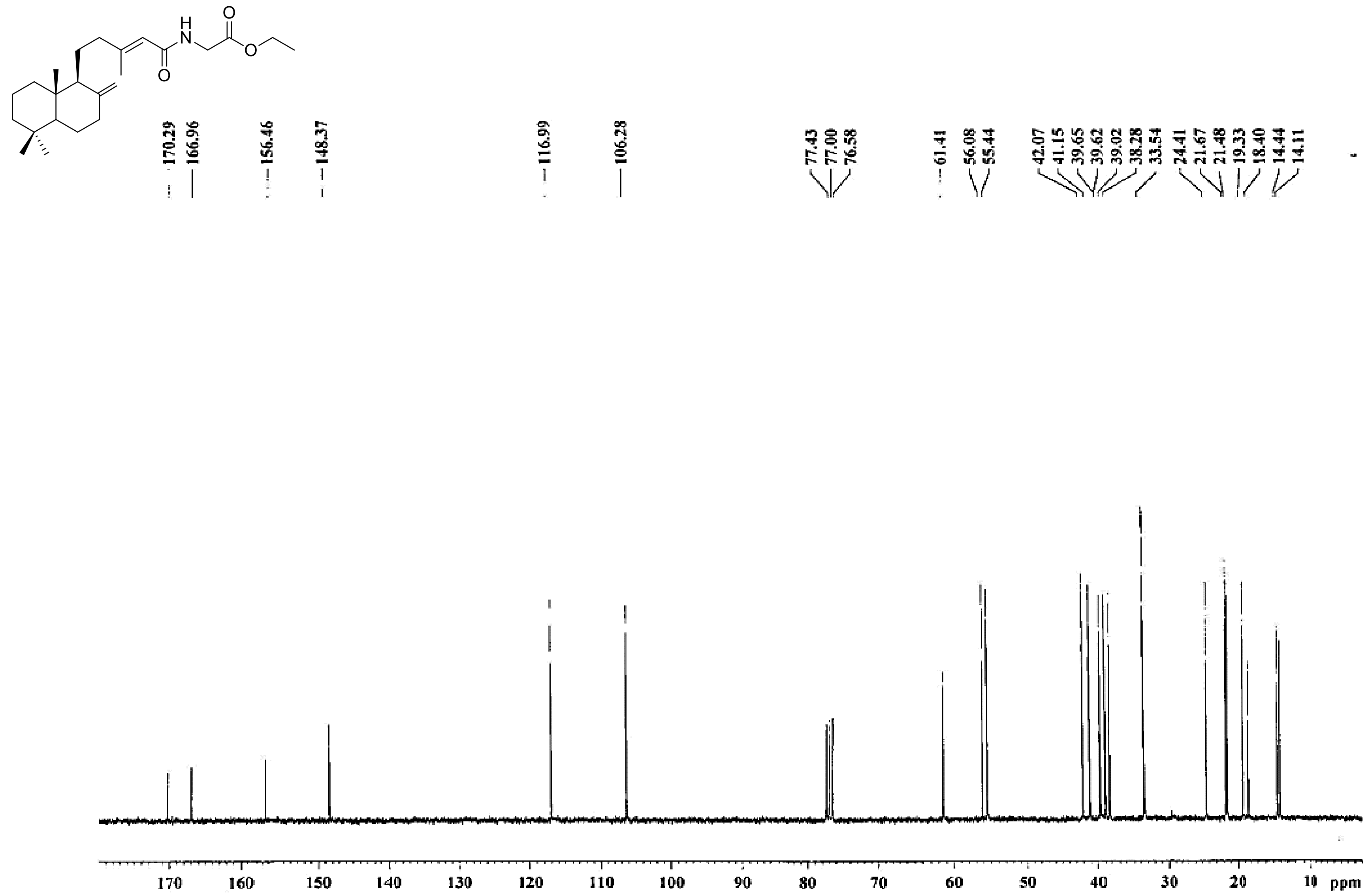

Mass spectrum of compound **4r**

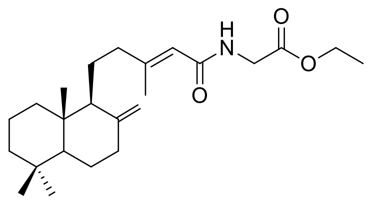

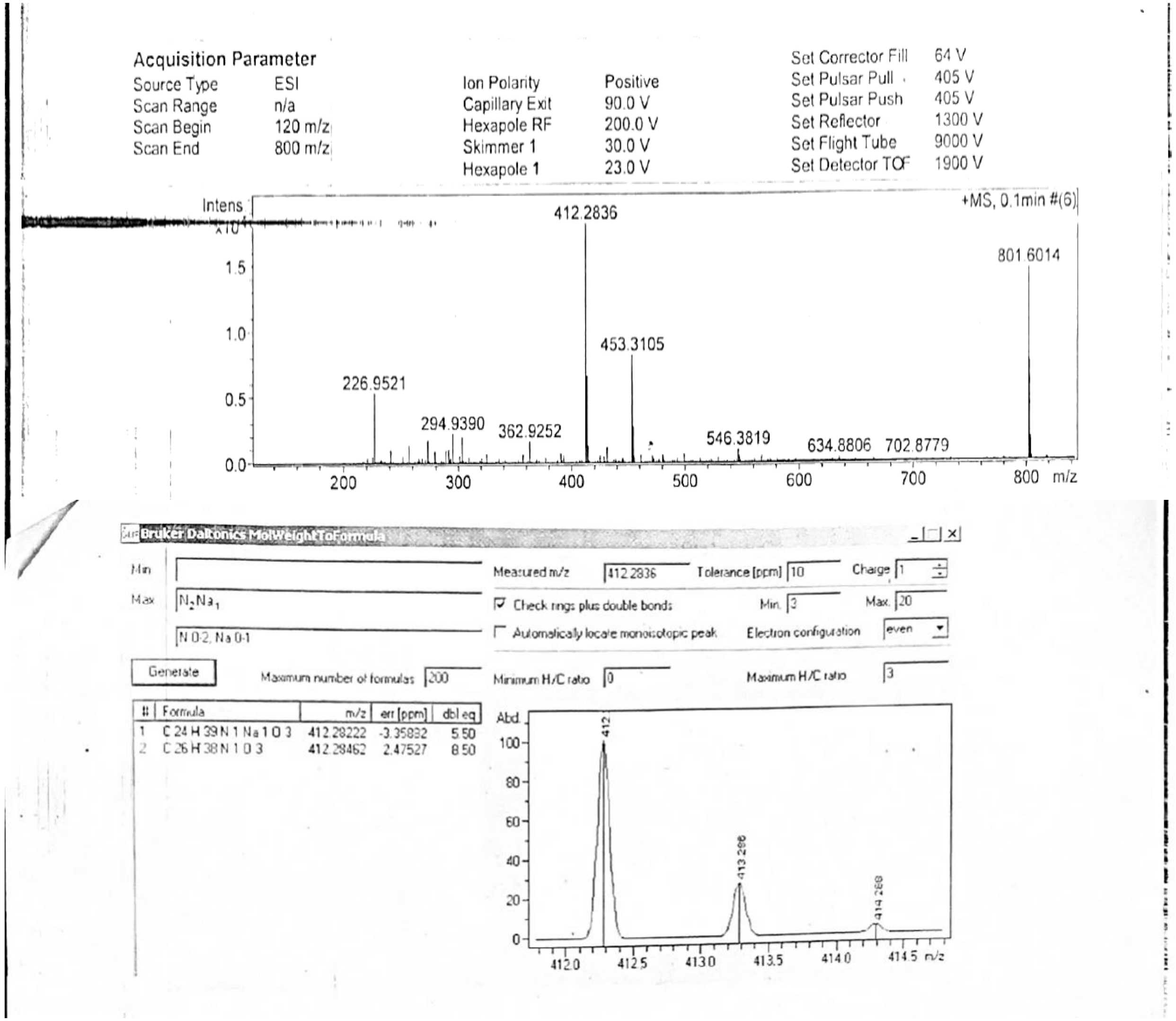

^{1}H NMR of compound **5s** (400 MHz, $CDCl_3$)

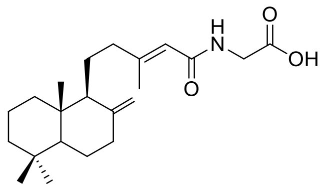

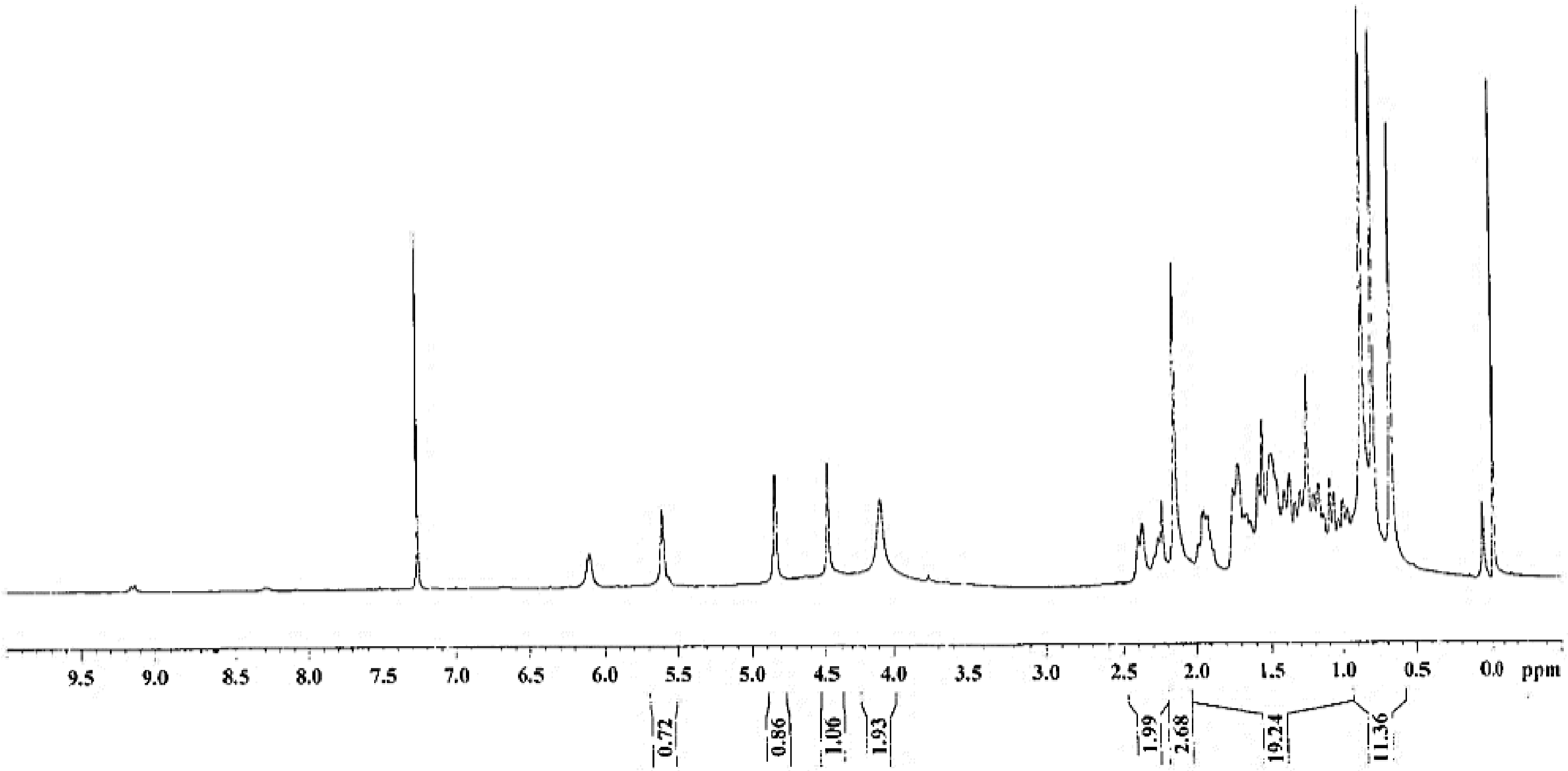

^{13}C NMR of compound **5s** (100 MHz, $CDCl_3$)

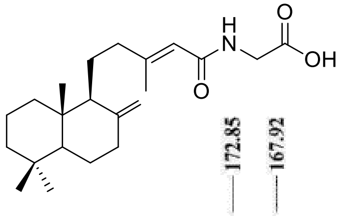

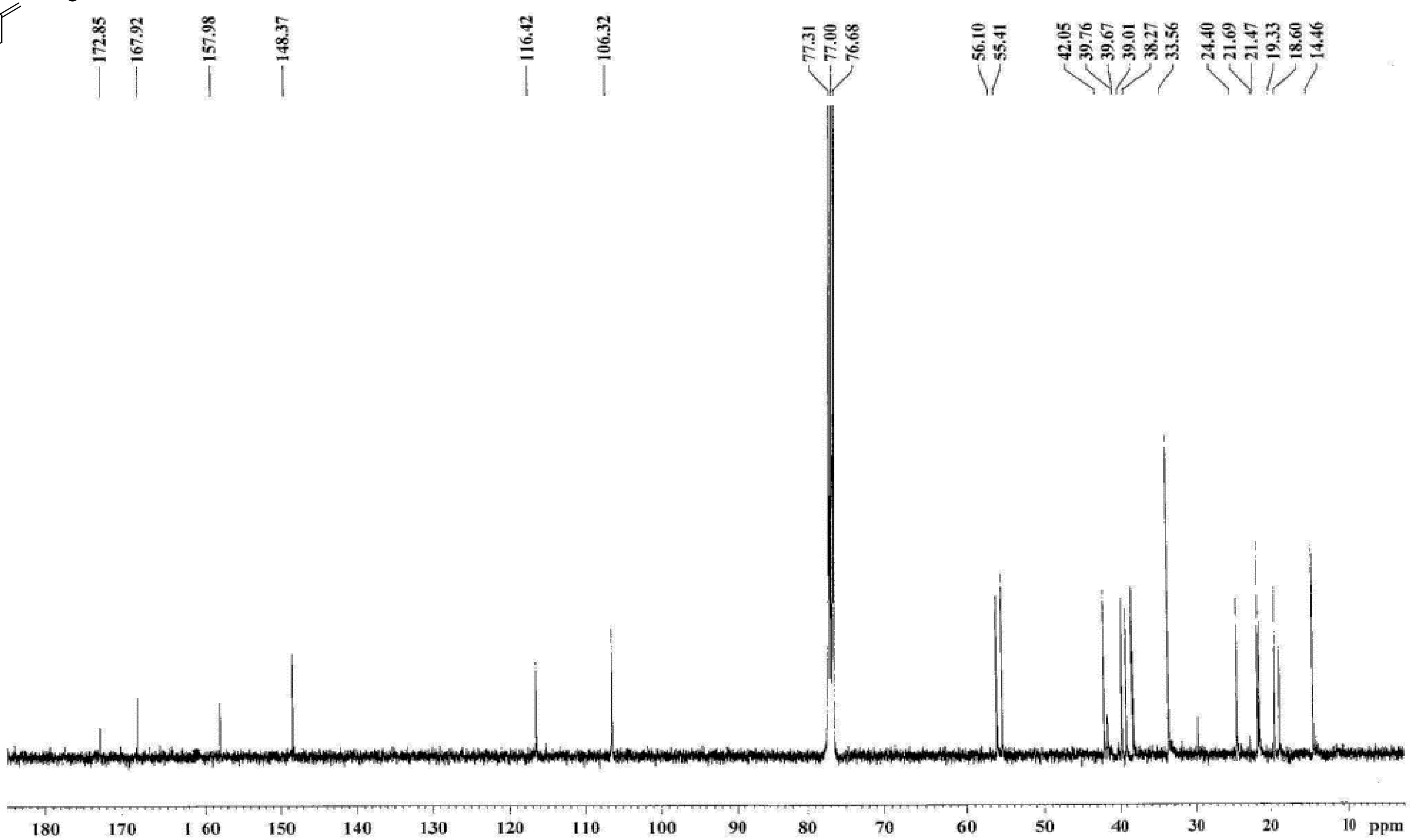

Mass spectrum of compound **5s**

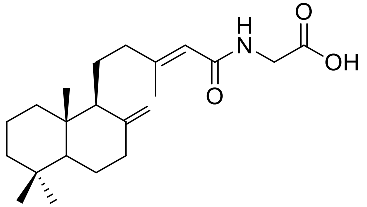

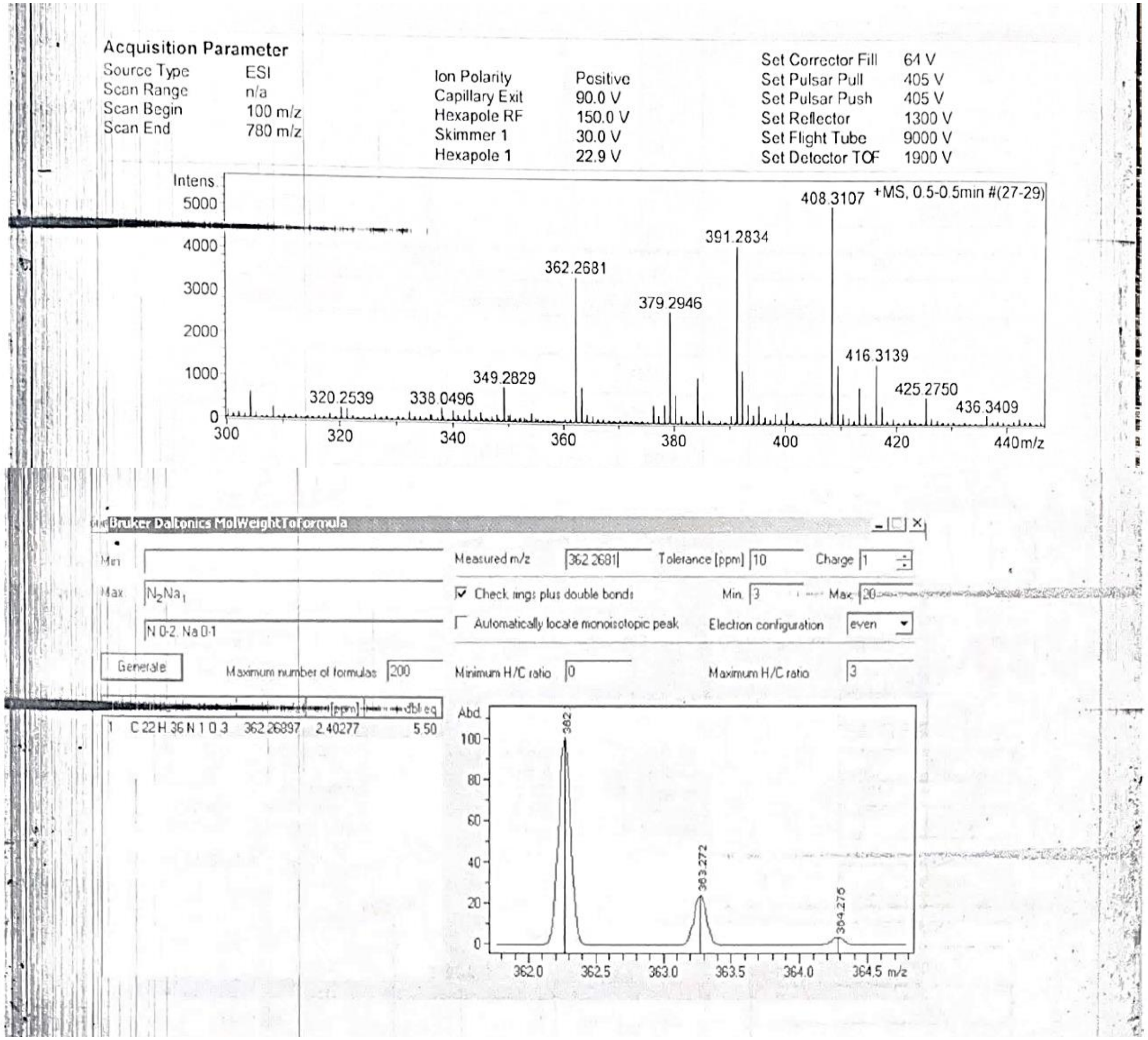

Supplement: Supplementary file 1 — Supplementary Information. [file 41598_2023_40669_MOESM1_ESM.pdf]
